# Epigenetic and oncogenic inhibitors converge to drive a metabolic catastrophe in castration-resistant prostate cancer

**DOI:** 10.1172/JCI203835

**Published:** 2026-06-09

**Authors:** Rhea Sahu, Miriam Enos, Swastika Sharma, Amy E. Schade, Alycia Gardner, Akiko Yoshinaga, Alexandra Indeglia, Eleanor Minogue, Songhua Hu, Kiran Kurmi, Shakchhi Joshi, Daniel R. Schmidt, Samkyu Yaffe, Van T.M. Nguyen, Fang Xie, Steven P. Balk, Matthew G. Vander Heiden, Kristian Helin, Marcia C. Haigis, Karen Cichowski

**Affiliations:** 1 Genetics Division, Department of Medicine, Brigham and Women’s Hospital, Boston, Massachusetts, USA.; 2 Department of Medicine and; 3 Department of Cell Biology, Harvard Medical School, Boston, Massachusetts, USA.; 4 Koch Institute for Integrative Cancer Research and Department of Biology, Massachusetts Institute of Technology, Cambridge, Massachusetts, USA.; 5 Department of Medical Oncology, Dana-Farber Cancer Institute, Boston, Massachusetts, USA.; 6 Division of Cancer Biology, The Institute of Cancer Research, London, United Kingdom.; 7 Division of Medical Oncology and Cancer Center, Beth Israel Deaconess Medical Center, and; 8 Ludwig Center at Harvard, Harvard Medical School, Boston, Massachusetts, USA.

**Keywords:** Cell biology, Metabolism, Oncology, Cancer, Signal transduction, Therapeutics

## Abstract

Men with advanced prostate cancer are typically treated with androgen deprivation therapy, but most ultimately develop resistance and incurable disease (e.g., castration-resistant prostate cancer, CRPC). The majority of CRPCs overexpress the epigenetic enzyme EZH2 and harbor alterations in the PI3K pathway, providing 2 targetable pathways outside of the androgen receptor. Here, we show that EZH2 inhibitors synergize with PI3K, AKT, or mTORC1 inhibitors to kill CRPC in vitro and promote tumor regression in vivo. Strikingly, these agents trigger a catastrophic energy crisis by cooperatively suppressing glycolysis, the TCA cycle, and oxidative phosphorylation before cell death. EZH2 and PI3K pathway inhibitors achieve this by respectively inhibiting 2 key regulators of metabolism, MYC and HIF-1A, while derepressing a proapoptotic stress sensor. Together, these studies reveal a promising therapeutic strategy for CRPC and demonstrate how metabolic plasticity can be fatally impaired by cotargeting upstream oncogenic nodes that converge on this important process.

## Abstract 2

This study describes a promising therapeutic strategy for advanced prostate cancer that triggers metabolic collapse of tumors by combining agents suppressing epigenetic and oncogenic enzymes

## Graphical abstract

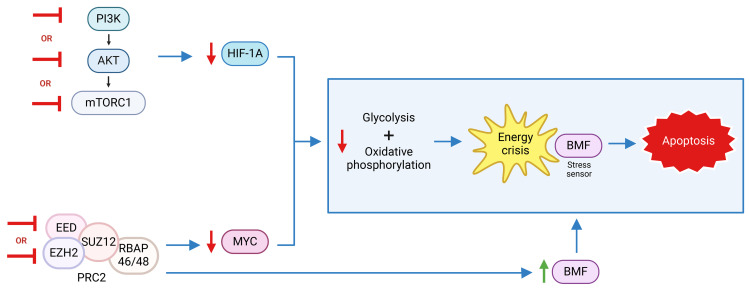

## Introduction

Prostate cancer is the second leading cause of cancer-related deaths in men, with 350,000 individuals dying every year globally (1, 2). While screening and early detection have improved outcomes, 20%–50% of men relapse within 10 years (3). Those diagnosed with metastatic disease have a much poorer prognosis, with a 5-year survival rate of only 30% (1, 2). Men with advanced disease are treated with medical castration, androgen deprivation therapy, and second-generation androgen receptor (AR) signaling inhibitors, such as enzalutamide (2, 3). However, most patients eventually develop resistance and castration-resistant prostate cancer (CRPC), which is lethal. Thus, there is an unmet clinical need to develop more effective treatments for this devastating disease.

The PI3K/AKT signaling pathway is hyperactivated in over 70% of CRPCs (4), with the most common alteration being loss of the negative regulator phosphatase and tensin homolog (*PTEN*) (2, 5). Notably, *PTEN* loss has also been shown to promote AR-independent, castration-resistant disease (6). However, while the PI3K/AKT signaling pathway is frequently hyperactivated in solid tumors, PI3K pathway inhibitors as monotherapies for prostate and other cancers have shown limited efficacy (7–9). Nevertheless, it is unknown whether these agents might be effective in the context of a therapeutic drug combination.

Epigenetic regulators can exert widespread transcriptional changes and have been increasingly implicated in cancer. One such enzyme, EZH2, is overexpressed in 92% of advanced prostate cancer and has been shown to drive metastasis in animal models (10, 11). EZH2 is the catalytic component of the polycomb repressive complex 2 (PRC2), which represses the transcription of specific genes by trimethylation of lysine 27 on histone H3 (H3K27me3) (12). Activating mutations in *EZH2* can occur in a subset of cancers, but *EZH2* is more commonly overexpressed or amplified in solid tumors (12). The EZH2 inhibitor tazemetostat has been FDA approved for the treatment of epithelioid sarcomas and follicular lymphoma (13, 14), and several additional inhibitors of EZH2 and EED, another obligate PRC2 component, are currently under clinical investigation (12, 15). However, as single agents, EZH2 inhibitors also exhibit limited activity in preclinical models of prostate cancer (as shown herein).

In this study, we show that combined suppression of EZH2 and various PI3K pathway components effectively kill CRPC and promote the regression of robust tumor models in vivo, which is exceedingly rare. Moreover, these agents elicit this response by converging on 2 major metabolic pathways and triggering a catastrophic energy crisis, which is not observed in normal cells or unrelated tumors. Indeed, metabolic plasticity is a particularly important feature of prostate cancer development and progression (16–18). Specifically, normal prostate tissue initially relies on basal glycolysis for energy, because healthy prostate glands secrete citrate into the prostatic fluid rather than utilizing it for the TCA cycle, whereas in early-stage prostate cancer, oncogenic AR signaling catalyzes events that restore the TCA cycle, triggering a reliance on oxidative phosphorylation. A third transition to aerobic glycolysis as tumors become castration resistant underscores the importance of metabolic adaptation in this tissue/disease (16–22). Here we report that EZH2 and PI3K pathway inhibitors exert their effects by cooperatively suppressing both glycolysis and oxidative phosphorylation in CRPC, through distinct effectors. Notably, many ongoing discovery efforts strive to target metabolic vulnerabilities in cancer, but these strategies are often hampered by rapid metabolic rewiring. The findings presented here reveal a promising approach to overcome this challenge by cotargeting converging oncogenic/epigenetic nodes, thereby restricting metabolic plasticity. These combinations also provide alternative therapeutic strategies for CRPC that do not rely on the androgen receptor.

## Results

### EZH2 and PI3K/AKT/mTOR complex 1 inhibitors synergize to kill advanced prostate cancer in vitro.

**Figure 1:**
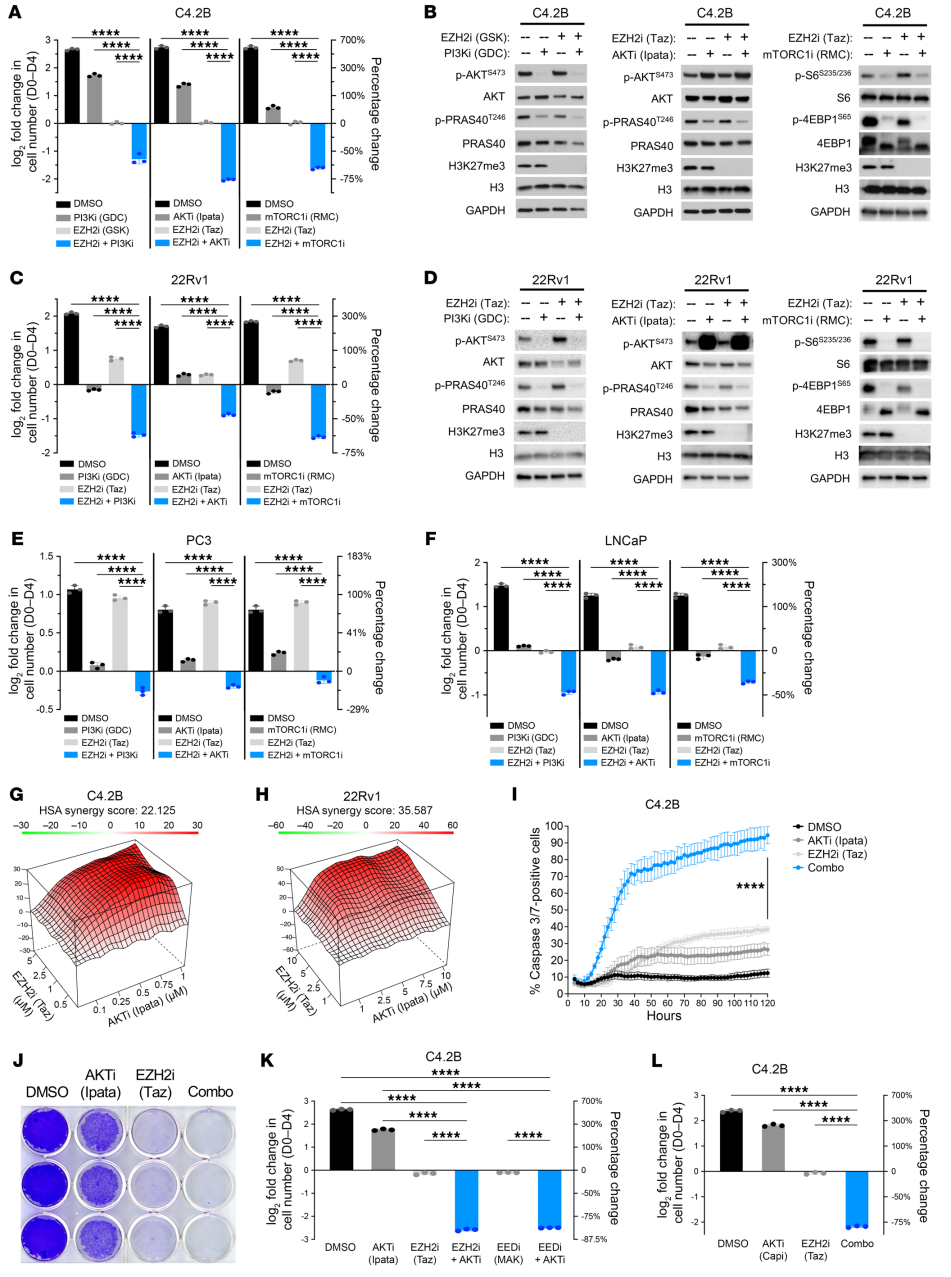
EZH2 and PI3K signaling pathway inhibitors synergize to kill advanced prostate cancer in vitro. (**A**, **C**, **E**, and **F**) Graphs depicting log_2_ fold-change of cell number after 4 days of combined treatment with EZH2 inhibitor (EZH2i) and PI3K/AKT/mTORC1 inhibitor in C4.2B (**A**), 22Rv1 (**C**), PC3 (**E**), and LNCaP (**F**) cells. *n* = 3. Note: AKT inhibitor and mTORC1 inhibitor experiments for LNCaP and PC3 (**E** and **F**) were performed in parallel and share the same DMSO and EZH2i data. EZH2 inhibitors: GSK-126 (GSK), tazemetostat (Taz). PI3K pathway inhibitors: PI3K inhibitor GDC-0941 (GDC), AKT inhibitor ipatasertib (Ipata), and mTORC1 inhibitor RMC-5552 (RMC). (**B** and **D**) Immunoblots confirming PI3K signaling (p-AKT, p-PRAS40, p-S6, p-4EBP1) and EZH2 (H3K27me3) target inhibition with relevant loading controls after 9 hours of treatment in C4.2B (**B**) and 22Rv1 (**D**). (**G** and **H**) Plot depicting synergy between EZH2 and AKT inhibitors using the HSA model of synergy in C4.2B (**G**) and 22Rv1 (**H**). Score > 10 indicates synergy. *n* = 2. (**I**) Graph depicting percentage of C4.2B cells undergoing apoptosis over time, as determined by a caspase-3/7 reporter and Incucyte imaging. *n* = 4 for AKTi and EZH2i; *n* = 5 for DMSO and combo. (**J**) Crystal violet staining of C4.2B cells after 3 weeks of cotreatment with EZH2 and AKT inhibitors. *n* = 3. (**K**) Graph depicting log_2_ fold-change of cell number after 4 days of combined treatment with EZH2i tazemetostat or EED inhibitor MAK683 (MAK) with AKT inhibitor ipatasertib in C4.2B. *n* = 3. (**L**) Graph depicting log_2_ fold-change of cell number after 4 days of combined treatment with EZH2i tazemetostat and AKT inhibitor capivasertib (Capi) in C4.2B. *n* = 3. Error bars represent ± SD, and *P* values were determined using 1-way ANOVA and Tukey’s test. ****P < 0.0001.

To evaluate the effects of EZH2 and PI3K pathway inhibitors in advanced prostate cancer, we first assessed a variety of agents in CRPC models in vitro. Importantly, all of these models harbor PI3K pathway alterations and equivalently overexpress EZH2 (Supplemental Figure 1, A and B; supplemental material available online with this article; https://doi.org/10.1172/JCI203835DS1). Cells were first treated with an EZH2 inhibitor, either GSK-126 or tazemetostat, for 6 days to permit the turnover and loss of H3K27me3. This was followed by the addition of drugs that target different components of the PI3K pathway: the pan-PI3K inhibitor GDC-0941, the AKT inhibitor ipatasertib, or the mTOR complex 1 (mTORC1) inhibitor RMC-5552 (9, 23) (treatment scheme shown in Supplemental Figure 1C). In all cases, previously published drug concentrations that effectively suppress their targets but exert minimal cytotoxic effects were used for these studies. In the *PTEN-*null C4.2B model, each agent individually reduced proliferation to some extent; however, a dramatic 50%–75% net loss of cells was observed only when PI3K pathway and EZH2 inhibitors were combined (Figure 1A). GDC-0941 was primarily included as an investigational tool to broadly probe the PI3K pathway; however, GDC-0941, ipatasertib, and RMC-5552 each exerted a similar response when combined with an EZH2 inhibitor. Western blots confirmed effective target inhibition by PI3K, AKT, mTORC1, and EZH2 inhibitors (Figure 1B).

The 22Rv1 CRPC model does not harbor defects in *PTEN* but instead possesses a phosphatidylinositol-4,5-bisphosphate 3-kinase catalytic subunit alpha (*PIK3CA*) mutation. In this context PI3K pathway inhibitors were more effective at slowing proliferation than EZH2 inhibitors, but again only the combined inhibition of EZH2 and a PI3K pathway component resulted in a potent loss of cells after treatment (Figure 1, C and D). Interestingly, EZH2 and PI3K pathway inhibitors also cooperatively killed the AR-negative, castration-resistant PC3 cell model, albeit less well, suggesting that these cytotoxic effects are not dependent on AR (Figure 1E and Supplemental Figure 1D). Of note, these combinations were also effective in androgen-sensitive prostate cancer cells (LNCaP) (Figure 1F and Supplemental Figure 1E). Importantly, however, these agents were not generally cytotoxic, as EZH2 and AKT inhibitors exerted minimal effects on immortalized prostate epithelial cells and other unrelated cell lines lacking EZH2 and PI3K pathway defects (Supplemental Figure 1F).

Responses to combined EZH2/AKT or EZH2/PI3K inhibitors were synergistic, as reflected by the highest single agent (HSA) synergy model with scores > 10 in multiple cell lines (24) (Figure 1, G and H, and Supplemental Figure 1G). To determine whether cells were dying by apoptosis, Incucyte live-cell imaging (Sartorius) was performed with a caspase-3/7 reporter. Strikingly, combined EZH2 and AKT inhibitors triggered apoptosis in approximately 70% of cells within 40 hours, with apoptosis increasing and killing over 85% of cells in 4–5 days (Figure 1I). These responses were durable for ≥3 weeks, as illustrated by crystal violet staining of drug-treated cells (Figure 1J). Similar effects were observed when the EZH2 inhibitor was replaced with the EED inhibitor MAK683 (25), which inhibits another obligate component of the PRC2 complex (Figure 1K and Supplemental Figure 1H). The EZH2 inhibitor tazemetostat also cooperated with capivasertib, an FDA-approved AKT inhibitor (26) (Figure 1L).

### EZH2 and PI3K/AKT pathway inhibitors promote tumor regression in CRPC models in vivo.

**Figure 2:**
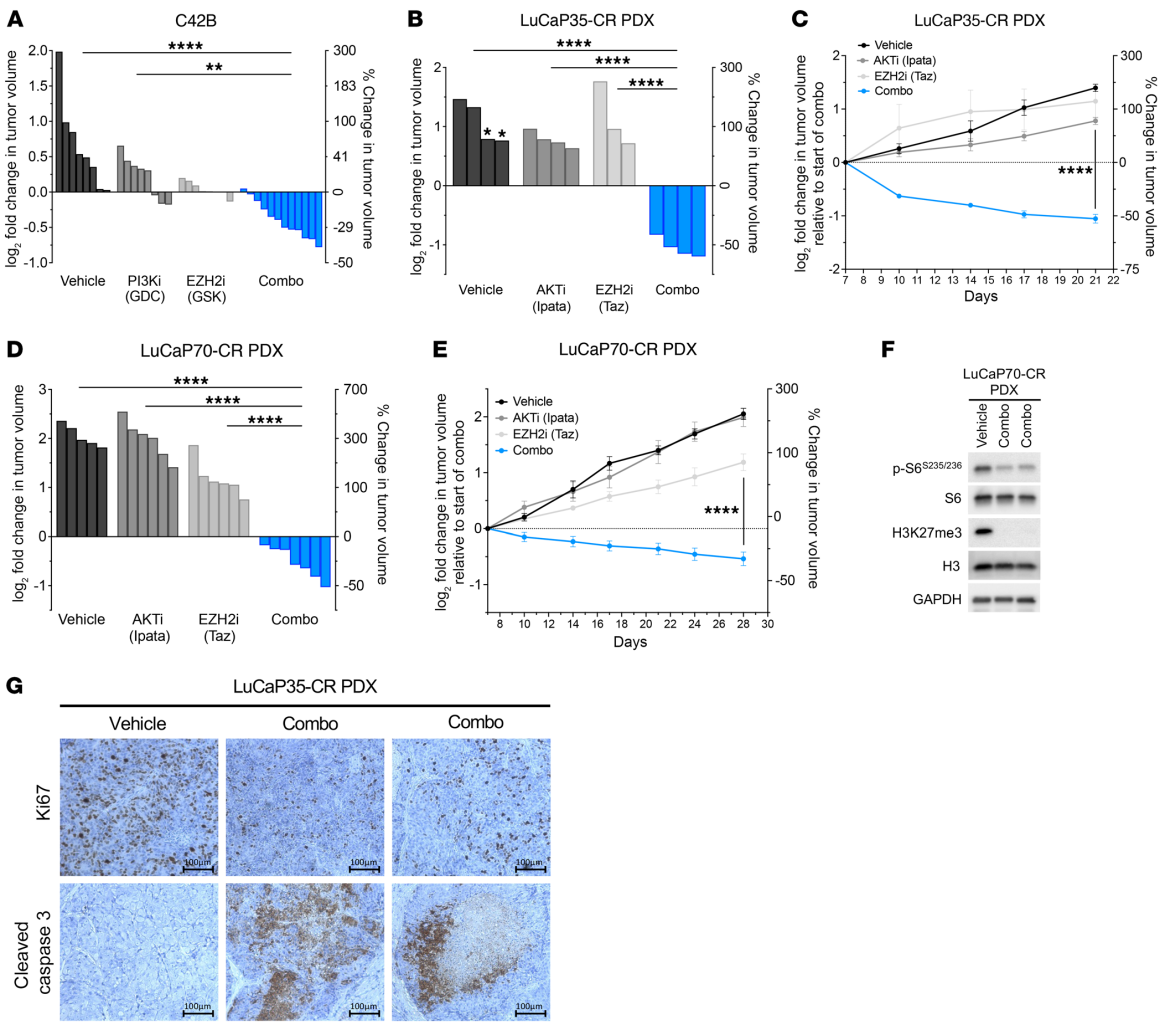
EZH2 and PI3K pathway inhibitors promote the regression of CRPC tumors in vivo. (**A**) Waterfall plot depicting log_2_ fold-change in tumor volume of established C4.2B xenografts in response to vehicle, EZH2 inhibitor GSK-126, and/or PI3K inhibitor GDC-0941 at endpoint (18–20 days, with 7 days of pretreatment with EZH2 inhibitor). (**B** and **C**) Waterfall plot at endpoint (**B**) or line graph (**C**) depicting log_2_ fold-change in tumor volume of established LuCaP35-CR PDX tumors treated with vehicle, EZH2 inhibitor tazemetostat, and/or AKT inhibitor ipatasertib (21 days, with 7 days of pretreatment with EZH2 inhibitor). * Denotes vehicle-treated mice that died on day 17 of study. (**D** and **E**) Waterfall plot at endpoint (**D**) or line graph (**E**) depicting log_2_ fold-change in tumor volume of established LuCaP70-CR PDX tumors treated with vehicle, tazemetostat, and/or ipatasertib at endpoint (28 days, with 7 days of pretreatment with EZH2 inhibitor). (**F**) Immunoblot confirms PI3K signaling and EZH2 target inhibition with relevant loading controls in 2 independent combination-treated LuCaP70-CR PDX tumors after 3 days of cotreatment versus tumors from vehicle-treated mice. (**G**) Immunohistochemistry images showing a decrease in the proliferation marker Ki67 and an increase in the apoptotic marker cleaved caspase-3 in 2 independent combination-treated tumors after 3 days of treatment with EZH2 and AKT inhibitors in LuCaP35-CR PDX tumors. Error bars represent ± SEM, and *P* values were determined using 1-way ANOVA and Tukey’s test. **P < 0.01; ****P < 0.0001.

We next evaluated these agents in cell and patient-derived xenograft tumor models. Similar to the design of in vitro studies, established tumors were pretreated for 1 week with EZH2 inhibitors, followed by the addition of vehicle or PI3K pathway inhibitors. The EZH2 inhibitor GSK-126 and the pan-PI3K inhibitor GDC-0941 were first evaluated in C4.2B xenografts, as a proof-of-principle study. While GSK-126 and GDC-0941 each slowed the growth of tumors, only the combined agents promoted tumor regression (Figure 2A).

Patient-derived CRPC tumor models were then selected for further study with more clinically relevant agents. Specifically, the EZH2 inhibitor tazemetostat and the AKT inhibitor ipatasertib were evaluated in LuCaP35-CR and LuCaP70-CR patient-derived xenograft (PDX) models, which both harbor alterations in *PTEN* and express high levels of EZH2 (27). In each of these models, tazemetostat and ipatasertib alone exerted minimal effects. However, LuCaP35-CR tumors shrank on average by 51.6% in response to both agents (Figure 2, B and C), and LuCaP70-CR tumors also regressed on average by 29.7% (Figure 2, D and E). These combinations were well tolerated in mice, as assessed by body condition scoring and weight loss (Supplemental Figure 2). Immunoblots derived from tumors on treatment confirmed effective target inhibition in vivo (Figure 2F). Additionally, tumors exposed to EZH2 and AKT inhibitors exhibited a decrease in proliferation, demonstrated by reduced anti-Ki67 staining, and an increase in apoptosis, marked by cleaved caspase-3 expression, within 3 days of combined treatment (Figure 2G). Thus, EZH2 and PI3K pathway inhibitors together promote the regression of robust CRPC models in vivo.

### EZH2 and PI3K pathway inhibitors cooperatively suppress glycolysis and oxidative phosphorylation, resulting in a catastrophic energy crisis.

**Figure 3:**
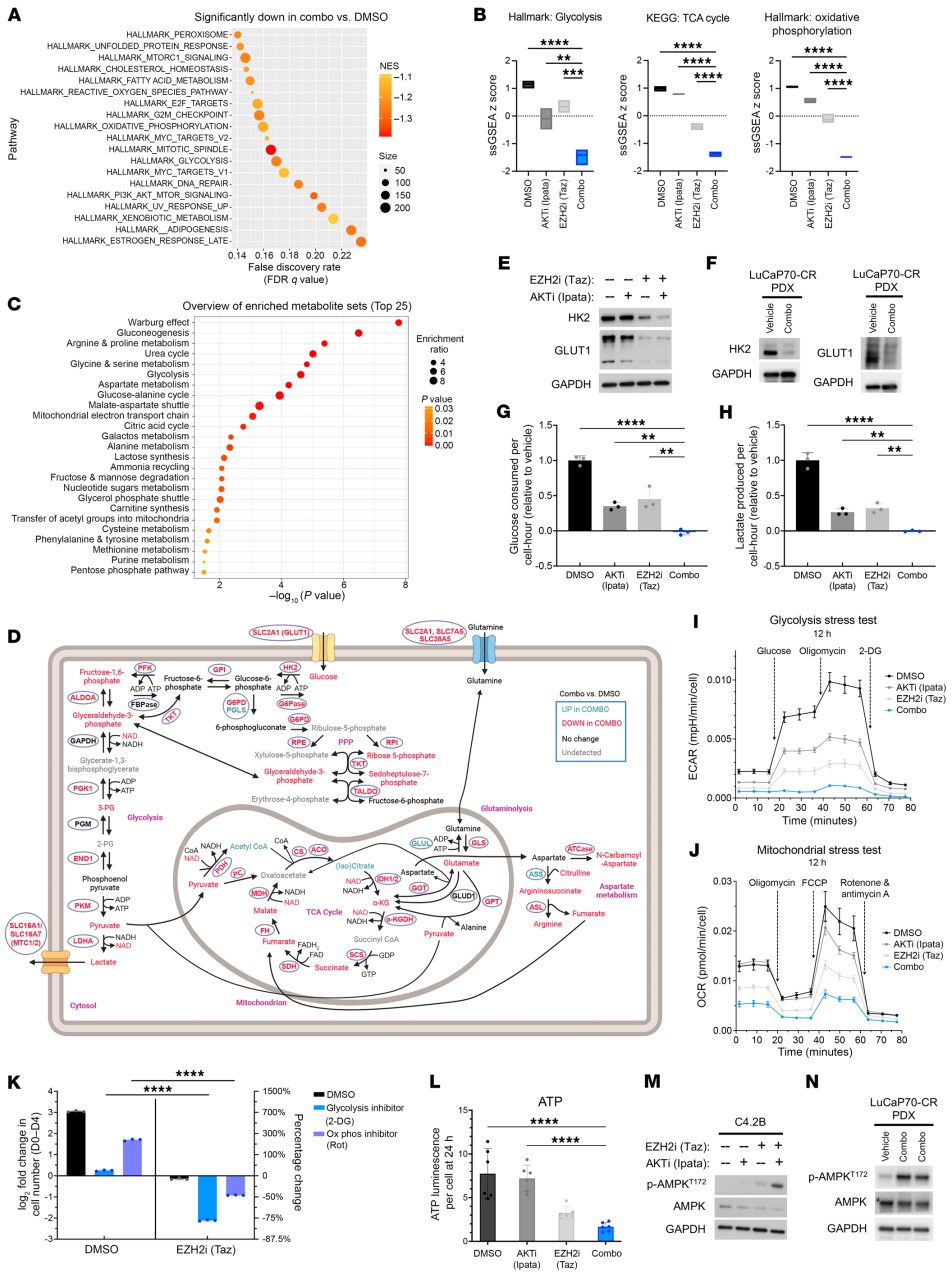
EZH2 and PI3K pathway inhibitors cooperatively suppress glycolysis and oxidative phosphorylation, triggering a catastrophic energy crisis. (**A**) RNA-seq GSEA depicting significantly downregulated Hallmark gene sets in C4.2B treated for 24 hours with combination of EZH2i tazemetostat and AKTi ipatasertib compared with DMSO (FDR < 0.25). *n* = 3. (**B**) ssGSEA highlights cooperative suppression of glycolysis, TCA cycle, and oxidative phosphorylation. KEGG, Kyoto Encyclopedia of Genes and Genomes. (**C**) Plot of steady-state metabolomics data showing top 25 pathways that decrease after 6 hours of combo in C4.2B. *n* = 3. (**D**) Metabolism map comparing gene expression of enzymes (circled) and metabolite levels in glycolysis and TCA cycle in C4.2B. Red indicates genes/metabolites that decrease; blue marks genes/metabolites that increase with combo. Created using BioRender. (**E** and **F**) Immunoblot showing decrease of HK2 and GLUT1 within 9 hours of combo in C4.2B (**E**) and after 2–3 days of combo in LuCaP70-CR PDX (**F**). (**G** and **H**) Graph depicting relative changes in glucose (**G**) and lactate (**H**) in C4.2B culture media after 24 hours of combo. *n* = 3. (**I** and **J**) Graphs of Seahorse glycolysis stress test (**I**) and mitochondrial stress test (**J**) normalized to C4.2B cell counts after 12 hours of combo. Error bars represent ± SEM. *n* = 7 for DMSO and AKTi, *n* = 8 for EZH2i and combo. ECAR, extracellular acidification rate. OCR, oxygen consumption rate.(**K**) Log_2_ fold-change of cell number over 4 days of treatment with glycolysis inhibitor 2-DG or oxidative phosphorylation inhibitor rotenone with EZH2i in C4.2B. *n* = 3. (**L**) Graph of ATP produced per C4.2B cell after 24 hours of EZH2i and AKTi. *n* = 6. (**M** and **N**) Immunoblot depicting p-AMPK induction after 24 hours of combo in C4.2B (**M**) and 4 days of combo in LuCaP70-CR PDX (**N**). Unless otherwise noted, error bars represent ± SD. *P* values were determined using 1-way ANOVA and Tukey’s test. **P < 0.01; ***P < 0.001; ****P < 0.0001.

To elucidate the mechanism of action, we performed RNA-seq in cells treated with tazemetostat, ipatasertib, or both agents after 24 hours, before cell death. An unbiased analysis of transcriptional profiles using gene set enrichment analysis (GSEA) (28–30) demonstrated that the top signatures were associated with multiple metabolic pathways, including glycolysis and oxidative phosphorylation, which were potently suppressed in response to combined EZH2 and AKT inhibitors (Figure 3A). Additionally, we observed a substantial decrease in target genes of MYC, a transcription factor known to critically regulate glycolysis, oxidative phosphorylation, and amino acid metabolism (31–34) (Figure 3A). Single sample GSEA (ssGSEA) analysis of transcriptional profiles from all 4 treatment arms further revealed that EZH2 and AKT inhibitors cooperatively suppressed genes associated with glycolysis, the TCA cycle, and oxidative phosphorylation in CRPC cells (Figure 3B). Transcriptional heatmaps illustrating the cooperative effects on individual genes within these pathways are shown in Supplemental Figure 3A. Similar results were obtained when cells were treated with PI3K inhibitor GDC-0941 and EZH2 inhibitor GSK-126 (Supplemental Figure 3B).

To determine whether these transcriptional events were accompanied by metabolic changes, steady-state metabolomics was performed 6 hours after combined treatment with EZH2 and AKT inhibitors (note that cells were pretreated with EZH2 inhibitors where indicated). Strikingly, even at this early time point, dramatic changes in glycolysis, the TCA cycle, and the electron transport chain were observed in cells treated with both agents (Figure 3C). To summarize, a cartoon depicting the broad suppression of various metabolites together with the transcriptional suppression of regulatory genes is shown in Figure 3D, where all suppressed metabolites (listed) and genes (circled) are depicted in red. Western blots confirmed that EZH2 and AKT inhibitors rapidly suppressed the expression of HK2, the rate-limiting enzyme of glycolysis, and GLUT1, the major glucose transporter (32) (Figure 3E and Supplemental Figure 3C). Specifically, EZH2 inhibition alone suppressed GLUT1 whereas EZH2 and AKT inhibitors cooperatively decreased the expression of HK2 (Figure 3E and Supplemental Figure 3C). Importantly, both HK2 and GLUT1 expression were also potently suppressed in PDX tumor tissue after only 2–3 days of treatment with the combination in vivo (Figure 3F).

The decrease in GLUT1 and HK2 expression, as well as the potent reduction in glycolysis and oxidative phosphorylation genes and metabolites, prompted us to measure changes in glycolysis and mitochondrial respiration. Indeed, together EZH2 and AKT inhibitors cooperatively blocked glucose uptake (Figure 3G) and lactate secretion (Figure 3H), demonstrating that glucose is not being utilized for glycolysis. Seahorse glycolysis and mitochondrial stress tests (Agilent) further confirmed that EZH2 and AKT inhibitors cooperatively suppressed both glycolysis and mitochondrial respiration at early time points before cell death (Figure 3, I and J, and Supplemental Figure 3, D and E). To determine whether we could prevent cell death by merely bypassing the suppressive effects on glucose uptake/glycolysis, we added exogenous pyruvate. However, the addition of pyruvate did not alter the cytotoxic effect of EZH2 and AKT inhibitors (Supplemental Figure 3F), consistent with the observation that these agents also independently suppress genes that regulate the TCA cycle and oxidative phosphorylation.

To confirm a metabolic role for EZH2 inhibition, tazemetostat was combined with either the glycolysis inhibitor 2-deoxy-d-glucose (2-DG) or an oxidative phosphorylation inhibitor, rotenone. Although these metabolic agents alone exerted cytostatic or partially cytostatic effects, when either agent was combined with tazemetostat, potent cytotoxic responses were observed (Figure 3K). Consistent with a collapse of cellular energy metabolism, ATP levels were potently suppressed in response to EZH2 and AKT inhibitors within 24 hours (Figure 3L). In addition, AMPK, a sensor of low ATP levels and therefore low cellular energy, was activated by phosphorylation, both in vitro (Figure 3M) and in vivo in tumors treated with both drugs (Figure 3N). Together, these data demonstrate that while EZH2 and AKT inhibitors each modestly reduce energy metabolism, when combined they drive a catastrophic energy crisis and ultimately cell death in CRPC.

Metabolic assays and steady-state metabolomics all revealed that combined EZH2 and AKT inhibitors exerted profound effects on metabolism within 6 hours (Figure 3, C and D, and Supplemental Figure 3, D and E), well before the initiation of apoptosis (Figure 1I), supporting the possibility that metabolic suppression could be a cause rather than an effect of cell death. Nevertheless, we sought to confirm that inhibition of metabolic transcriptional signatures was not a general feature of dying cells. Importantly, these signatures were not suppressed when EZH2 inhibitors were combined with MEK inhibitors, which killed colorectal cancer models (Supplemental Figure 3G) (35). Thus, the cooperative inhibition of these major metabolic pathways is not a general feature of cell death, even in the context of other EZH2 inhibitor–based combinations (35).

### EZH2 and PI3K/AKT inhibitors function by suppressing major metabolism regulators: MYC and HIF-1A.

**Figure 4:**
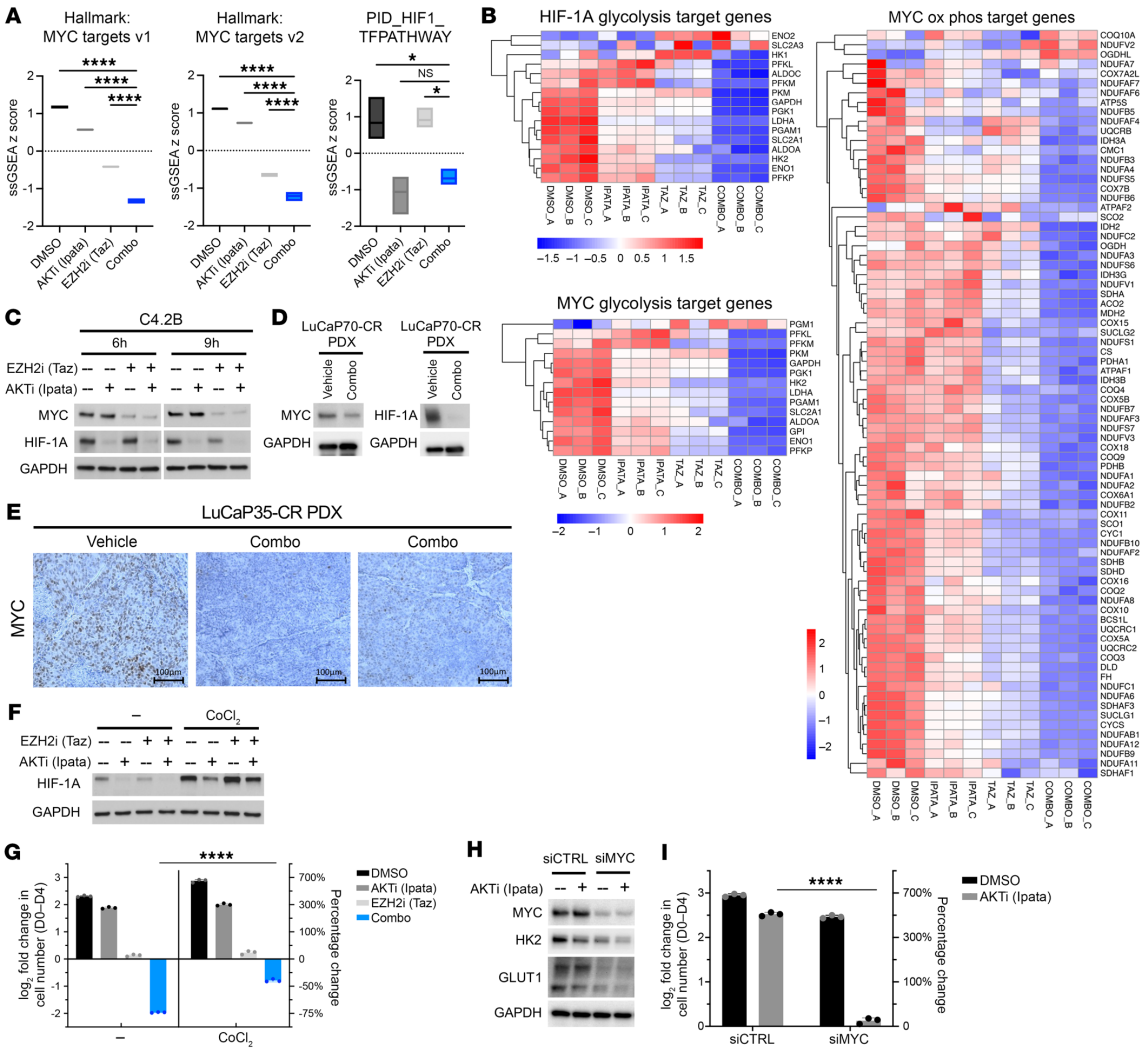
EZH2 and AKT inhibitors function by suppressing major metabolism regulators: MYC and HIF-1A. (**A**) RNA-seq ssGSEA in C4.2B cells shows that HIF-1A and MYC transcriptional targets are suppressed by AKTi ipatasertib and EZH2i tazemetostat. *n* = 3. (**B**) Heatmaps depicting changes in gene expression of known MYC and HIF-1A glycolysis and oxidative phosphorylation target genes in response to EZH2 and AKT inhibitors. *n* = 3. (**C**) Immunoblot highlighting decrease of MYC with EZH2 inhibition and HIF-1A with AKT inhibition in C4.2B after 6 and 9 hours of combination treatment. GAPDH is used as a loading control. (**D**) Immunoblot showing decrease of MYC and HIF-1A in LuCaP70-CR PDX tumor tissue after 2–3 days of combination treatment. Each lane indicates an independent tumor. Note: these blots are from the same experiment and have the same loading controls as Figure 3F. (**E**) Immunohistochemistry images of LuCaP35-CR PDX tumor tissue depicting decrease in MYC staining in 2 independent tumors after 3 days of combination treatment with EZH2 and AKT inhibitors. (**F** and **G**) Immunoblot after 9 hours (**F**) and bar graph showing log_2_ fold-change of cell number after 4 days (**G**) of cotreatment with EZH2i and AKTi in C4.2B cells cultured in media with or without cobalt chloride (CoCl_2_) to stabilize HIF-1A protein levels. *n* = 3. (**H** and **I**) Immunoblot after 24 hours (**H**) and graph depicting log_2_ fold-change in cell number after 4 days (**I**) of treatment with AKTi in C4.2B cells with or without knockdown of MYC. *n* = 3. Error bars represent ± SD, and *P* values were determined using 1-way ANOVA and Tukey’s test. * *P* < 0.05; **** *P* < 0.0001; NS, *P* > 0.05 or not significant.

Given the broad effects of these agents on metabolism and their regulatory genes, we considered transcription factors that are known to regulate metabolism. As shown in Figure 3A, MYC target signatures were substantially decreased in cells treated with EZH2 and AKT inhibitors. Notably, MYC regulates both glycolysis and oxidative phosphorylation genes (31–34), and MYC overexpression has been shown to enhance glycolysis (36) and oxidative phosphorylation (37) in prostate cancer. We also considered HIF-1A, another transcription factor known to regulate most glycolytic genes (38). In fact, HIF-1A and MYC overexpression have both been associated with aggressive prostate cancer (37, 39–42). ssGSEA revealed that MYC and HIF-1A signatures were both suppressed by EZH2 and AKT inhibitors (Figure 4A). Interestingly, MYC transcriptional signatures were more potently inhibited by EZH2 inhibition, whereas HIF-1A target gene signature was primarily suppressed by the AKT inhibitor. Importantly, these transcriptional changes included the potent suppression of glycolysis and oxidative phosphorylation target genes of HIF-1A and MYC (Figure 4B). Immunoblotting further demonstrated that EZH2 inhibition alone was sufficient to inhibit MYC protein expression and the AKT inhibitor was sufficient to inhibit HIF-1A protein expression in multiple prostate cancer cell lines (Figure 4C and Supplemental Figure 4A).

PI3K pathway activation is well known to regulate HIF-1A expression, largely by enhancing its translation (43), consistent with the suppressive effect of PI3K/AKT inhibitors on HIF-1A protein expression in CRPC (Figure 4C and Supplemental Figure 4A). Accordingly, mTORC1 inhibition also suppressed HIF-1A expression in CRPC (Supplemental Figure 4B). However, MYC suppression is not a general response to EZH2 inhibition and is not observed in colorectal cancer, triple-negative breast cancer, or other noncancerous cells (Supplemental Figure 4C). Nevertheless, immunoblots and immunohistochemical analysis confirmed the potent decrease in both MYC and HIF-1A in PDX tumor tissue treated with EZH2 and AKT inhibitors, before tumor regression (Figure 4, D and E), consistent with the notion that concomitant suppression of these 2 major metabolic regulators, HIF-1A and MYC, may contribute to the therapeutic response in CRPC.

To first investigate whether HIF-1A suppression was required, cells were treated with cobalt chloride, which stabilizes HIF-1A by preventing its hydroxylation and subsequent recognition by the ubiquitin ligase VHL (44). Cobalt chloride partially stabilized HIF-1A in the presence of EZH2 and AKT inhibitors (Figure 4F) and decreased the sensitivity of cells to this therapeutic combination (Figure 4G). We were unable to ectopically express MYC in these cells using multiple methods, in part, due to its known toxicity when overexpressed in many settings. Therefore, to determine whether the decrease in MYC by EZH2 inhibitor might be contributing to the therapeutic response, we instead ablated MYC expression with pooled siRNA sequences. We initially hypothesized that MYC knockdown might recapitulate the effects of tazemetostat and cooperate with ipatasertib to suppress metabolic genes and kill cells. Indeed, MYC ablation inhibited the expression of GLUT1 and cooperatively suppressed the expression of HK2, similar to our previous observations (Figure 4H). Moreover, while MYC or AKT suppression alone exerted minimal effects on proliferation, together they exerted a profound cytostatic response (Figure 4I). Nevertheless, combined suppression of MYC and AKT did not recapitulate the robust cytotoxic response conferred by EZH2 and AKT inhibitors (Figure 4G and throughout this study). Additional metabolic studies suggested that tazemetostat may exert slightly greater effects than MYC knockdown in this therapeutic context, which could contribute to the enhanced response (Supplemental Figure 4D); however, we also considered the possibility that additional nonmetabolic targets of EZH2 might also play a role in driving apoptosis.

### EZH2 inhibition concomitantly derepresses a sensor of cellular stress that drives apoptosis.

**Figure 5:**
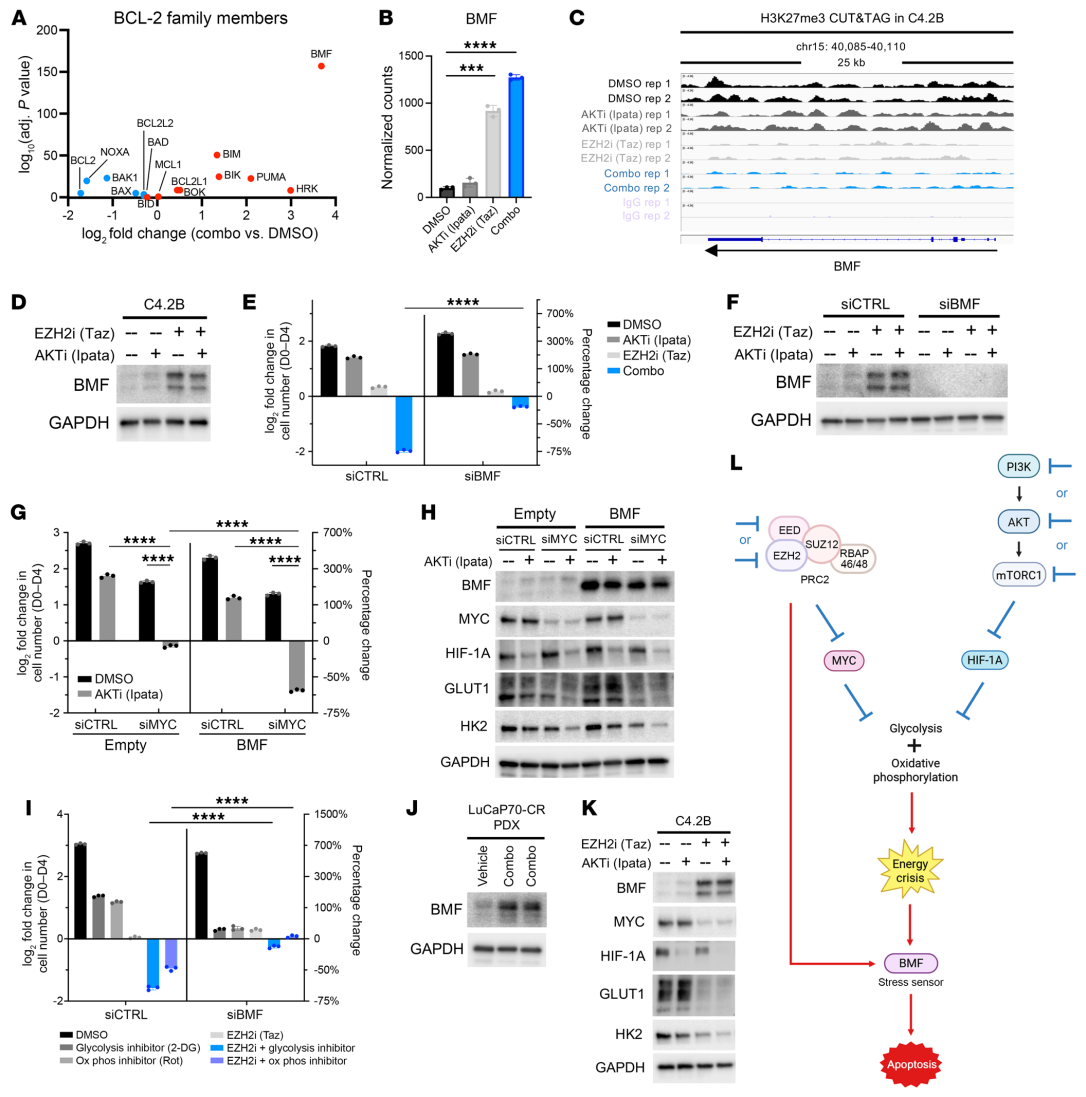
EZH2 inhibition concomitantly derepresses a sensor of cellular stress that drives apoptosis. (**A**) Volcano plot comparing expression of BCL-2 family members in C4.2B treated with combination of EZH2i tazemetostat and AKTi ipatasertib compared with DMSO. (**B**) Normalized RNA-seq counts of proapoptotic protein BMF in C4.2B cells treated with EZH2i and/or AKTi. *n* = 3. Significance determined by DESeq2 adjusted *P* value. (**C**) CUT&TAG reveals loss of H3K27me3 along the BMF gene in EZH2i-treated C4.2B. *n* = 2. (**D**) Immunoblot depicting induction of BMF with EZH2i, with or without AKTi, after 24 hours of combo in C4.2B. (**E**) Log_2_ fold-change in cell number after 4 days of combo in C4.2B, with or without BMF knockdown. *n* = 3. (**F**) Immunoblot confirms BMF knockdown after 24 hours of treatment. (**G**) Log_2_ fold-change in cell number after 4 days of AKTi in C4.2B cells that express BMF and have MYC knocked down via siRNA (and relevant controls). *n* = 3. (**H**) Immunoblot confirms BMF expression, MYC knockdown, GLUT1 suppression, and HIF-1A suppression by AKTi after 24 hours. (**I**) Log_2_ fold-change in cell number after 4 days of treatment with glycolysis inhibitor 2-DG or oxidative phosphorylation inhibitor rotenone with EZH2i in C4.2B — with or without BMF knockdown. *n* = 3. (**J**) Immunoblot showing induction of BMF in LuCaP70-CR PDX tumors after 4 days of cotreatment with EZH2i and AKTi. Note: these blots are from the same experiment and have the same loading control as Figure 3N. (**K**) Immunoblot showing changes in BMF, MYC, HIF-1A, HK2, and GLUT1 with AKTi and EZH2i in C4.2B. (**L**) Model of cell death caused by inhibitors of the PRC2 complex (EZH2 or EED) combined with inhibitors of the PI3K pathway (PI3K, AKT, or mTORC1) in CRPC. Created using BioRender. Error bars represent ± SD, and *P* values were determined using 1-way ANOVA and Tukey’s test. ***P < 0.001; ****P < 0.0001.

To identify additional signals that might be involved in triggering apoptosis, we examined the expression of Bcl-2 family members. Interestingly, the proapoptotic protein BMF was the most highly upregulated Bcl-2 family member in response to these agents (Figure 5A). BMF is a stress sensor that is normally sequestered in an inactive state; however, in response to a variety of cellular stresses, it binds and neutralizes prosurvival BH3-containing proteins, thereby triggering apoptosis (45, 46). Specifically, we found that BMF was upregulated by more than 10-fold in CRPC models in response to EZH2 or EZH2/AKT inhibition (Figure 5B and Supplemental Figure 5A). CUT&TAG using an H3K27me3 antibody further demonstrated that repressive H3K27me3 marks were present on histones throughout the *BMF* locus (Figure 5C) and at upstream sites (Supplemental Figure 5B), which were lost upon treatment with tazemetostat or the combination (Figure 5C and Supplemental Figure 5B). Accordingly, EZH2 inhibition was sufficient to dramatically increase both BMF mRNA and protein expression (Figure 5, B and D, and Supplemental Figure 5C), suggesting that the PRC2 complex normally represses the BMF locus and that EZH2 inhibitors relieve this repression. To determine if BMF was required for the therapeutic response to EZH2 and AKT inhibitors, BMF was ablated with pooled siRNA sequences. Indeed, BMF suppression prevented the cytotoxicity conferred by EZH2 and AKT inhibitors (Figure 5, E and F). Together these findings demonstrate that BMF upregulation is required for cell death; however, upregulation alone is not sufficient, consistent with the known function and regulation of BMF, which triggers apoptosis but only when it senses cellular stress (45, 46).

We previously reported that EZH2 and histone deacetylase (HDAC) inhibitors can cooperatively kill prostate cancers through the induction of a different stress response gene, activating transcription factor 3 (ATF3) (11). ATF3 was not induced by EZH2 and AKT inhibitors, consistent with the known additional requirement of HDAC suppression for histone deacetylation and transcriptional activation of the ATF3 locus (Supplemental Figure 5D) (11). However, because EZH2 inhibition is sufficient for BMF expression, it was indeed upregulated by EZH2 and HDAC inhibitors (Supplemental Figure 5D). Moreover, BMF ablation also impaired the therapeutic response to these agents (Supplemental Figure 5E), suggesting that ATF3 and BMF collaboratively function in this former therapeutic context and underscoring BMF’s importance in broadly mediating cell death in response to cellular stress in prostate cancer.

Based on these findings, we hypothesized that EZH2 and AKT inhibitors potently suppress glycolysis and oxidative phosphorylation by decreasing MYC and HIF-1A protein levels, respectively, which triggers an energy crisis that BMF senses. Consistent with this model, ectopic expression of BMF was able to kill CRPC cells only when there was both a suppression of MYC (by siRNAs) and a decrease in HIF-1A (by the AKT inhibitor) (Figure 5, G and H). Conversely, when glycolysis or oxidative phosphorylation inhibitors were combined with tazemetostat, BMF ablation prevented the cytotoxic response, supporting the notion that BMF is required to kill cells in response to energy stress (Figure 5I). Importantly, the potent induction of BMF was observed in multiple cell lines (Figure 5D and Supplemental Figure 5C) and in PDX tumors upon treatment in vivo (Figure 5J). This striking increase in BMF was observed alongside the potent suppression of metabolic targets MYC, HIF-1A, GLUT1, and HK2 (Figure 5K). Together, these findings demonstrate that EZH2 and PI3K pathway inhibitors kill CRPC by promoting irresolvable metabolic stress. EZH2 inhibitors concomitantly increase BMF expression, which then acts as a sensor of this energy crisis and triggers apoptosis (Figure 5L).

## Discussion

CRPC remains a highly aggressive and largely incurable disease with limited therapeutic options. In this study, we show that EZH2 inhibitors synergize with PI3K, AKT, or mTORC1 inhibitors to kill advanced CRPCs in vitro and promote tumor regression in vivo. This finding is particularly striking given that tumor regression is rarely observed in CRPC models. Notably, these effects extend across many models, including androgen-independent CRPC (C4.2B, 22Rv1, LuCaP35-CR, LuCaP70-CR), castration-sensitive LNCaP, and AR-negative PC3 cells, suggesting this approach may benefit a broad spectrum of patients with advanced prostate cancer.

Mechanistically, synergy arises from the convergent suppression of key metabolic regulators. Specifically, we show that EZH2 inhibition suppresses MYC expression, while PI3K pathway inhibition suppresses HIF-1A, collectively blocking both glycolysis and oxidative phosphorylation. By simultaneously disrupting both pathways, these agents substantially impair energy production and prevent adaptive switching between glycolysis and oxidative phosphorylation, which can contribute to therapeutic resistance in other settings. Notably, HIF-1A and MYC overexpression have both been linked to resistance to castration and AR inhibitors (37, 39–42). Therefore, these agents provide a powerful means of cotargeting these important yet difficult-to-drug proteins in this aggressive malignancy. Concomitantly, EZH2 inhibition derepresses and upregulates the proapoptotic protein BMF, which triggers apoptosis in the context of acute metabolic crisis. Consistent with its role as a stress-responsive protein and the notion that it is sequestered in an inactive form in the absence of stress, BMF expression alone is insufficient to induce apoptosis but also requires the metabolic deficiencies EZH2 and AKT inhibitors induce.

Notably, the profound metabolic defects observed are not a general consequence of agents that trigger cancer cell death and thus far appear to be restricted to these agents in prostate cancer. This is partly because EZH2 inhibitors do not broadly suppress MYC (and downstream metabolic genes) in other tissues. We also show that MYC suppression and BMF upregulation (both triggered by EZH2 inhibitors) and HIF-1A suppression (caused by PI3K pathway inhibitors) are all necessary and sufficient for CRPC cell death. Importantly, a rapid induction of BMF, concomitant suppression of MYC and HIF-1A, and the consequential activation of AMPK, can be readily observed in PDX tumors at early time points in vivo. Together, these findings reveal a tractable therapeutic vulnerability in CRPC and demonstrate that oncogenes can critically regulate distinct pathways in different tumor types. The dramatic effect that this combination has on metabolism in CRPC raises the intriguing possibility that ¹^8^F-FDG PET may serve as a useful response biomarker in this setting.

PI3K/AKT signaling is activated in over 70% of advanced prostate cancers (4), and EZH2 is overexpressed in more than 90% of metastatic tumor samples (11), highlighting the broad potential utility of this combination in this disease. Importantly, this combination is well tolerated in normal cells and in mice, suggesting that the aberrant activation of these 2 pathways in tumors provides a tractable therapeutic window. Multiple clinical agents that target the PRC2 complex (various EZH2, EZH1/2, and EED inhibitors) (14, 25, 47) and the PI3K pathway (ipatasertib, capivasertib, the mTORC1-selective inhibitor RMC-5552, and the dual mTOR/PI3K inhibitor gedatolisib) (9, 23, 26, 48–50) provide several feasible options for clinical translation. Moreover, this approach does not rely on targeting the androgen receptor.

Together, these findings describe a promising strategy that exploits a metabolic vulnerability in CRPC. By cotargeting EZH2 and the PI3K/AKT/mTORC1 pathway, we show that it is possible to suppress metabolic plasticity, resulting in a severe energy crisis and a selective induction of apoptosis in cancer cells. Importantly, these findings can be used to support the development of clinical trials combining EZH2 and PI3K pathway inhibitors in CRPC, which would offer promising new treatment option(s) for individuals with refractory castration-resistant disease.

## Methods

### Sex as a biological variable.

Prostate cancer occurs in males only, and therefore all mouse studies were conducted using male mice.

### Cell lines.

Cell lines were purchased from ATCC and were regularly tested for Mycoplasma contamination using the MycoAlert Mycoplasma Detection Kit (Lonza, LT07-710). All experiments were performed within 17 passages from thawing cells. C4.2B (ATCC, CRL-3315), 22Rv1 (ATCC, CRL-2505), LNCaP (ATCC, CRL-1740), and RWPE-1 (ATCC, CRL-3607) cells were cultured in RPMI (Corning, 10-040-CV) supplemented with 10% FBS (GeminiBio, 100-106-500) and 1× concentration of penicillin/streptomycin/glutamine (Gibco, 10378016). PC3 (ATCC, CRL-1435) and HEK293T (293T; ATCC, CRL-3216) cells were cultured in DMEM (Corning, 10-013-CV) supplemented with 10% FBS and 1× concentration of penicillin/streptomycin/glutamine. C4.2B, LNCaP, and RWPE-1 cells were switched to 10% charcoal-stripped FBS (Gibco, 12676029), and 22Rv1 and PC3 cells were switched to 2% charcoal-stripped FBS on day 0 (start of combination treatment with EZH2 and PI3K pathway inhibitors). 293T (ATCC, CRL-3216) cells were switched to 2% FBS on day 0. RPMI-7951 melanoma (ATCC, HTB-66) cells were cultured in EMEM (ATCC, 30-2003) with 10% FBS and 1× concentration of penicillin/streptomycin/glutamine. Colon cancer cells were cultured and treated as previously described (35).

### Inhibitors.

EZH2 inhibitor tazemetostat (Selleck Chemicals, S7128) was used at 1 μM in C4.2B and LNCaP cells, 2.5 μM in 22Rv1 and RWPE-1 cells, and 5 μM in PC3 and 293T cells. EZH2 inhibitor GSK-126 (Selleck Chemicals, S7061) was used at 5 μM in C4.2B and RPMI-7951 cells. AKT inhibitor ipatasertib (Selleck Chemicals, S2808) was used at 0.25 μM in C4.2B and LNCaP cells and 5 μM in 22Rv1, PC3, RWPE-1, and 293T cells. PI3K inhibitor GDC-0941 (Selleck Chemicals, S1065) was used at 0.5 μM in C4.2B, LNCaP, and RPMI-7951 cells, 2 μM in 22Rv1 cells, and 5 μM in PC3 cells. mTORC1 inhibitor RMC-5552 (MedChemExpress, HY-132168) was used at 10 nM in C4.2B cells, 1 nM in 22Rv1 and LNCaP cells, and 1 μM in PC3 cells. EED inhibitor MAK683 (Selleck Chemicals, S8983) was used at 1 μM, and AKT inhibitor capivasertib (Selleck Chemicals, S8019) was used at 0.25 μM. HDAC inhibitor vorinostat (MedChemExpress, HY-10221) was used at 2 μM in C4.2B cells as previously described (11). Glycolysis inhibitor (Sigma, D8375) was used at 10 mM, and oxidative phosphorylation inhibitor rotenone (Sigma, R8875) was used at 0.1 μM.

### Proliferation cell counting assay.

Cells were seeded and pretreated the next day with DMSO or EZH2/EED inhibitor (day –6). Cells were passaged on day –3 and maintained in DMSO or EZH2/EED inhibitor. On day –1, cells were seeded in triplicates in 6-well plates at 150,000 cells/well for counting and 650,000 cells per 10 cm plate for protein collection. For PC3 and 293T cells, cells were seeded in triplicates in 6-well plates at 50,000 cells/well for counting and 220,000 cells per 10 cm plate for protein collection. On day 0, cells were switched to charcoal-stripped FBS and dosed with DMSO, EZH2/EED inhibitor, PI3K/AKT/mTORC1 inhibitor, or a combination. For experiments with glycolysis or oxidative phosphorylation inhibitors, 2-DG or rotenone was added on day 0. On day 0 and day 4, 6-well plates were counted using a hematocytometer. Log_2_ fold-change in cell number was calculated to assess proliferation over these 4 days of cotreatment. Protein lysates were collected from 10 cm plates 6–24 hours depending on the experiment. For cobalt chloride experiments, water or 50 μM cobalt (II) chloride hexahydrate (Sigma, C8661) was added to cell culture media starting day –3. For pyruvate experiments, water or 10 mM sodium pyruvate (Sigma, P5280) was added to cell culture media starting day –6. For experiments including siRNA knockdown, cells were incubated with siRNA for 6 hours on day –2. Pooled siRNAs were purchased from Dharmacon: MYC (Horizon Discovery, L-003282-02-0010), BMF (Horizon Discovery, L-004393-00-0010), and nontargeting control (Horizon Discovery, D-001810-10-50).

### Synergy.

Similarly to proliferation cell counting assay, cells were pretreated with EZH2 inhibitor and then seeded in duplicates in white, flat-bottom plates at 5,000 cells/well on day –1. On day 0, they were dosed in charcoal-stripped FBS with EZH2 and PI3K/AKT inhibitors. For C4.2B, the concentration range was EZH2 inhibitor tazemetostat (0, 0.5, 1, 2.5, 5 μM), and AKT inhibitor ipatasertib (0, 0.1, 0.25, 0.5, 0.75, 1 μM); EZH2 inhibitor GSK-126 (0, 1, 2.5, 5 μM), and PI3K inhibitor GDC-0941 (0, 0.1, 0.25, 0.5, 1, 2 μM). For 22Rv1, the concentration range was tazemetostat (0, 1, 2.5, 5, 10 μM) and ipatasertib (0, 1, 2.5, 5, 7.5, 10 μM). After 4 days of combined treatment, cell viability was measured using CellTiter-Glo (Promega, G9291) according to manufacturer’s instructions. Cell viability was normalized to a day 0 measurement and then to DMSO to calculate the inhibitory response. SynergyFinder (24) was used to calculate the synergy score using the HSA model. A calculated value of greater than 10 indicates synergy between the 2 drugs.

### Incucyte live-cell imaging.

Cells were pretreated with EZH2 inhibitor for 6 days and then seeded in triplicates in clear-bottom, black plates at 5,000 cells/well on day –1. On day 0, they were dosed in charcoal-stripped FBS with EZH2 and AKT inhibitors, in media containing NucLight Rapid Red Reagent (Sartorius, 4717, 1:1,000) to label nuclei and Caspase-3/7 Green Detection Reagent (Invitrogen, C10423, 1:1,000) to label cells undergoing apoptosis. Plates were placed in the Incucyte machine (Sartorius), and images were taken in phase, green (300 ms acquisition time), and red (400 ms acquisition time) channels every 2 hours for 4 days. Incucyte software was used to calculate percentage of dying cells by calculating overlap of red-labeled nuclei and green-labeled apoptotic cells normalized to total number of red-labeled nuclei.

### Crystal violet.

Cells were pretreated with EZH2 inhibitor for 6 days, then seeded in triplicates in 12-well plates at 100,000 cells per well. On day 0, they were dosed in charcoal-stripped FBS with EZH2 and PI3K pathway inhibitors for 3 weeks. Media with drugs were refreshed every 3–4 days. Plates were washed 3 times with PBS, fixed in 2% formaldehyde (dilution from Formaldehyde-Fresh, Thermo Fisher Scientific, SF94-4) for 30 minutes, washed once with PBS, and stained with 0.02% crystal violet (Sigma-Aldrich, C6158) for 1 hour. After staining, cells were washed 3 times with water, air-dried overnight, and imaged using a standard scanner.

### In vivo C4.2B cell line xenograft.

All mouse work was conducted in compliance with the Institutional Animal Care and Use Committee at Brigham and Women’s Hospital. Subcutaneous xenografts were generated by injecting cells into castrated 7- to 10-week-old male mice. A total of 10^6^ C4.2B cells in 1:1 media/Matrigel were injected into the left and right flanks of NSG mice from Charles River Laboratory. When all tumors reached more than 200 mm^3^, mice were randomized between treatment groups to ensure average tumor sizes at the start of treatment. There were 4 treatment groups: vehicle, PI3K inhibitor GDC-0941, EZH2 inhibitor GSK-126, and combination treatment. Mice were pretreated with GSK-126 for 7 days, followed by addition of GDC-0941 treatment for 11–13 days (for a total of 18–20 days). GSK-126 was diluted in 20% Captisol, pH 4.5, and GDC-0941 was diluted in methylcellulose (0.5% v/v) and polysorbate 80 (0.2% v/v). GSK-126 was administered at 300 mg/kg dose, twice a week via i.p. injection. GDC-0941 was given at 150 mg/kg daily via oral gavage. Tumor size and mouse body weight were measured twice a week. Animals were euthanized upon completion of the experiment.

### In vivo PDXs.

All mouse work was conducted in compliance with the Institutional Animal Care and Use Committee at Brigham and Women’s Hospital. LuCaP35-CR and LuCaP70-CR (27) PDX tumor tissues were a gift from Beth Israel Deaconess Medical Center and Harvard Medical School, Boston, Massachusetts, USA. Homogenized tumor chunks in 1:1 media: Matrigel were injected subcutaneously using 18G needles into the right flanks of 7- to 10-week-old castrated male ICR SCID mice from Taconic Biosciences. Tumors measuring approximately 50–300 mm^3^ were randomized and enrolled into 1 of the 4 treatment groups: vehicle, AKT inhibitor ipatasertib, EZH2 inhibitor tazemetostat, and combination treatment. Mice were pretreated with vehicle or tazemetostat for 7 days, followed by vehicle, ipatasertib, tazemetostat, or combination treatment for an additional 14 days for LuCaP35-CR (total 21 days) and 21 days for LuCaP70-CR (total 28 days). A separate cohort of tumor-bearing mice were treated for 2–4 days with vehicle or the drug combination (after 7 days of pretreatment with vehicle or EZH2 inhibitor), and tumors were harvested for Western blot and immunohistochemistry. Ipatasertib was diluted in 0.5% methylcellulose, viscosity 4,000 cP, with 2% Tween-80 at pH 7.0. Tazemetostat was prepared in 0.5% methylcellulose containing 0.1% Tween-80. Ipatasertib was administered at 50 mg/kg dose, once a day via oral gavage. Tazemetostat was administered at 250 mg/kg dose, twice a day via oral gavage. Tumor size and mouse body weight were measured twice a week. Animals were euthanized upon completion of the experiment.

### Western blotting.

Protein lysates were collected from cells after 6–24 hours of treatment with DMSO, EZH2 inhibitor alone, PI3K/AKT/mTORC1 inhibitor alone, or combination treatment, and from in vivo tumor tissue 2 hours after final dose on day 2, 3, or 4 of vehicle or combination treatment with EZH2 and AKT inhibitors. Cells were washed once with PBS and lysed on the plate using a cell scraper in boiling 1% SDS lysis buffer (1% SDS, Invitrogen, 15553-035), 10 mM Tris-HCl pH 7.5 (Sigma-Aldrich, 77-86-1), and 100 mM NaCl (Sigma-Aldrich, S5586). Snap-frozen tumor tissue from in vivo experiments was homogenized using a Biomasher tube (Research Products International, 199623) before addition of 1% SDS lysis buffer. Protein lysates were sheared using a 20G needle 6–8 times, boiled again at 95°C for 5 minutes, and centrifuged at maximum speed for 2 minutes. Colon cancer protein samples were collected as previously described (35). Protein concentration was quantified using a commercially available BCA kit according to manufacturer’s instructions (Thermo Fisher Scientific, 23225), and samples were prepared with SDS sample buffer (Boston Bioproducts, BP-111R) for running gels. Prepared protein samples were run on SDS-PAGE gels (Bio-Rad, 4561096 and 4561036) and transferred onto Immobilon PVDF membranes (Sigma, IPVH00010). Membranes were blocked in 5% milk in TBS-Tween (TBST) for 1 hour and incubated with primary antibodies overnight at 4°C. Membranes were washed 3 times with TBST, incubated with HRP-conjugated secondary antibody for 1 hour, and washed in TBST again. HRP signal (Thermo Fisher Scientific, 32106 and 34095) was measured on autoradiographic film or on Bio-Rad Chemidoc machine. The following antibodies were used: p-AKT (Cell Signaling Technologies, 4060, 1:1,000), AKT (Cell Signaling Technologies, 9272, 1:1,000), p-PRAS40 (Cell Signaling Technologies, 2997, 1:1,000), PRAS40 (Cell Signaling Technologies, 2691, 1:1,000), p-S6 (Cell Signaling Technologies, 4858, 1:1,000), S6 (Cell Signaling Technologies, 2217, 1:2,000), p-4EBP1 (Cell Signaling Technologies, 9451, 1:800), 4EBP1 (Cell Signaling Technologies, 9452, 1:800), H3K27me3 (Cell Signaling Technologies, 9733, 1:1,000), H3 (Cell Signaling Technologies, 4499, 1:2,500), GAPDH (Cell Signaling Technologies, 2118, 1:5,000), HK2 (Cell Signaling Technologies, 2867, 1:800), GLUT1 (Cell Signaling Technologies, 12939, 1:800), MYC (Abcam, ab32072, 1:1,000), HIF-1A (Cell Signaling Technologies, 14179, 1:800), p-AMPK (Cell Signaling Technologies, 2535, 1:800), AMPK (Cell Signaling Technologies, 2532, 1:800), BMF (Cell Signaling Technologies, 50542, 1:800), EZH2 (BD Biosciences, 612666), and ATF3 (Cell Signaling Technologies, 18665, 1:1,000).

### Immunohistochemistry.

Tumors from in vivo experiments were collected 2 hours after final dose on day 2, 3, or 4 of vehicle or combination treatment with EZH2 and AKT inhibitors. Tumor tissue was fixed in Formaldehyde-Fresh (Thermo Fisher Scientific, SF94-4) for 24 hours, then stored in 70% ethanol. Immunohistochemistry was performed at the Brigham & Women’s Hospital Pathology Core. The following antibodies were used: Ki67 (Biocare, CRM325), cleaved caspase-3 (Cell Signaling Technologies, 9664), and MYC (Abcam, ab32072).

### Steady-state metabolomics.

Similarly to proliferation cell counting assay, cells were first pretreated with EZH2 inhibitor for 6 days and then seeded in triplicate in 6-well plates at 450,000 cells/well on day –1. On day 0, they were dosed in charcoal-stripped FBS with EZH2 and AKT inhibitors. After 6 hours of combined treatment, plates were washed with PBS and snap-frozen in liquid nitrogen. Cells from an additional 6-well plate were counted and used for normalization. Metabolites were extracted from the plates using a 2:2:1 (v/v/v) mixture of LC-MS–grade acetonitrile, methanol, and water on dry ice. Samples were vortexed for 15 minutes and centrifuged at 16,000*g* for 15 minutes at 4°C. The resulting supernatant was collected and injected into the mass spectrometer for LC-MS analysis. Metabolites were separated using a Vanquish U-HPLC system coupled to a Q Exactive HF-X hybrid quadrupole-Orbitrap mass spectrometer (Thermo Fisher Scientific) with a HESI source operating in negative ion mode. Analytes were separated using an iHILIC column (5 μM, 150 × 2.1 mm I.D., HILICON) connected to a Thermo Fisher Scientific SII UPLC system. For polar targeted metabolomics analysis, buffer A (water with 20 mM ammonium carbonate and 0.1% ammonium hydroxide, pH 9.3) and buffer B (acetonitrile) were used on the iHILIC LC column. The Vanquish U-HPLC was run at a flow rate of 0.150 mL/min, 0–23 min linear gradient from 95% B to 5% B; 23–25 min hold at 5% B, diverted to waste from 25–25.5 min gradient to 95% B at 0.20 mL/min, 25.5–32.5 min hold at 95% B, and finally 32.5–33 min 95% B at 0.15 mL/min. Data acquisition and quantification were conducted using TraceFinder v4.1 (Thermo Fisher Scientific). Data were normalized based on extraction volume, cell counts, and an internal spiked-in standard (D8-phenylalanine). MetaboAnalyst was used for statistical analysis, metabolic pathway mapping, and enrichment analysis (51). *P* value less than 0.05 was used for significance.

### Glucose uptake and lactate secretion measurements.

Cells were pretreated with EZH2 inhibitor for 6 days, then seeded in triplicates in 6-well plates at 300,000 cells/well on day –1. On day 0, cells were dosed in charcoal-stripped FBS with EZH2 and AKT inhibitors. An extra plate with only media was also used to control for evaporation. Media were collected at time of treatment on day 0 and again after 24 hours. Glucose and lactate were measured on a 2900 Biochemistry Analyzer (YSI Inc.). Glucose consumption and lactate excretion were calculated by subtracting final from initial glucose or lactate amount after accounting for changes in media volume due to evaporation. This was normalized to the area under the growth curve and plotted relative to DMSO vehicle treatment group.

### Seahorse assays.

The oxygen consumption rate of cells was measured after 6, 12, and 24 hours of combination treatment with EZH2 and AKT inhibitors using the Seahorse XF Pro Analyzer (Agilent) and an XF Cell Mito Stress Test Kit (Agilent, 103015-100) according to kit instructions. Cells were first pretreated for EZH2 inhibitor for 6 days and then seeded in Seahorse assay 96-well plates (Agilent, 103793-100) and clear-bottom, black plates for Incucyte at 5,000 cells/well on day –1. A total of 5–8 replicates were seeded per condition. On day 0, they were dosed in charcoal-stripped FBS with EZH2 and PI3K pathway inhibitors. The media for the Incucyte plate also contained NucLight Rapid Red Reagent (Sartorius, 4717, 1:1,000) to label nuclei. Incucyte plate was placed in the Incucyte machine, and images were taken in phase and red (400 ms acquisition time) channels every 2 hours for 2 days. Seahorse plates were normalized by cell counting using Incucyte software counts of red-stained nuclei. At 1 hour prior to running the Seahorse plates, media were replaced with appropriate Seahorse XF RPMI media containing compound of interest at 37°C and the absence of CO_2_. Three basal measurements were made at 3-minute intervals, followed by 3 measurements per treatment. For mitochondrial stress tests, the preincubation medium consisted of Seahorse XF RPMI medium (pH 7.4; Agilent 10376-100) supplemented with 2 mM glutamine (Thermo Fisher Scientific 25030081), 1 mM pyruvate (Agilent 103578-100), and 10 mM glucose (Agilent 103577-100). Treatments comprised 1 μM oligomycin, 1.5 μM FCCP, and 100 nM rotenone plus 1 μM antimycin A (Agilent 103016-100). For glycolysis stress tests, the preincubation medium consisted of Seahorse XF RPMI medium (pH 7.4) supplemented with 2 mM glutamine. Treatments comprised 10 mM glucose (Agilent 103577-100), 1 μM oligomycin, and 50 mM 2-DG (Agilent 103020-100).

### ATP quantification.

Cells were first pretreated with EZH2 inhibitor for 6 days and then seeded in white, flat-bottom, 96-well plates for CellTiter-Glo and clear-bottom, black, 96-well plates for Incucyte at 7,000 cells/well on day –1. A total of 6 replicates were seeded per condition. On day 0, they were dosed in charcoal-stripped FBS with EZH2 and AKT inhibitors. The media for the Incucyte plate also contained NucLight Rapid Red Reagent (Sartorius, 4717, 1:1,000) to label nuclei. Incucyte plates was placed in the Incucyte machine, and images were taken in phase and red (400 ms acquisition time) channels every 2 hours for 2 days. Incucyte software counts of red-stained nuclei were used to determine cell counts. After 24 hours of combined treatment, CellTiter-Glo (Promega, G9291) assay was conducted according to manufacturer’s instructions to determine relative ATP levels by luminescence. Relative ATP levels were normalized by cell counts.

### BMF ectopic expression.

pBabe neo BMF (Addgene plasmid 17239) was a gift from Joan Brugge (Harvard Medical School, Boston, Massachusetts, USA) (52). Negative control pBabe neo empty plasmid (Addgene plasmid 1767) was a gift from Hartmut Land and Jay Morgenstern (Imperial Cancer Research Fund, Lincoln’s Inn Fields, London, United Kingdom) (53). Plasmids were introduced into C4.2B cells using retroviral transduction. Retrovirus was produced in 293T cells using X-tremeGENE 9 DNA Transfection Reagent (Roche, 6365787001). Virus was harvested after 48 hours and used to infect cells twice for 24 hours each at 1:2 dilution with 6 μg/mL polybrene (Sigma-Aldrich, H9268). Cells were given 24 hours to recover from the infection and then selected in 0.4 mg/mL neomycin (Thermo Fisher Scientific, 10131-035) for 6–7 days.

### RNA-seq.

Cells were pretreated with EZH2 inhibitor for 6 days, then seeded in triplicates in 6 cm plates at 250,000 cells/well on day –1. On day 0, they were dosed in charcoal-stripped FBS with EZH2 and PI3K pathway inhibitors. After 24 hours of combined treatment, RNA was isolated using RNeasy Plus Mini kit (QIAGEN, 74136). RNA was sequenced using the Illumina NextSeq 500 system through Dana-Farber Cancer Institute Molecular Biology Core Facility or Novogene. Raw data were aligned to hg38 and turned into counts using STAR and HTSeq (54, 55). DESeq2 was used to normalize counts and perform differential expression analysis between treatment arms (29). Adjusted *P* value < 0.05 was used for significance. RNA-seq in colorectal cancer was conducted as previously described (35). ssGSEA and GSEA (56, 57) were performed using the DESeq2-normalized counts on GenePattern (https://www.genepattern.org). Hallmark, KEGG, and PID gene signatures were obtained from Molecular Signatures Database (https://www.gsea-msigdb.org/gsea/msigdb) (28). Significance was determined by FDR < 0.25.

### CUT&TAG.

CUT&TAG was performed as previously described (58). Briefly, cells were first pretreated with EZH2 inhibitor for 6 days and seeded in duplicate in 15 cm plates at 2,310,000 cells per plate. On day 0, they were dosed in charcoal-stripped FBS with EZH2 and AKT inhibitors. After 24 hours of cotreatment, cells were collected in PBS using a cell scraper, then pelleted, and nuclei were extracted using nuclei extraction buffer (20 mM HEPES-KOH pH 7.9, 10 mM KCl, 0.1% Triton X-100, 20% glycerol, 0.5 mM spermidine, 1× Roche Complete Mini EDTA-free protease inhibitor). Concanavalin A magnetic beads (Bangs Laboratories, BP531) were activated using bead activation buffer (20 mM HEPES pH 7.9, 10 mM KCl, 1 mM CaCl_2_, 1 mM MnCl_2_). Extracted nuclei and activated concanavalin A beads were incubated together overnight (4°C) with 0.5 μg of IgG (EpiCypher, 13-0042) or H3K27me3 (Cell Signaling Technology, 9733S) antibody on a shaker. A magnet was used to wash off primary antibody, and samples were incubated in secondary antibody in Digitonin 150 buffer (20 mM HEPES pH 7.5, 150 mM NaCl, 0.5 mM spermidine, 1× Roche Complete Mini EDTA-free Protease Inhibitor, 0.01% Digitonin) at 4°C for 1 hour. Samples were washed twice with Digitonin 150 buffer and incubated in Digitonin 300 buffer (20 mM HEPES pH 7.5, 300 mM NaCl, 0.5 mM spermidine, 1× Roche Complete Mini EDTA-free Protease Inhibitor, 0.01% Digitonin) containing pA-Tn5 (1:50 dilution) at 4°C for 1 hour. Samples were washed in Digitonin 300 buffer and incubated in tagmentation buffer (Digitonin 300 Buffer, 10 mM MgCl_2_) at 37°C for 1 hour to activate Tn5. Samples were then incubated in 5 μL of SDS release buffer (10 mM TAPS with 0.1% SDS) at 58°C for 1 hour to release Tn5. SDS quench buffer (0.67% Triton X-100) was added, and then samples were prepared for PCR using 2 μL of barcoded i5 primer (10 μM), 2 μL of barcoded i7 primer (10 μM), and 25 μL of NEBNext PCR mix. PCR amplification was performed in a thermocycler with 12 PCR cycles. CUT&TAG libraries were cleaned with SPRIselect beads (Beckman Coulter, B23319) and sequenced on Illumina NextSeq 2000 sequencer using paired-end sequencing. FASTQ files were aligned to hg38 reference genome using Bowtie2 with setting “—very-sensitive –no-mixed –no-discordant –phred33 -I 10 -X 700” (59). Spike-in normalization factor was calculated by using total number of mapped reads to *E*. *coli* genome. Samtools was used to convert resulting.sam files into.bam files (60), and Deeptools was used to generate BigWig files with “scale-factor” option of bamCoverage (61). IGV was used to make genome tracks, and peaks were called using MACS2 callpeak function (62, 63). DESeq2 and DiffBind R package was used to perform differential binding analysis between conditions. Significance was determined by FDR < 0.1 (64).

### BioRender.com.

BioRender.com was used to create the metabolism map presented in Figure 3D (BioRender.com/oo0ug18; agreement OL29K7TGOV), the model shown in Figure 5L (BioRender.com/wqg7vh5; agreement HT29KHY6AV), and the graphical abstract (BioRender.com/wqg7vh5; agreement CH29KHZ38X).

### Statistics.

For most in vitro quantitative experiments, error bars represent ± SD, and *P* values were determined using 1-way ANOVA and Tukey’s post hoc test to allow for multiple comparisons. For Seahorse assays and in vivo studies, error bars represent ± SEM. For RNA-sequencing data, DESeq2 adjusted *P* values were used. A *P* value less than 0.05 was considered significant, except for GSEA, where FDR < 0.25 was used for significance. Most quantitative data were graphed and analyzed using GraphPad Prism or R. MetaboAnalyst was used to analyze metabolomics data (51). SynergyFinder was used to calculate the synergy score using the HSA model (24). A calculated value of greater than 10 indicates synergy between the 2 drugs.

### Study approval.

All mouse studies were conducted in compliance with and with the approval of the Institutional Animal Care and Use Committee at Brigham and Women’s Hospital (Protocol 2016N000467). No human subject research was performed.

### Data availability.

All RNA-sequencing and CUT&TAG data are uploaded to NCBI GEO. RNA-seq data of C4.2B cells treated with AKT inhibitor ipatasertib and EZH2 inhibitor tazemetostat are deposited under GSE297894, and RNA-seq data of C4.2B cells treated with PI3K inhibitor GDC-0941 and EZH2 inhibitor GSK-126 are deposited under GSE297897. H3K27me3 CUT&TAG data in C4.2B cells treated with ipatasertib and tazemetostat are deposited under GSE297899.Values for all data points in graphs are reported in the Supporting Data Values file.

## Author contributions

Conceptualization, investigation, and design were contributed by RS, ME, and KC. Resources and methodology were contributed by RS, ME, AES, VTMN, DRS, SY, SS, AG, AY, AI, KK, SJ, EM, SH, FX, KH, MGVH, SPB, MCH, and KC. Data curation was performed by RS, ME, AES, VTMN, DRS, SY, SS, AG, AY, AI, KK, SJ, EM, SH, KH, MGVH, SPB, MCH, and KC. Formal analysis was performed by RS, AES, VTMN, DRS, and SY. Supervision was done by KH, MGVH, SPB, MCH, and KC. Writing of the original draft and review of the manuscript were performed by RS and KC. LuCaP35-CR and LuCaP70-CR (27) PDX tumor tissues were a gift from FX and SPB.

## Conflict of interest

KC is a member of the scientific advisory board of Erasca and has served as an advisor for Merck. MCH serves on the scientific advisory boards of MitoQ, Alixia Therapeutics, Minovia, *Cell Metabolism*, and *Molecular Cell* and is a founder of and receives research funding from Refuel Bio. KH is a founder of Dania Therapeutics and is a member of the scientific advisory boards of Hannibal Innovation, Inthera Bioscience AG, and MetaboMed Inc. MGVH serves on the scientific advisory board for Agios Pharmaceuticals, iTeos Therapeutics, Sage Therapeutics, Lime Therapeutics, Faeth Therapeutics, Pretzel Therapeutics, Droia Ventures, MPM Capital, and Auron Therapeutics.

## Funding support

- Cancer Research UK Grand Challenge SPECIFICANCER team funding to KC and KH.
- Harvard University Landry Cancer Biology Research Fellowship to RS.
- Harvard Medical School Fujifilm Fellowship to RS.

## Supplementary Material

Supplemental data

Unedited blot and gel images

Supporting data values

## Version 1 06/09/2026

In-Press Preview

## Version 2 08/17/2026

Electronic publication
