# Supplementary material for: Epigenetic and oncogenic inhibitors converge to drive a metabolic catastrophe in castration-resistant prostate cancer
Source: J Clin Invest. 2026 Jun 9;136(16):e203835. doi: 10.1172/JCI203835 (PMC13476140; doi:10.1172/JCI203835)
Supplement: Supplementary file 1 [file jci-136-203835-s026.pdf]

Supplemental Figure 1

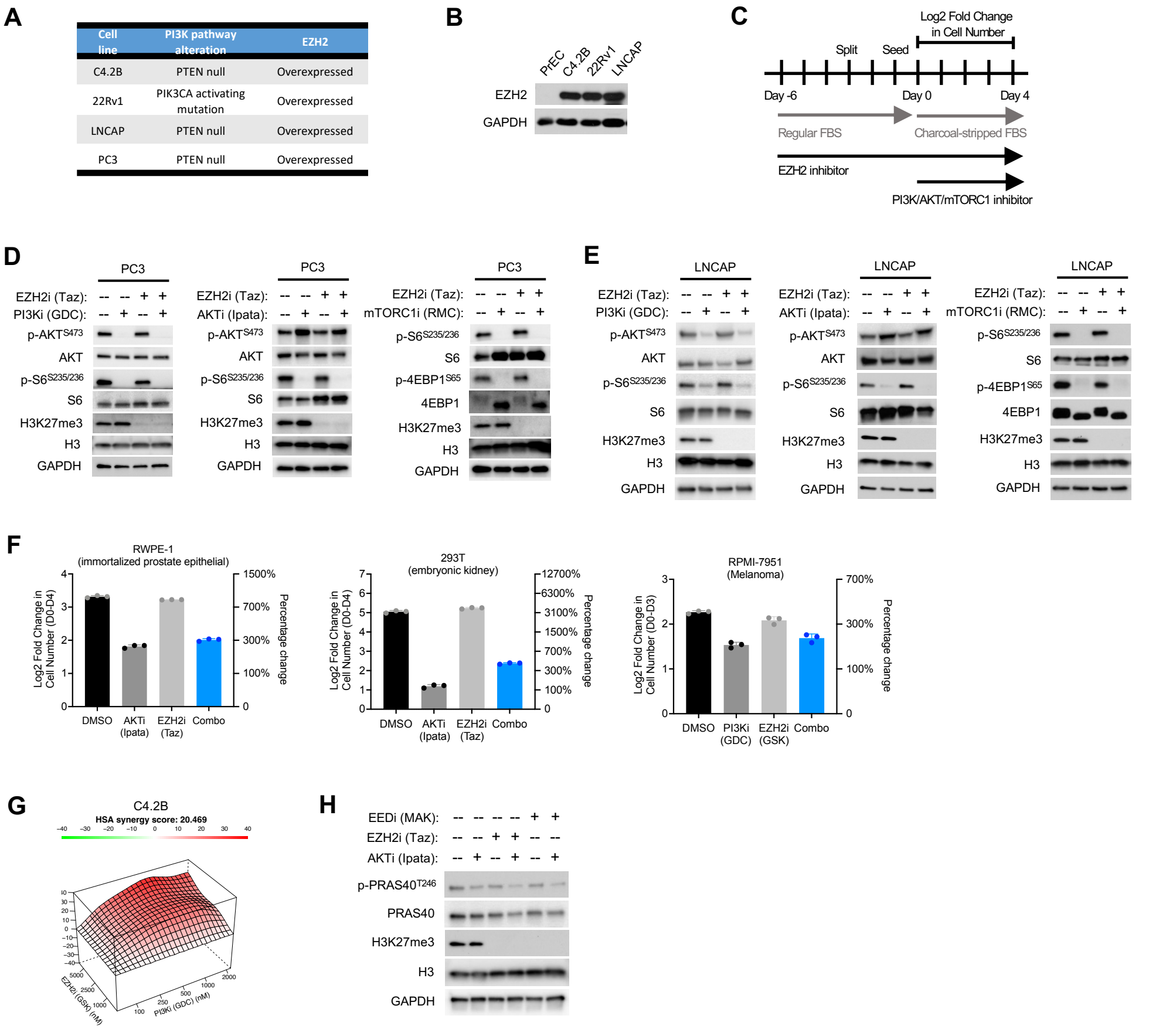

Supplemental Figure 1

EZH2 and PI3K/AKT/mTORC1 inhibitors selectively kill castration-resistant prostate cancer in vitro, related to figure 1

(A) Table summarizing PI3K signaling pathway activating alteration and EZH2 expression in cell lines used in figure 1. **(B)** Immunoblot showing high EZH2 expression in advanced prostate cancer C4.2B, 22Rv1, and LNCaP cell lines. GAPDH is used as a loading control. **(C)** Schematic of timeline for treatment with EZH2 and PI3K/AKT/mTORC1 inhibitors for cell proliferation assays. **(D-E)** Immunoblots confirming PI3K signaling target inhibition (p-AKT, p-S6, p-4EBP1) and EZH2 target inhibition (H3K27me3) with relevant loading controls after 9 hours of combination treatment in PC3 **(D)** and LNCaP **(E)**. **(F)** Graph depicting Log_2 fold change of cell number over 4 days of combination treatment with EZH2 inhibitor and PI3K/AKT inhibitor in RWPE-1 immortalized prostate epithelial, 293T human embryonic kidney, and RPMI7951 melanoma cell lines. n = 3. Error bars represent ± S.D. **(G)** Synergy plot between EZH2 inhibitor GSK-126 and PI3K inhibitor GDC-0941 using the HSA model of synergy in C4.2B cells. Score > 10 indicates synergy. n = 2. **(H)** Immunoblot depicting PI3K signaling pathway target inhibition and PRC2 complex target inhibition with relevant loading controls after 9 hours of combination treatment in C4.2B cells. EZH2 inhibitors: GSK-126 (GSK), tazemetostat (Taz). PI3K pathway inhibitors: PI3K inhibitor GDC-0941 (GDC), AKT inhibitor Ipatasertib (Ipata), mTORC1 inhibitor RMC-5552 (RMC).

Supplemental Figure 2

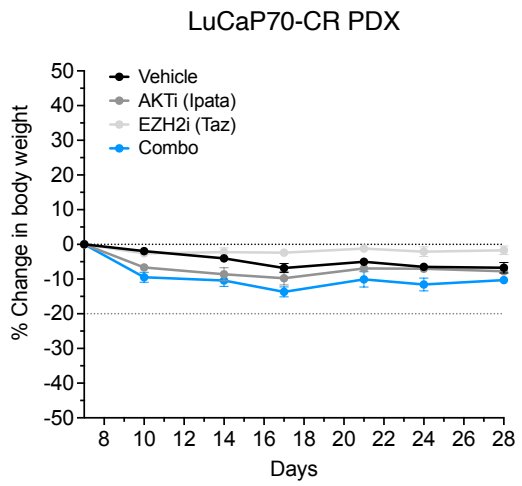

Supplemental Figure 2

Combination treatment with EZH2 and AKT inhibitors has minimal effect on body weight of mice, related to figure 2

Graph showing minimal percentage change in body weight compared to start of combination treatment with EZH2 inhibitor tazemetostat and AKT inhibitor ipatasertib in LuCaP70-CR PDX. n = 5 for vehicle, n = 6 for AKTi and EZH2i, n = 7 for Combo.

Supplemental Figure 3

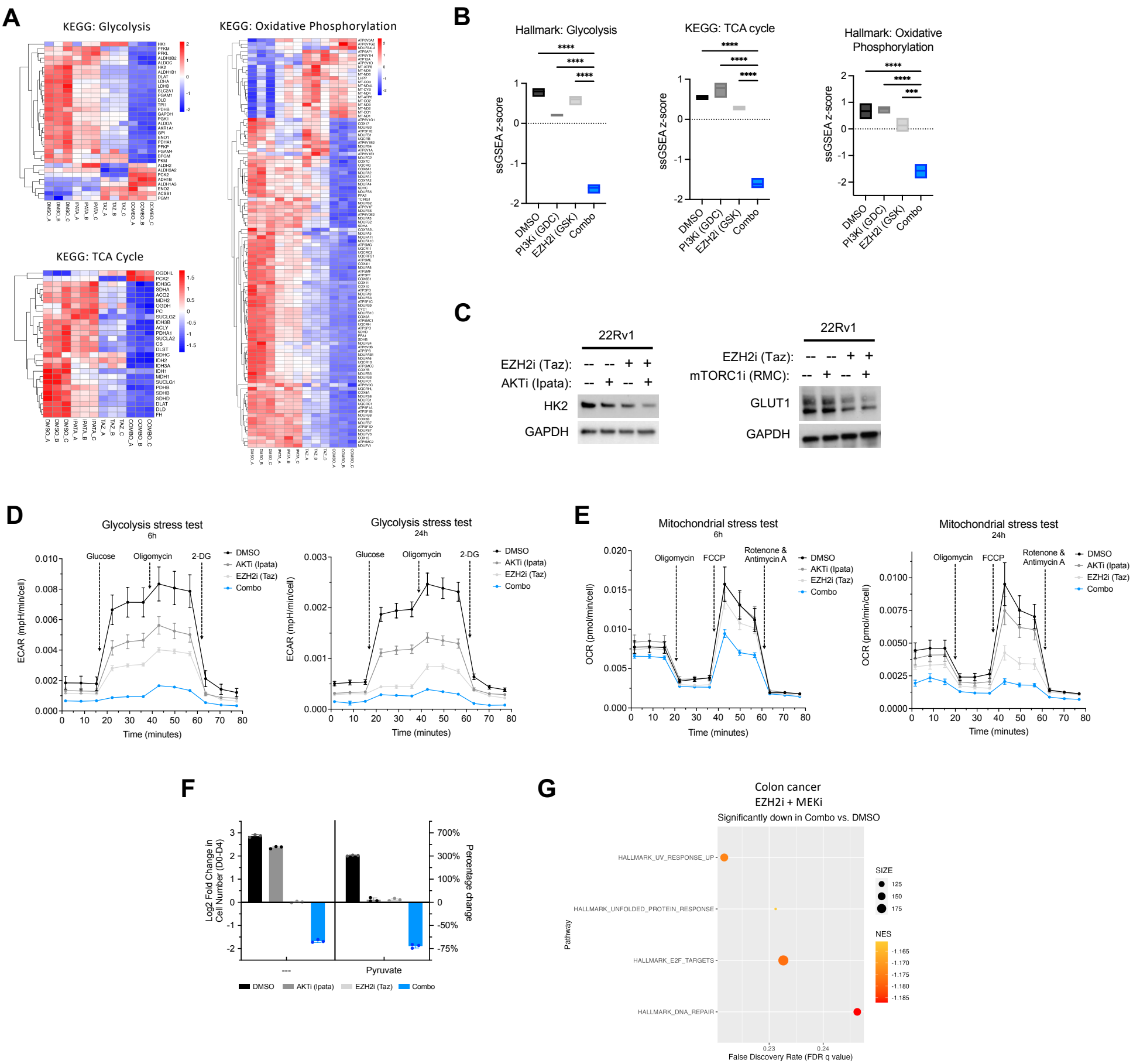

Supplemental Figure 3

EZH2 and PI3K pathway inhibitors suppress both glycolysis and oxidative phosphorylation in CRPC, related to figure 3

(A) Heatmaps of KEGG glycolysis, TCA cycle, and oxidative phosphorylation gene signatures in C4.2B cells treated with EZH2 inhibitor tazemetostat and AKT inhibitor ipatasertib. Includes all genes that significantly changed between combination treatment and DMSO. n = 3. **(B)** RNA-seq ssGSEA analysis in C4.2B highlights cooperative suppression of glycolysis, TCA cycle, and oxidative phosphorylation by EZH2 inhibitor GSK-126 and PI3K inhibitor GDC-0941. n = 3. Error bars represent ± S.D and p-values were determined using one-way ANOVA and Tukey's test. * $p<0.05$; ** $p<0.01$; *** $p<0.001$; **** $p<0.0001$. **(C)** Immunoblot showing decrease in HK2 and GLUT1 with EZH2i tazemetostat and AKTi ipatasertib or mTORC1i RMC-5552 in 22Rv1 cells after 6-9 hours. Note: HK2 blot is from the same experiment and has the same GAPDH loading control as Figure 1D. **(D-E)** Graphs showing Seahorse glycolysis stress test **(D)** and mitochondrial stress test **(E)** normalized to cell counts in C4.2B treated with EZH2 and AKT inhibitors together for 6 hours and 24 hours. n = 7 for DMSO and AKTi, n = 8 for EZH2i and Combo. Error bars represent ± S.E.M. **(F)** Graph showing $\log_2$ fold change of cell number after 4 days of co-treatment with EZH2 and AKT inhibitors in C4.2B cells cultured in media with or without sodium pyruvate. n = 3. Error bars represent ± S.D. **(G)** RNA-seq GSEA analysis depicting significantly downregulated Hallmark gene sets in colon cancer LOVO cells treated for 24 hours with combination of EZH2 inhibitor tazemetostat and MEK inhibitor trametinib compared to DMSO. Significance determined by FDR < 0.25. n = 3.

Supplemental Figure 4

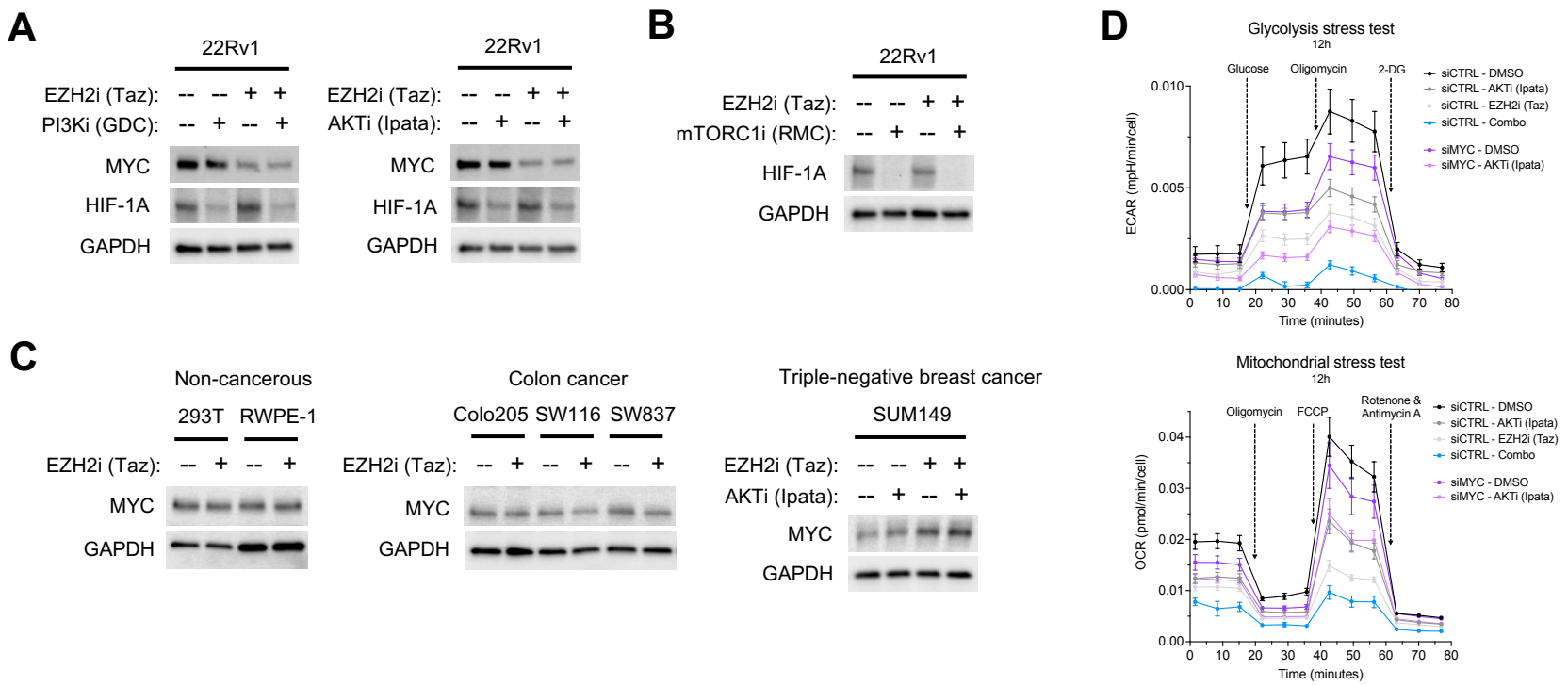

Supplemental Figure 4

EZH2 and PI3K pathway inhibitors decrease MYC and HIF-1A in CRPC but not in other cells, related to figure 4

(A) Immunoblot showing decrease in MYC and HIF-1A with EZH2 inhibitor tazemetostat and PI3K/AKT inhibitors (GDC-0941/ipatasertib) in 22Rv1 cells after 9 hours of co-treatment. **(B)** Immunoblot highlighting decrease in HIF-1A in 22Rv1 cells treated with mTORC1 inhibitor RMC-5552, with or without EZH2 inhibitor tazemetostat. Note: these blots **(A-B)** are from the same experiments and have the same GAPDH loading controls as Figure 1D. **(C)** Immunoblot showing effect of EZH2 inhibition on MYC protein levels in non-cancerous (293T, RWPE-1), colon cancer (Colo205, SW116, SW837), and triple-negative breast cancer (SUM149) cells. **(D)** Graphs showing Seahorse glycolysis stress test and mitochondrial stress test normalized to cell counts in C4.2B cells treated with EZH2 and AKT inhibitors together for 12 hours, with or without MYC knockdown via siRNA. n = 5. Error bars represent ± S.E.M.

Supplemental Figure 5

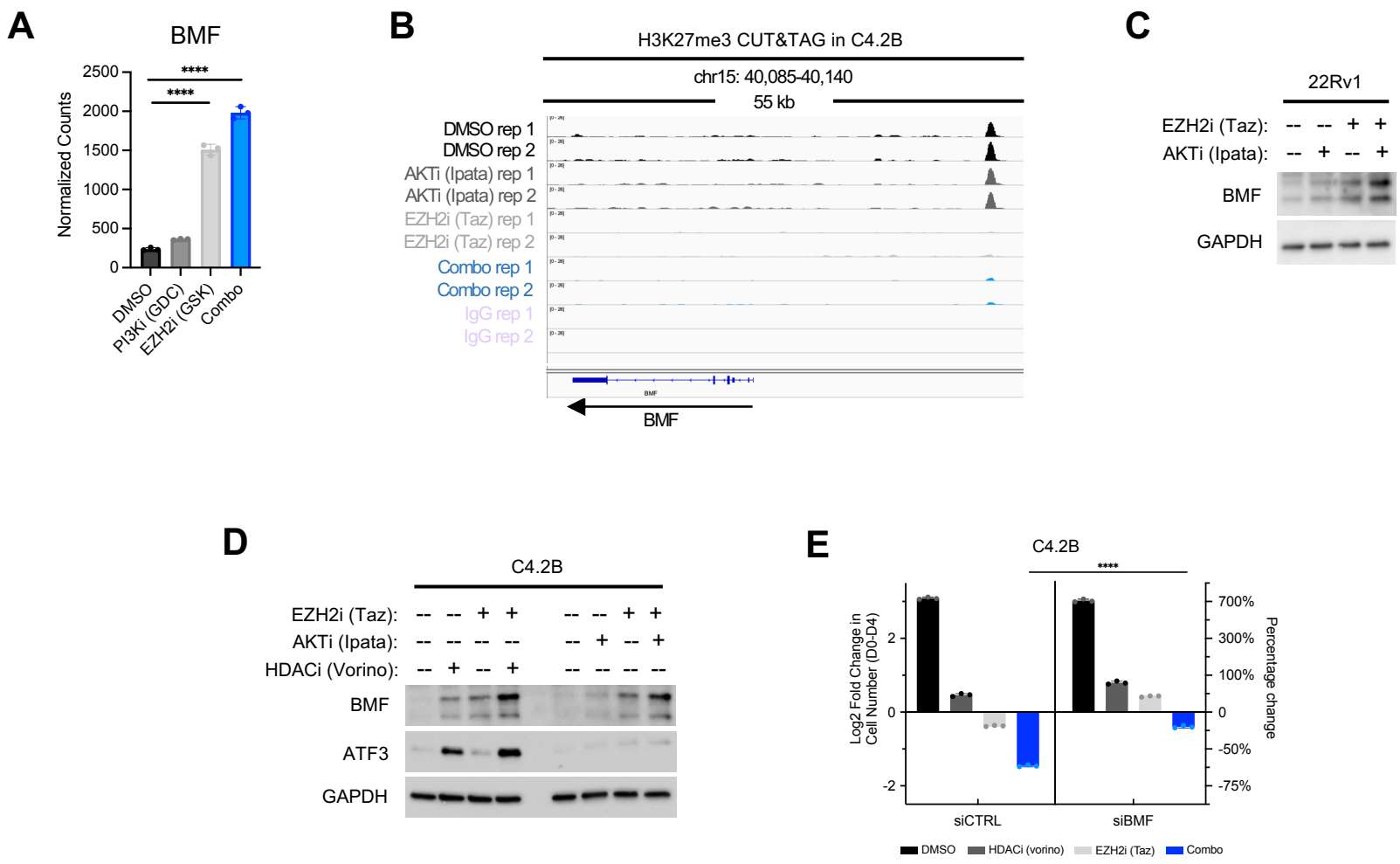

Supplemental Figure 5

EZH2 inhibition concomitantly derepresses BMF, a sensor of cellular stress that drives apoptosis, related to figure 5

(A) Normalized RNA-seq counts of BMF in response to EZH2 inhibitor GSK-126 and/or PI3K inhibitor GDC-0941 in C4.2B cells. Significance determined by DESeq2 adjusted p-value. n = 3. **(B)** CUT&TAG reveals loss of H3K27me3 mark in the region upstream of BMF gene locus in EZH2-inhibitor tazemetostat treated C4.2B cells, with or without AKT inhibitor ipatasertib. n = 2. **(C)** Immunoblot depicting induction of BMF with EZH2 inhibitor, with or without AKT inhibitor, after 24 hours in 22Rv1 cells. GAPDH is used as a loading control. **(D)** Immunoblot comparing induction of BMF and ATF3 with EZH2 inhibitor tazemetostat, with or without AKT inhibitor ipatasertib or HDAC inhibitor vorinostat after 9 hours in C4.2B. **(E)** Graph showing Log_2 fold change of cell number after 4 days of co-treatment with EZH2 and HDAC inhibitors in C4.2B cells, with or without BMF knockdown via siRNA. n = 3. Error bars represent ± S.D. and p-values were determined using one-way ANOVA and Tukey's test. * p<0.05; ** p<0.01; *** p<0.001; **** p<0.0001.
