# Supplementary material for: Epigenetic and oncogenic inhibitors converge to drive a metabolic catastrophe in castration-resistant prostate cancer
Source: J Clin Invest. 2026 Jun 9;136(16):e203835. doi: 10.1172/JCI203835 (PMC13476140; doi:10.1172/JCI203835)
Supplement: Supplementary file 2 [file jci-136-203835-s027.pdf]

Raw unedited blots for Figure 1B

C4.2B

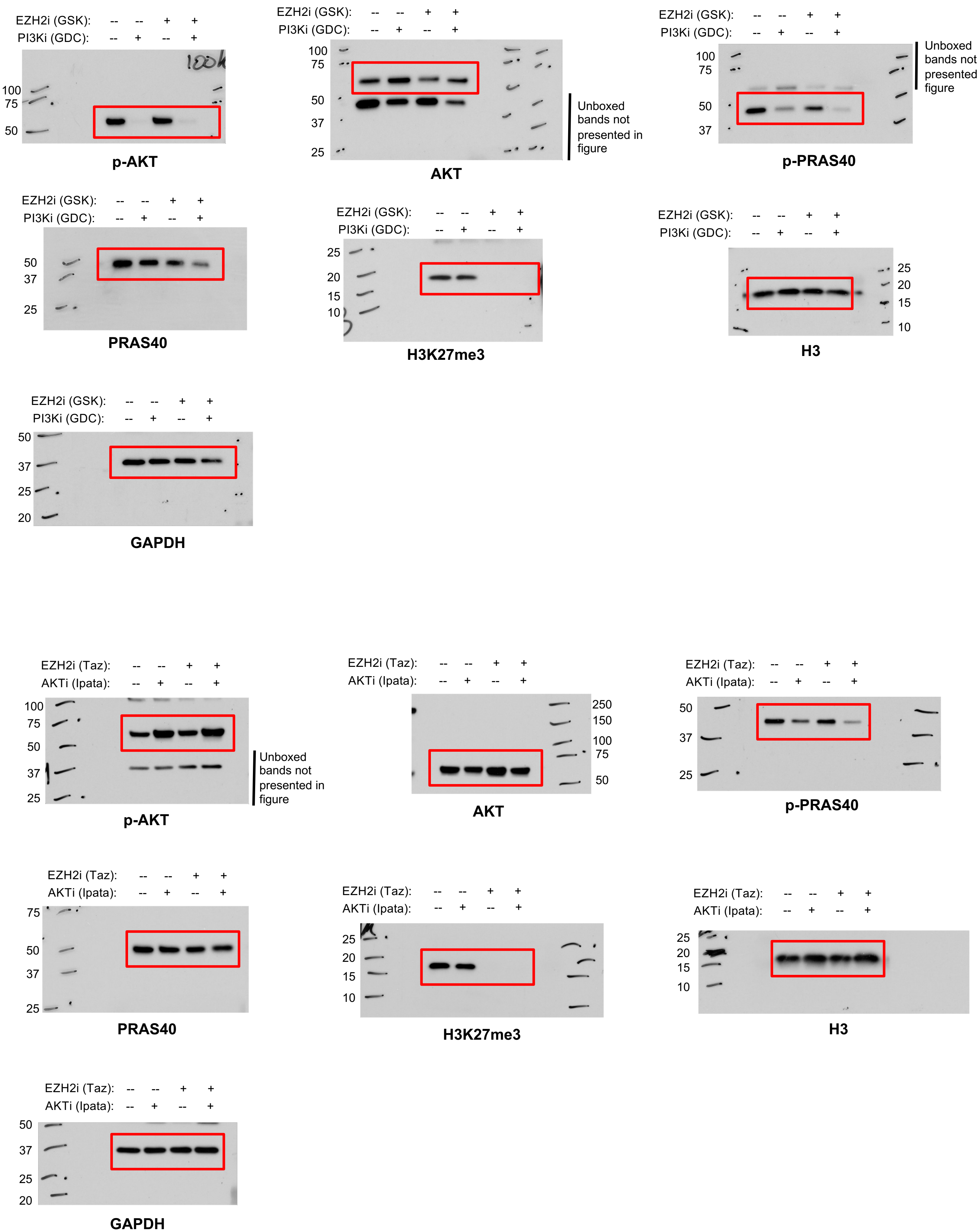

Raw unedited blots for Figure 1B

C4.2B

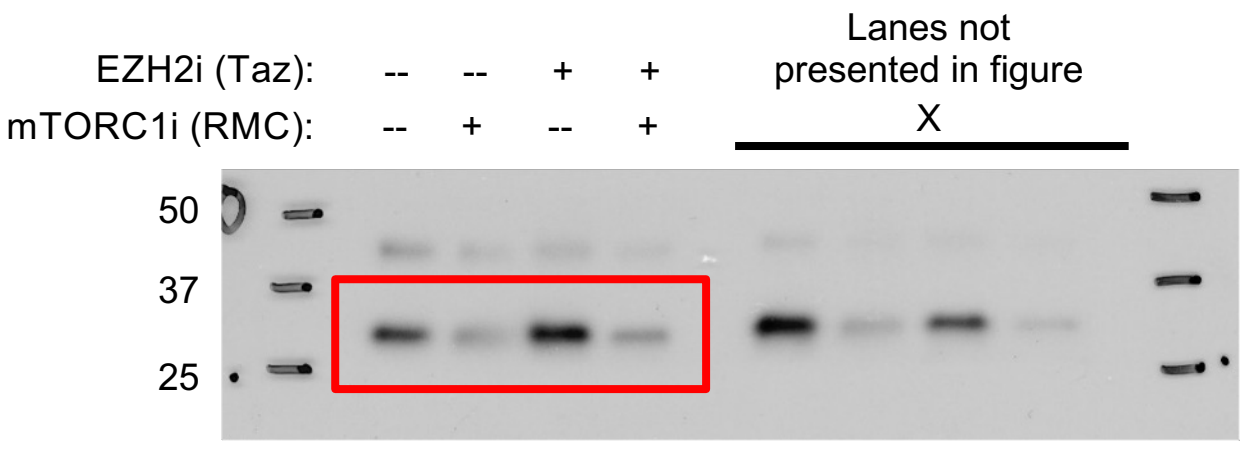

p-S6

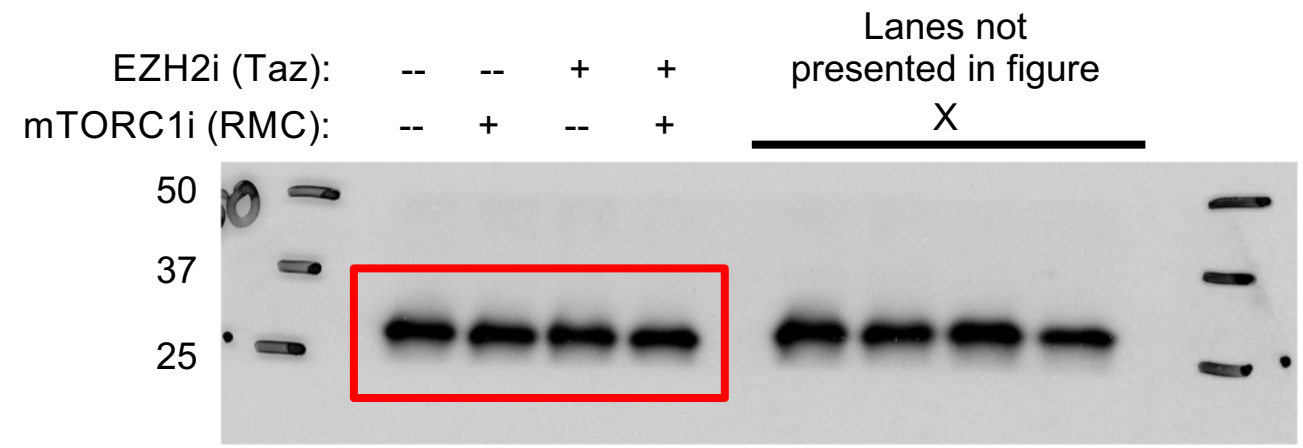

S6

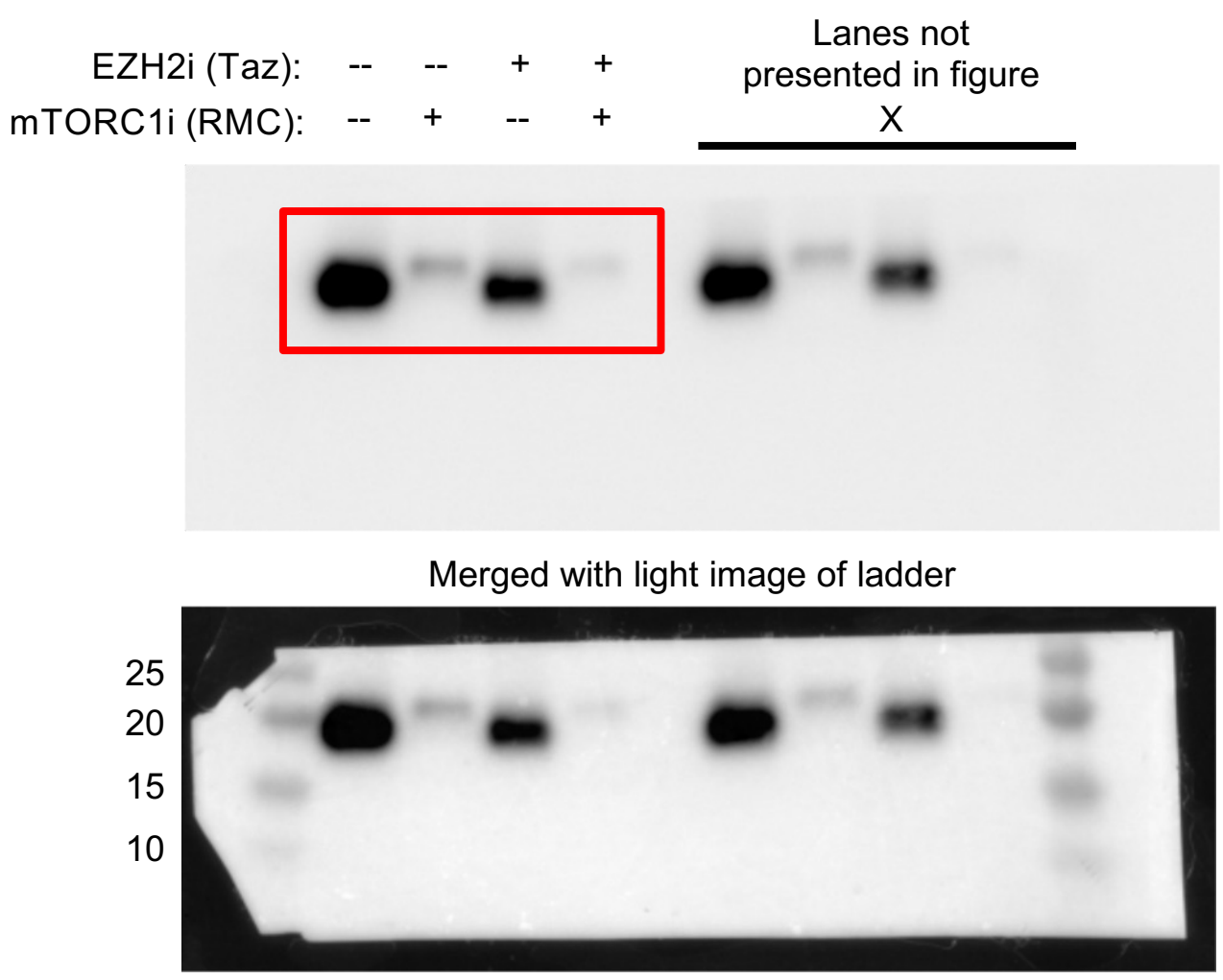

p-4EBP1

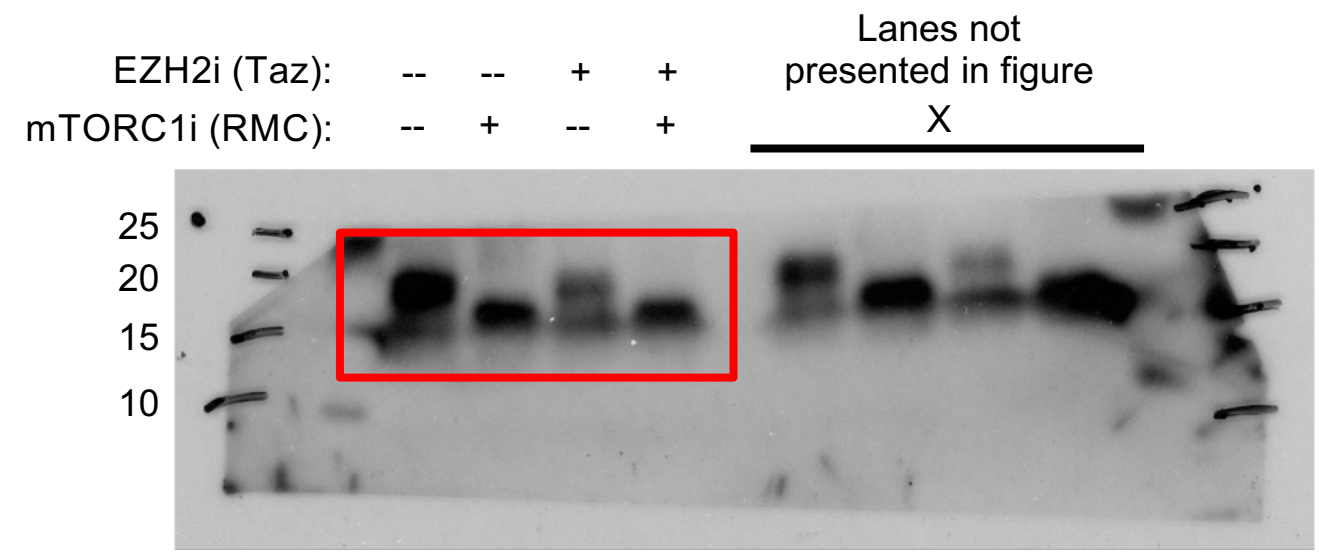

4EBP1

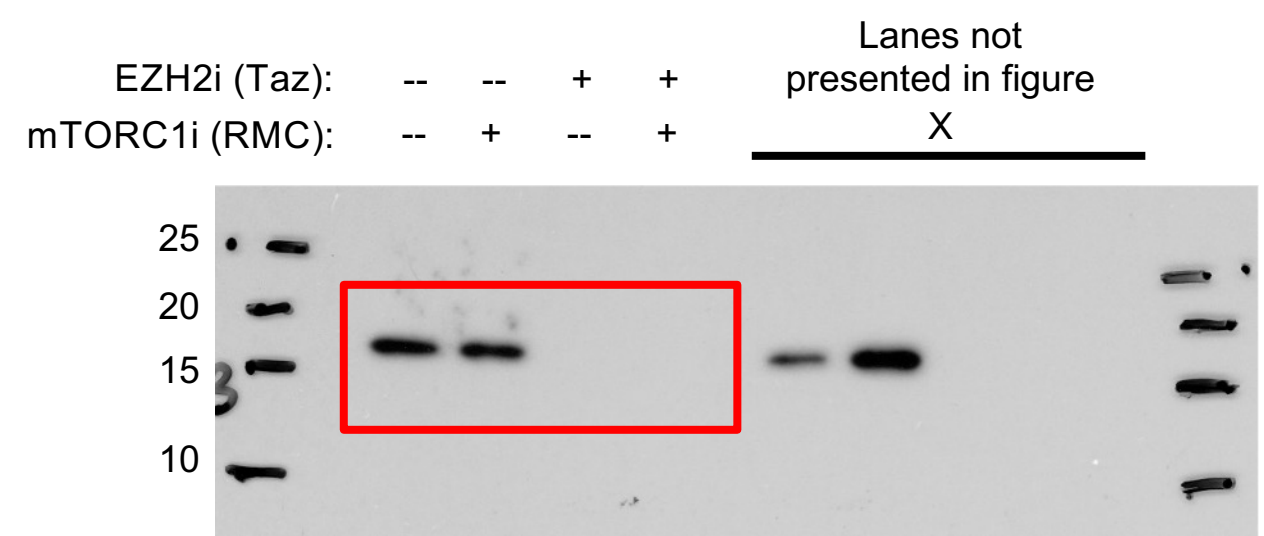

H3K27me3

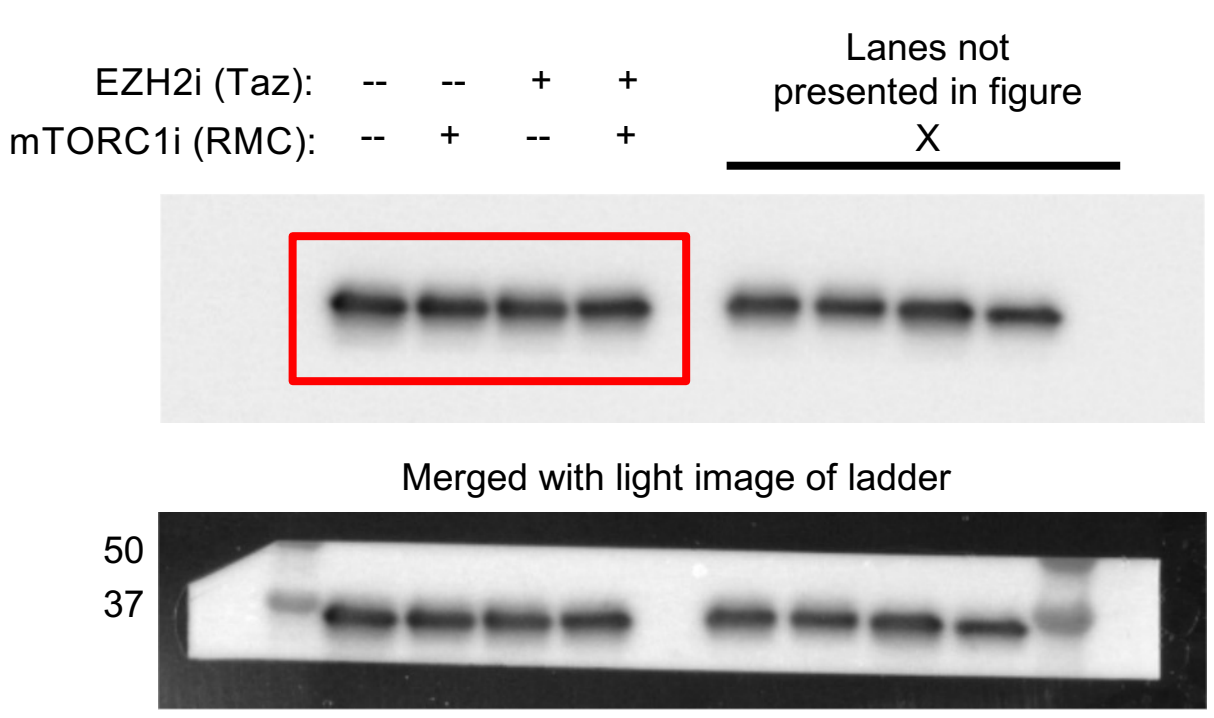

GAPDH

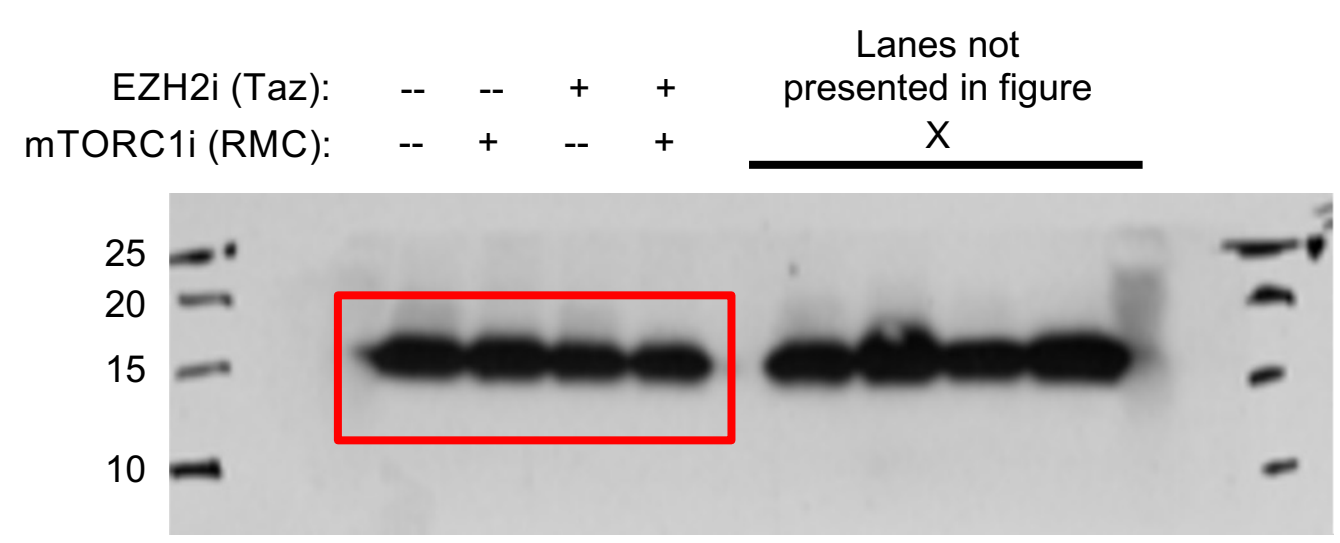

H3

Raw unedited blots for Figure 1D

22Rv1

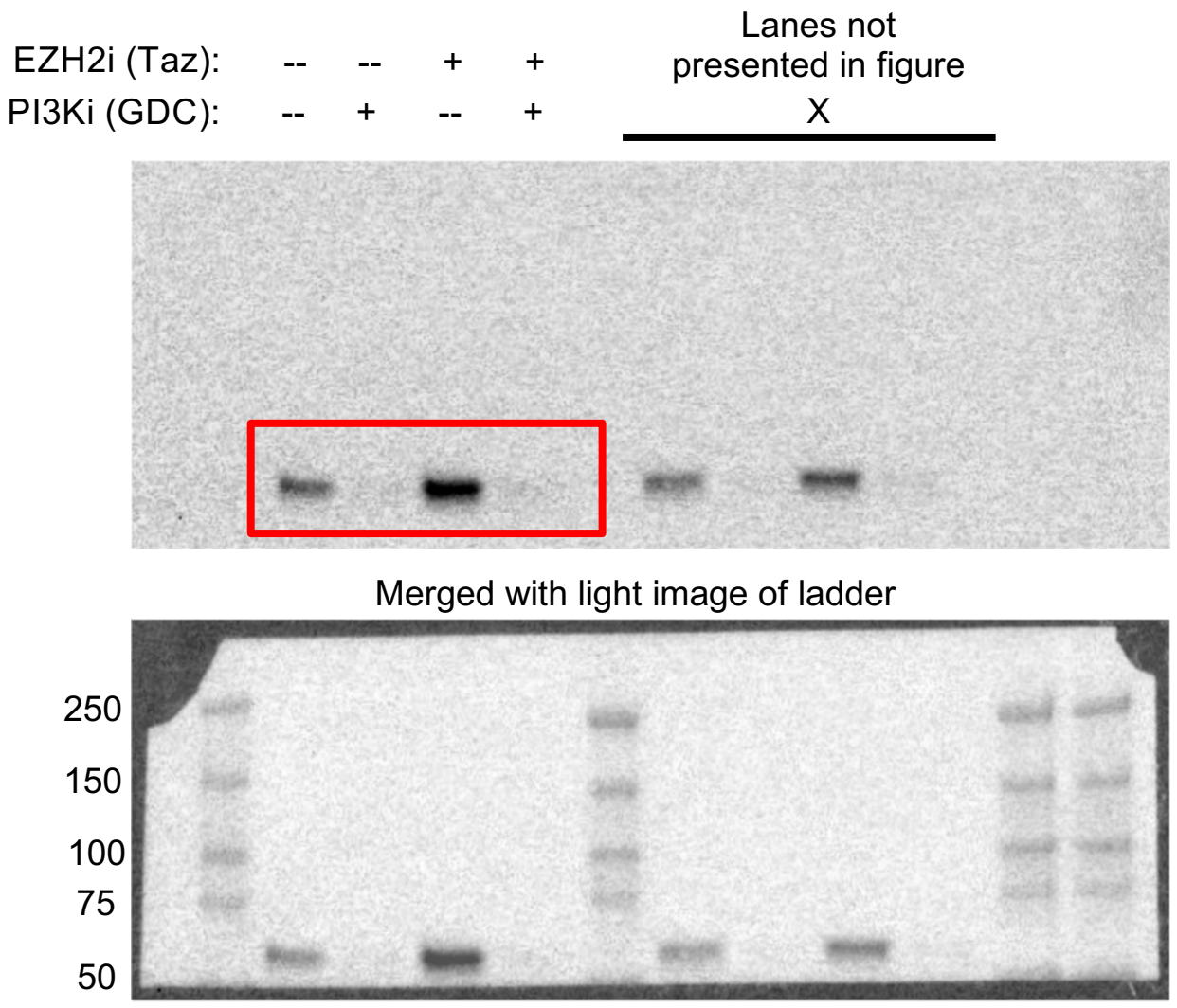

p-AKT

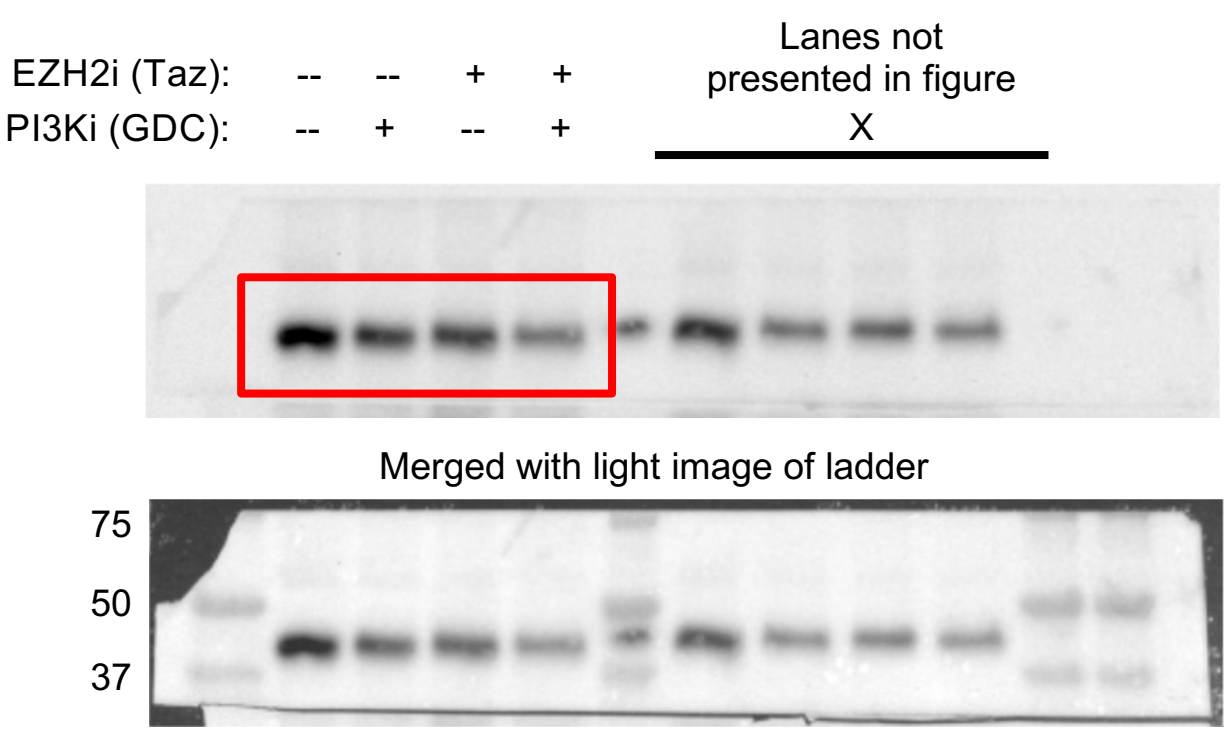

PRAS40

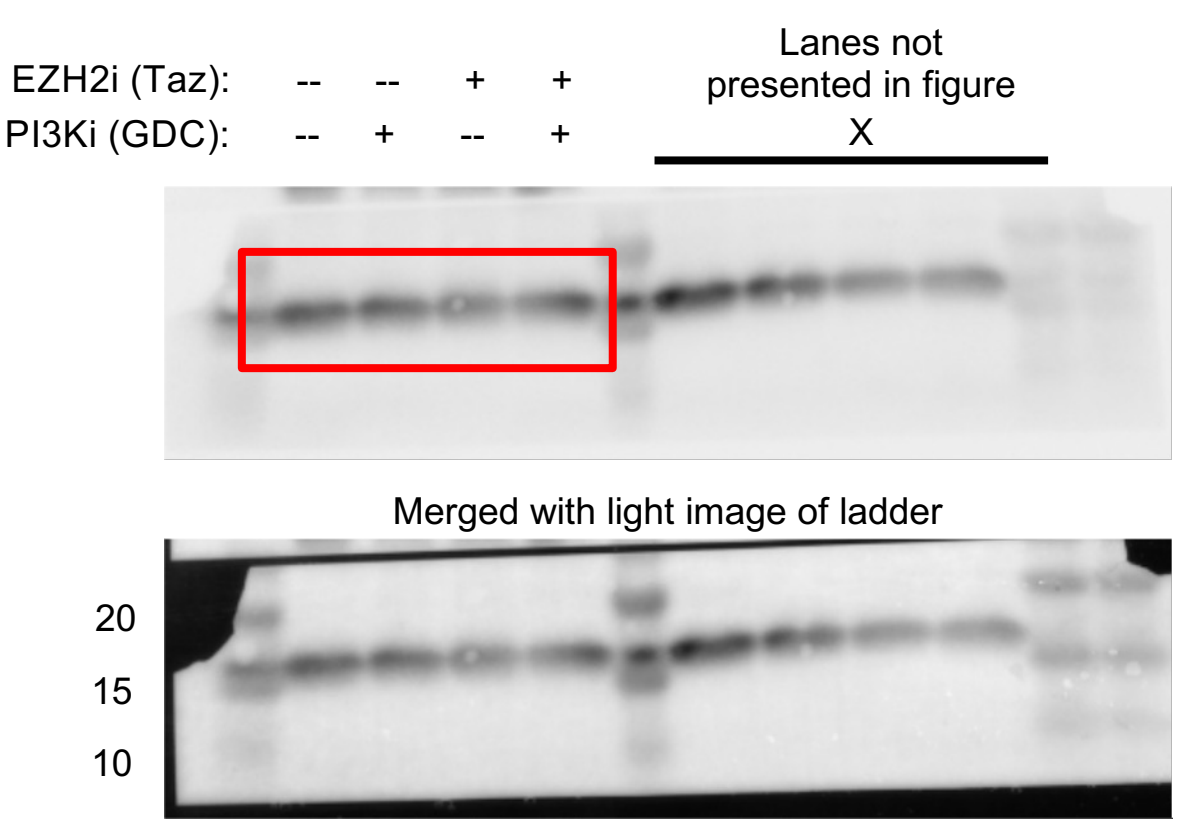

H3

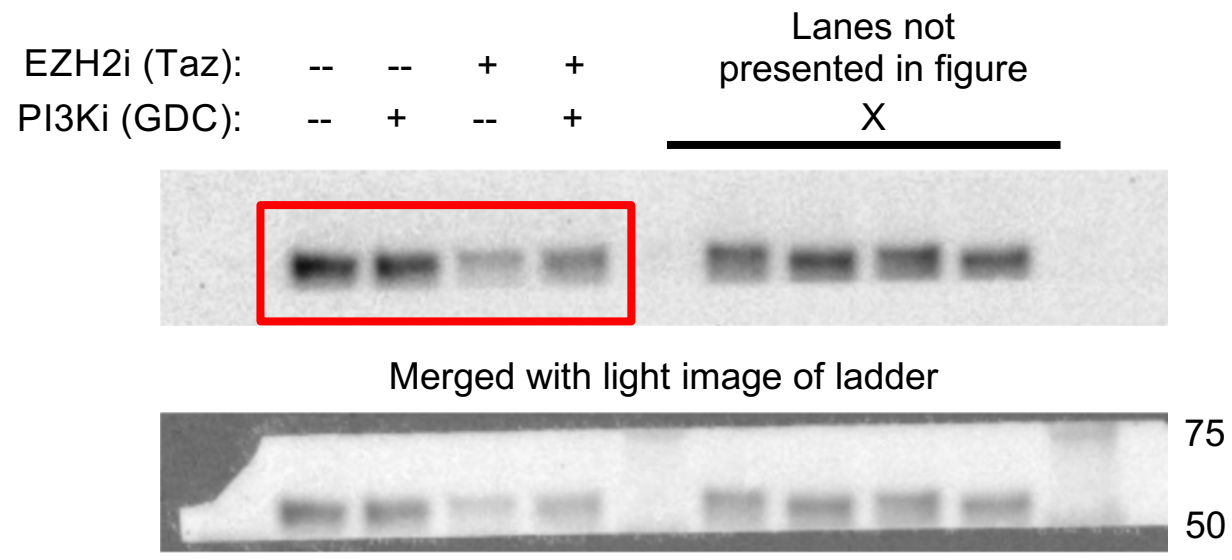

AKT

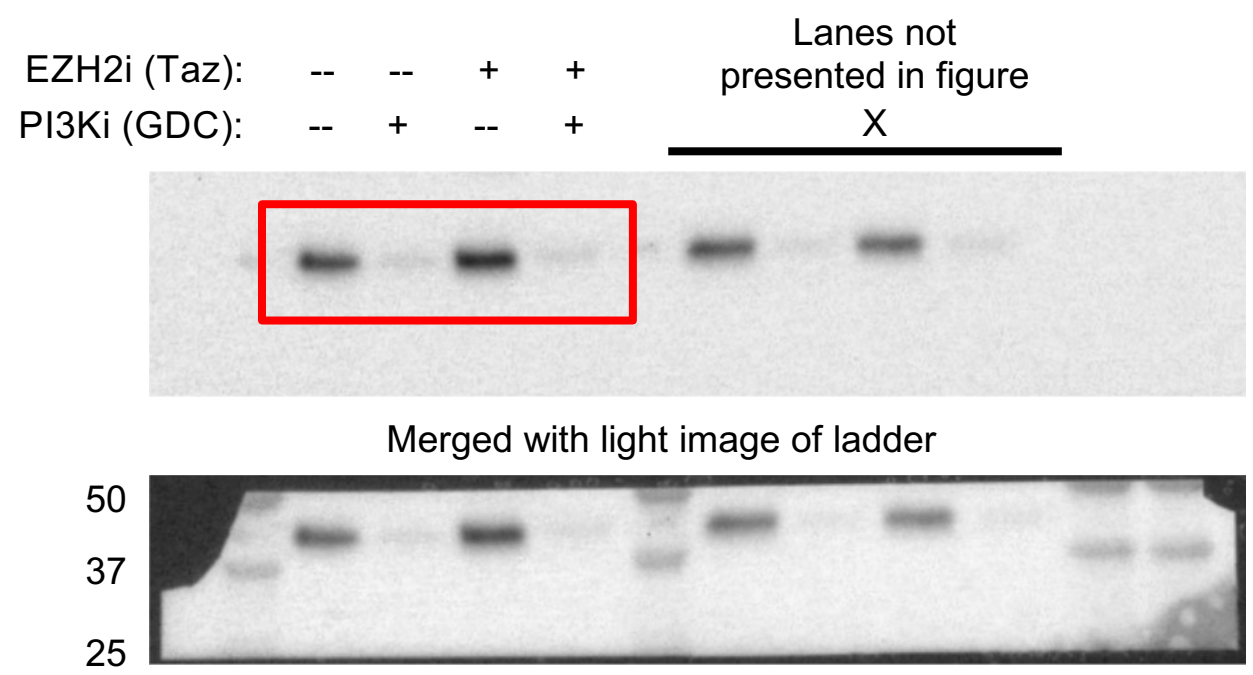

p-PRAS40

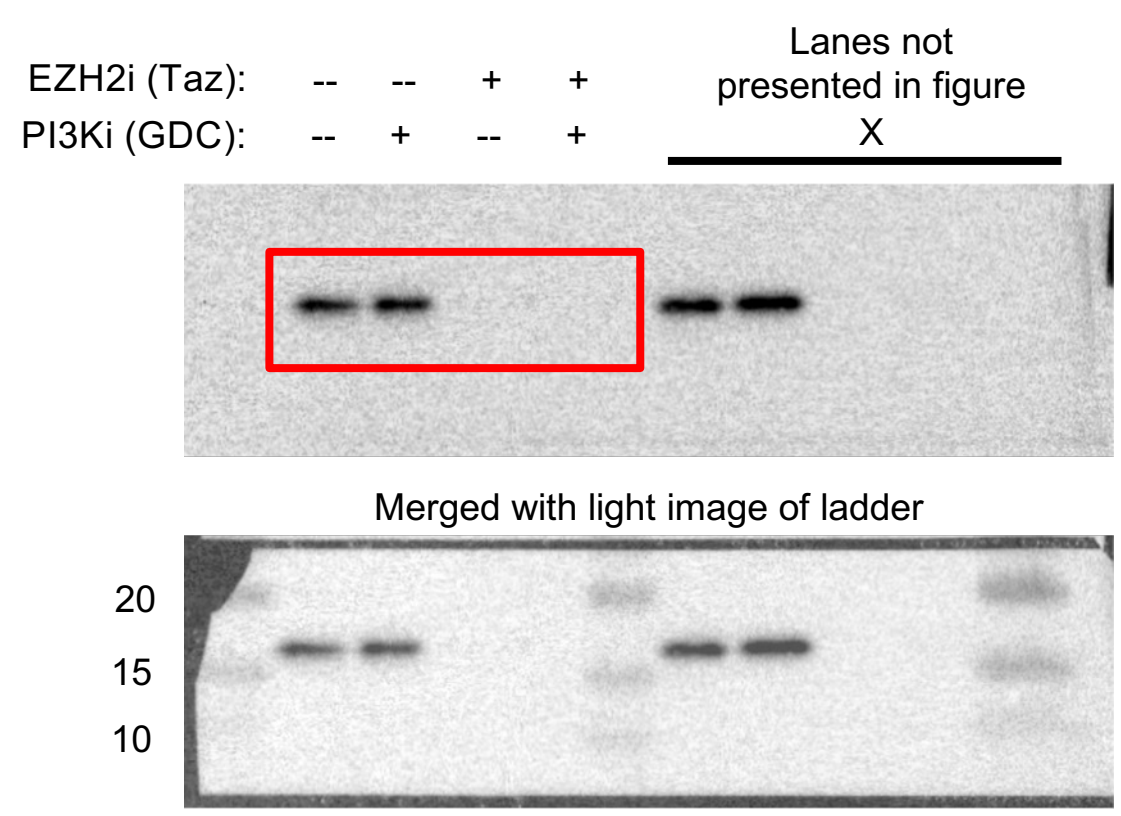

H3K27me3

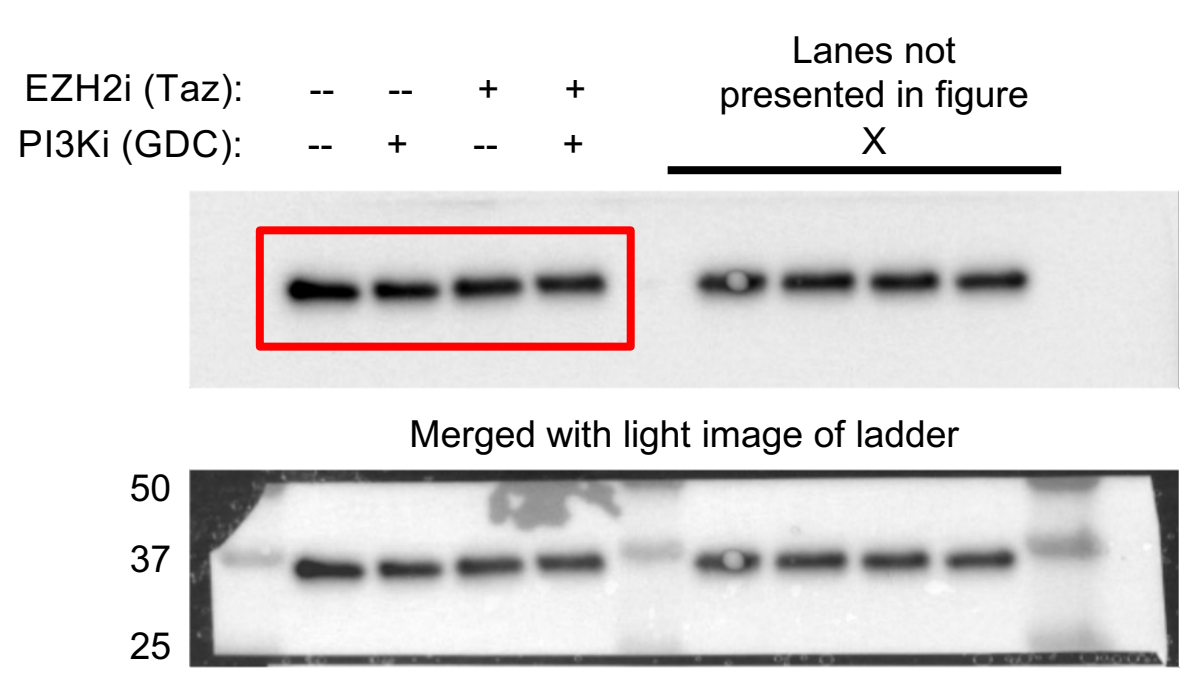

GAPDH

Raw unedited blots for Figure 1D

22Rv1

EZH2i (Taz): -- -- + + | Lanes not presented in figure X
AKTi (Ipata): -- + -- +

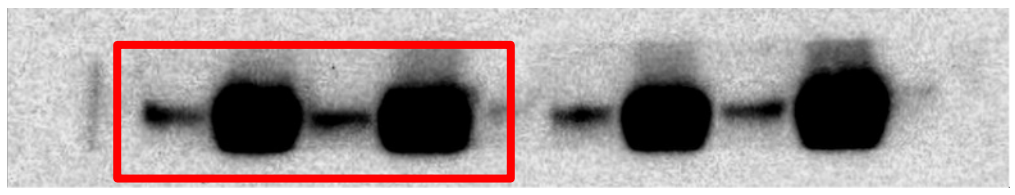

Merged with light image of ladder

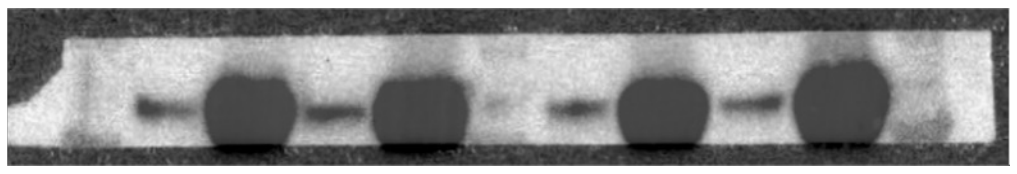

p-AKT

EZH2i (Taz): -- -- + + | Lanes not presented in figure X
AKTi (Ipata): -- + -- +

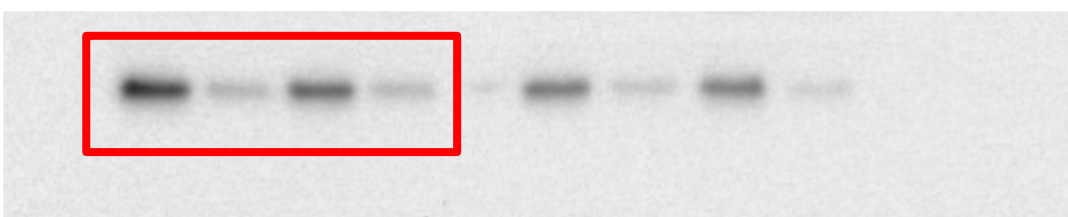

Merged with light image of ladder

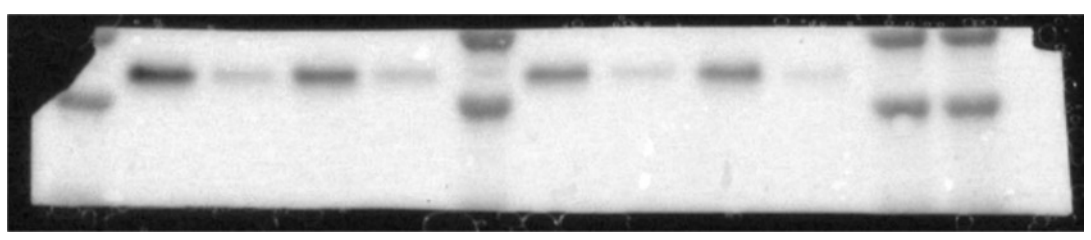

p-PRAS40

EZH2i (Taz): -- -- + + | Lanes not presented in figure X
AKTi (Ipata): -- + -- +

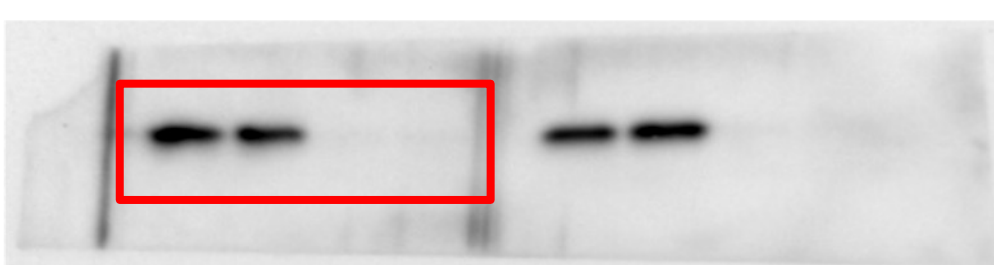

Merged with light image of ladder

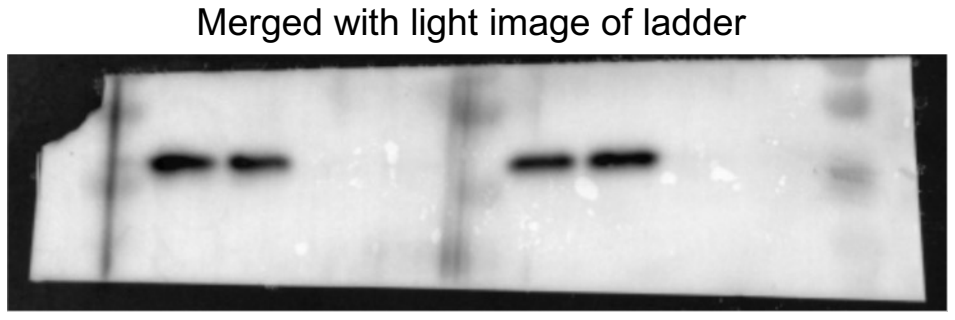

H3K27me3

EZH2i (Taz): -- -- + + | Lanes not presented in figure X
AKTi (Ipata): -- + -- +

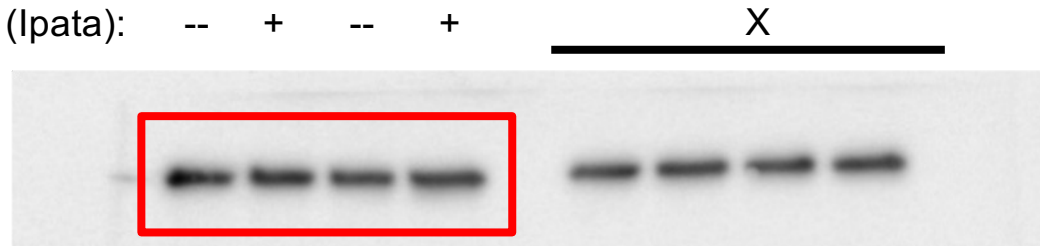

Merged with light image of ladder

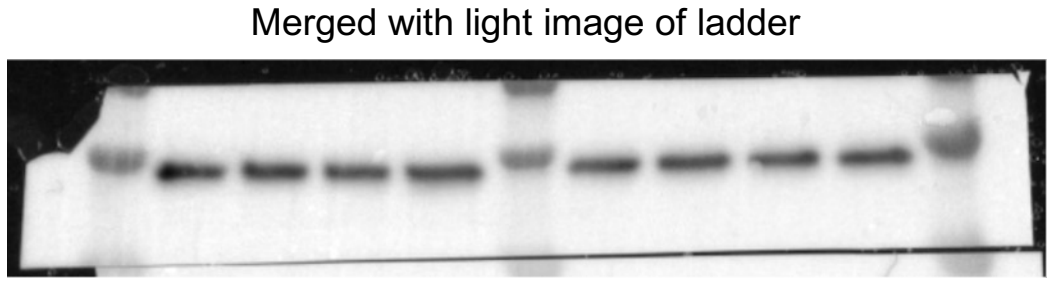

GAPDH

EZH2i (Taz): -- -- + + | Lanes not presented in figure X
AKTi (Ipata): -- + -- +

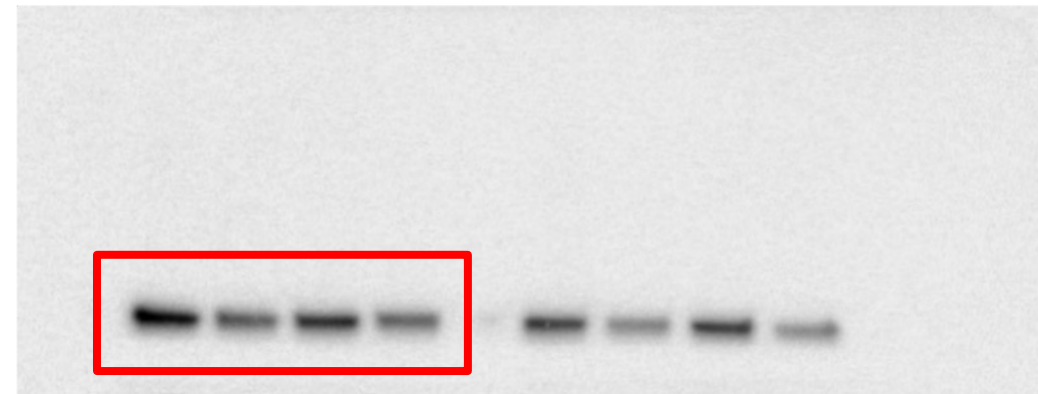

Merged with light image of ladder

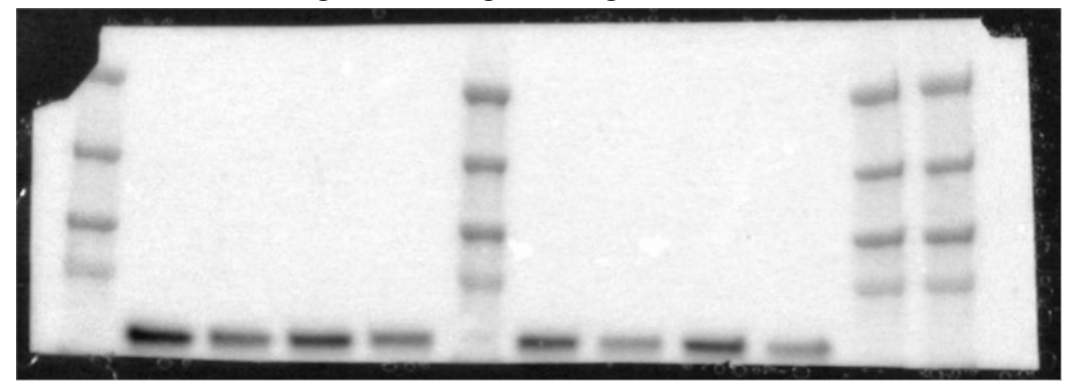

AKT

EZH2i (Taz): -- -- + + | Lanes not presented in figure X
AKTi (Ipata): -- + -- +

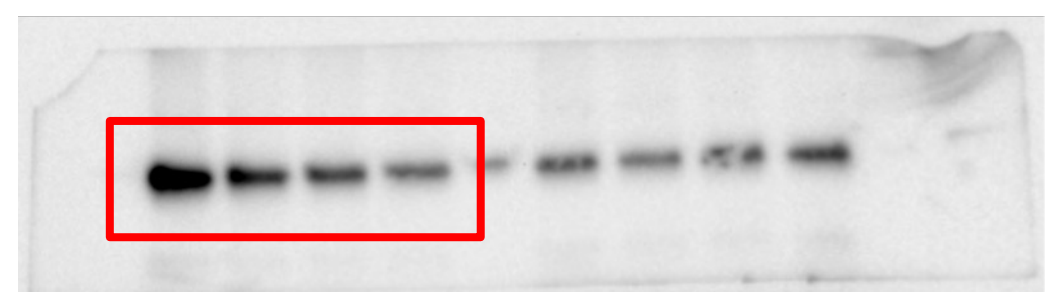

Merged with light image of ladder

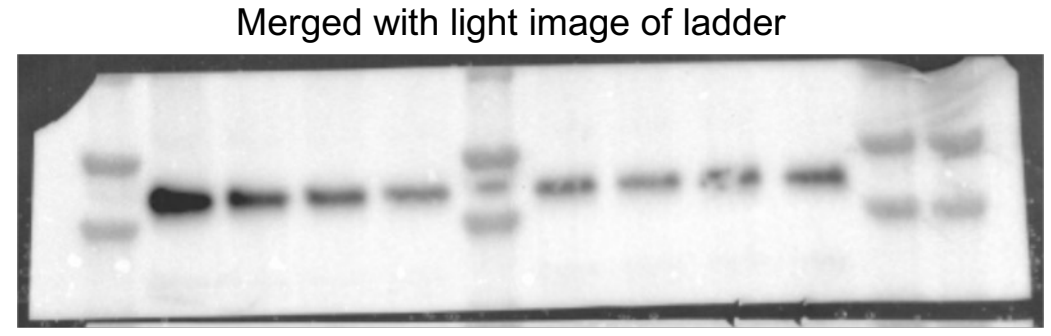

PRAS40

EZH2i (Taz): -- -- + + | Lanes not presented in figure X
AKTi (Ipata): -- + -- +

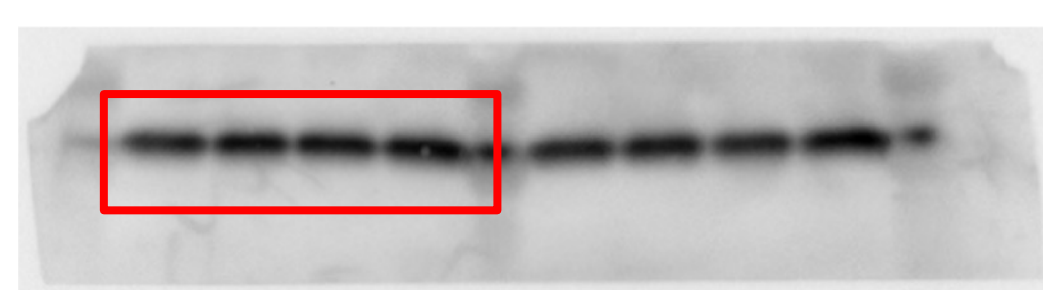

Merged with light image of ladder

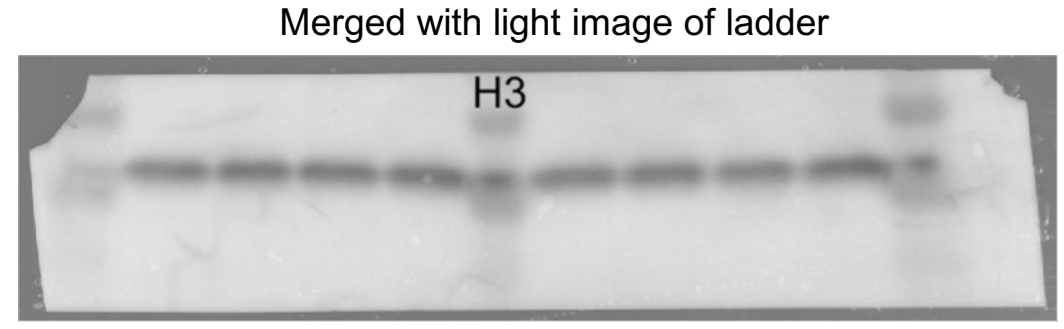

H3

Raw unedited blots for Figure 1D

22Rv1

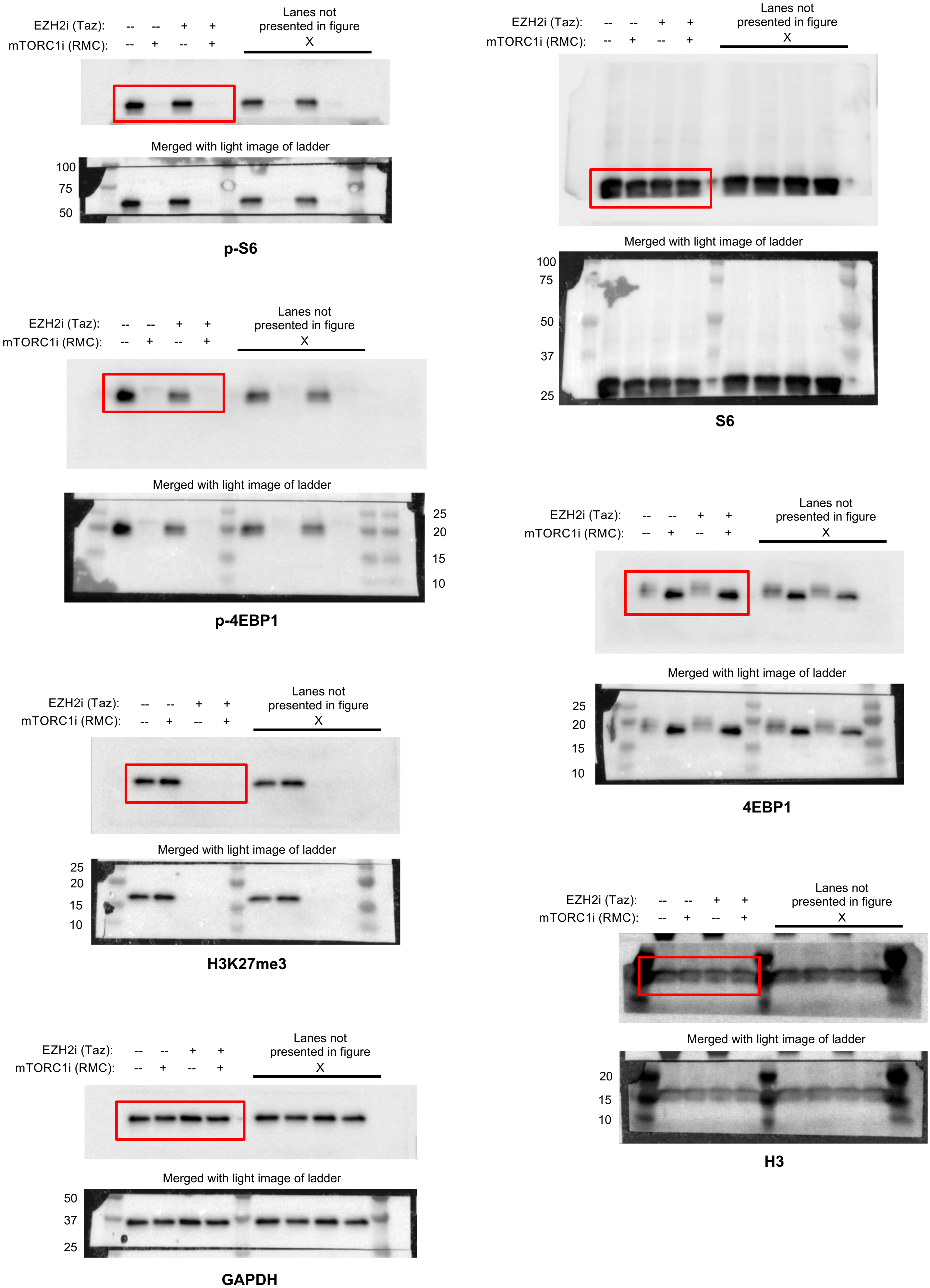

Raw unedited blots for Figure 2F

LuCaP 70-CR PDX

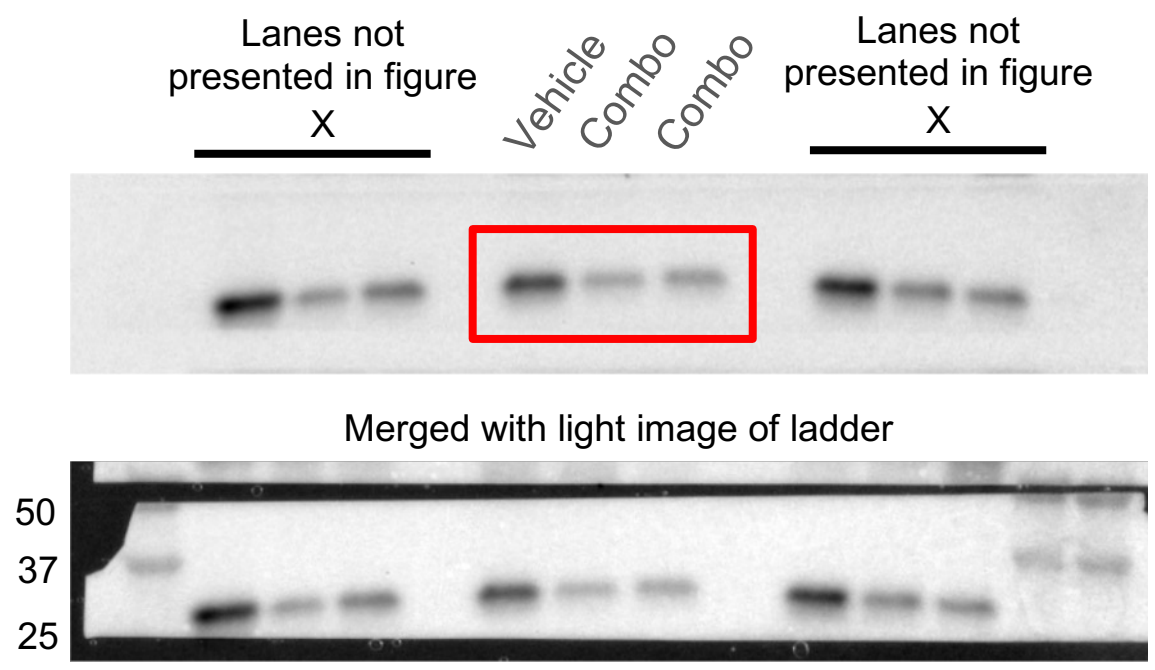

p-S6

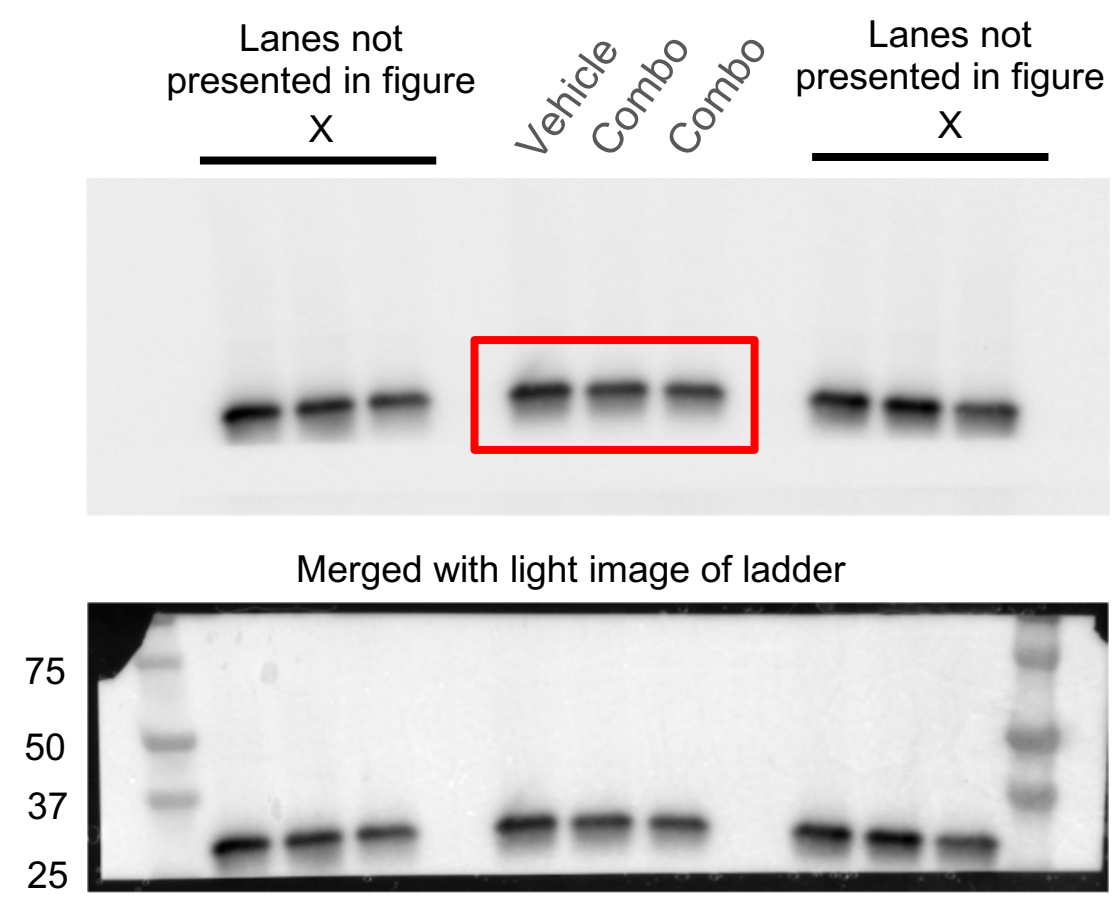

S6

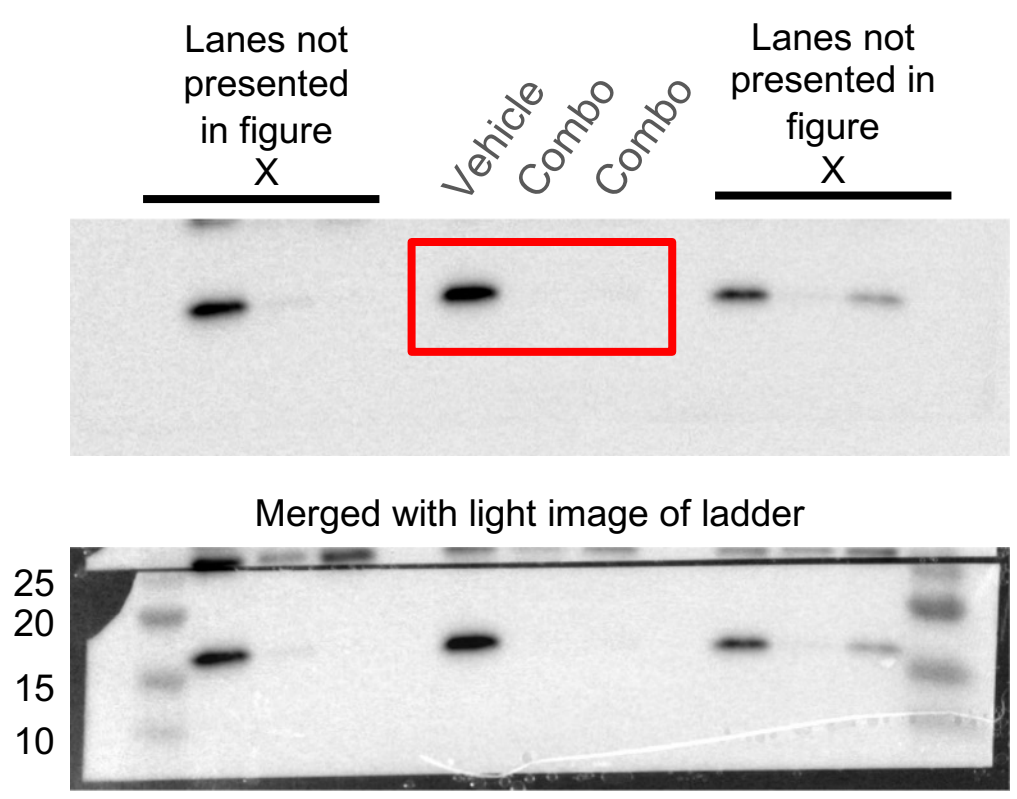

H3K27me3

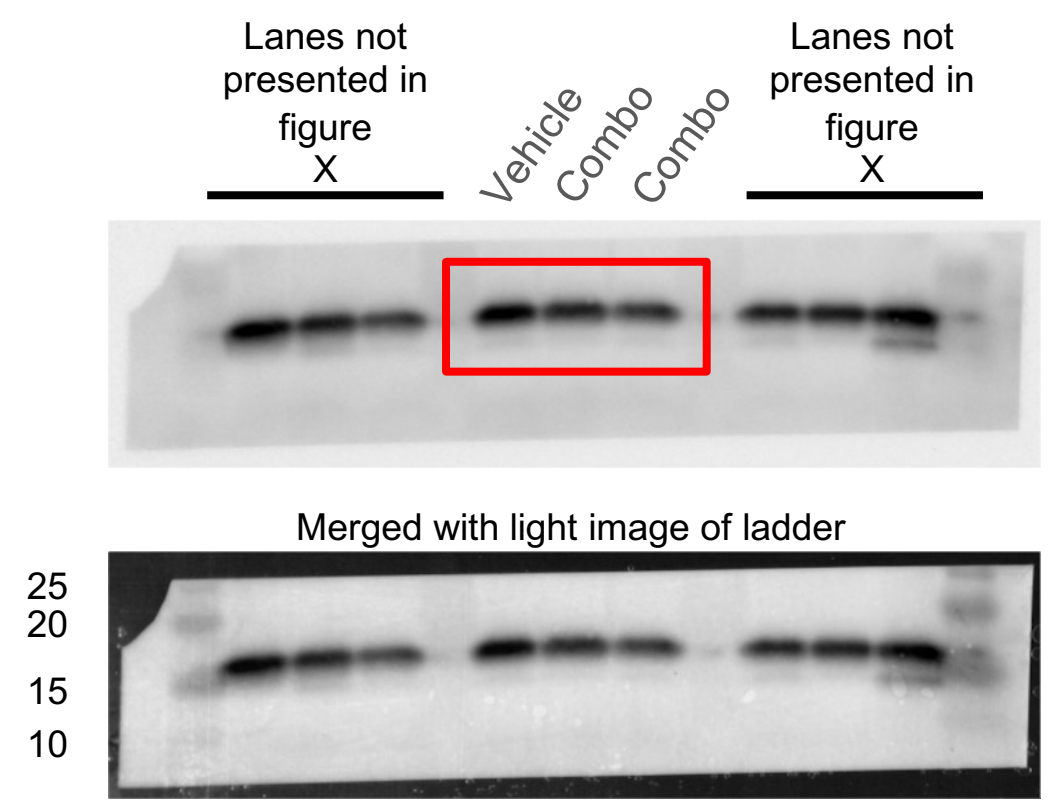

H3

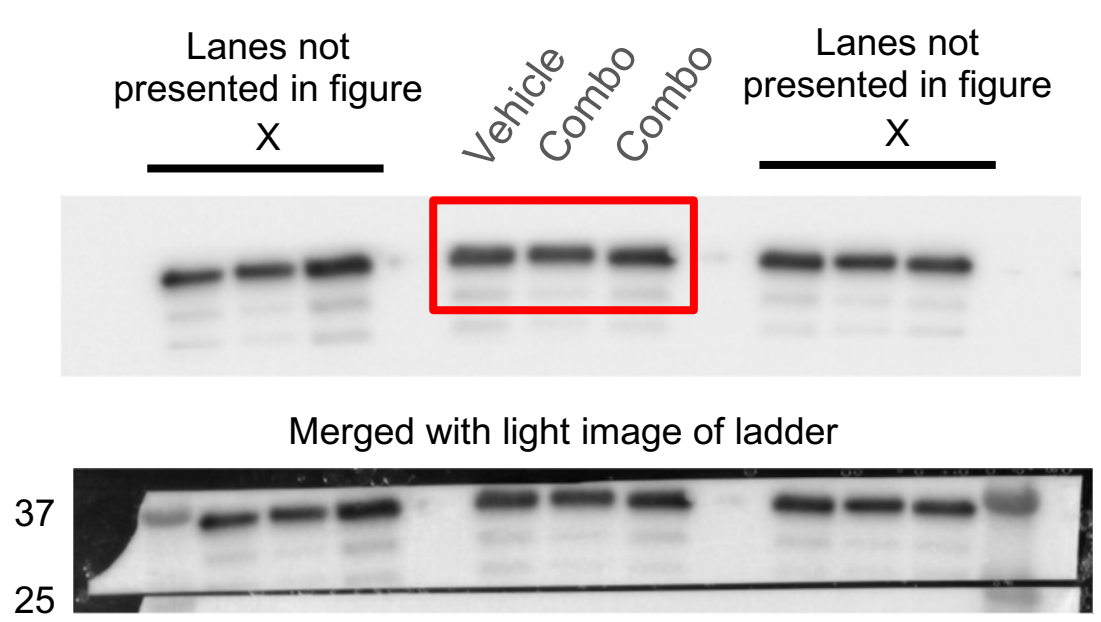

GAPDH

Raw unedited blots for Figure 3E

C4.2B

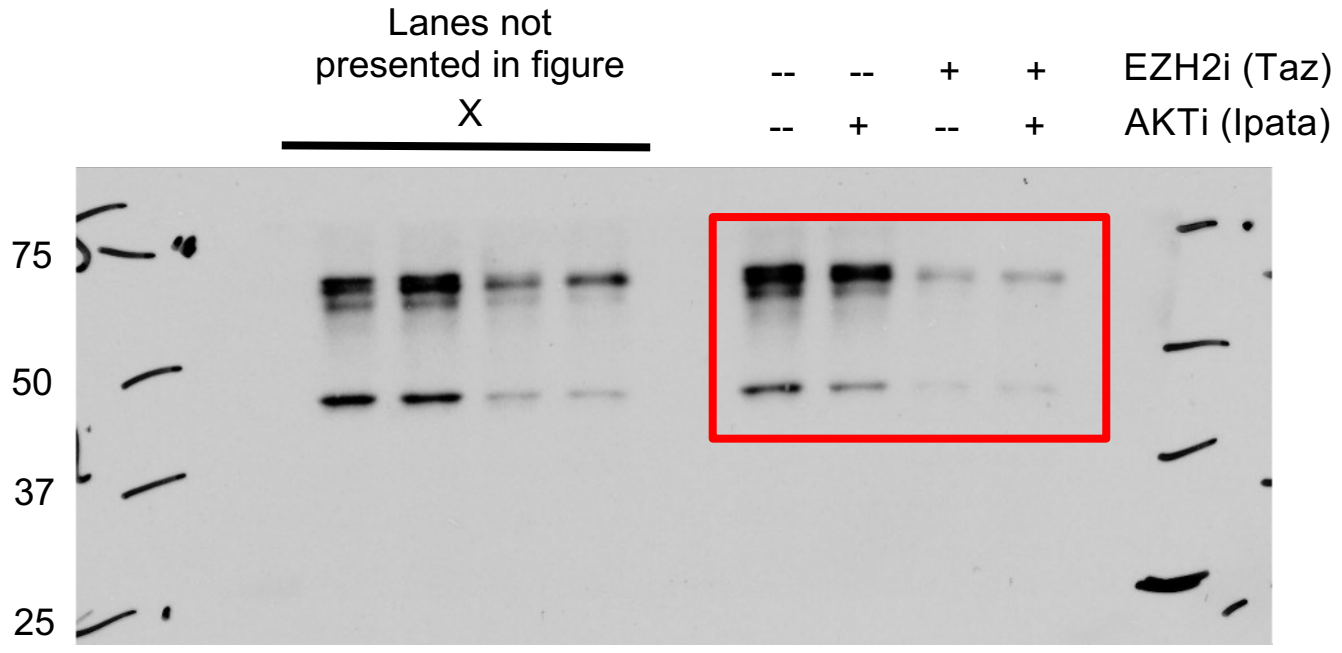

GLUT1

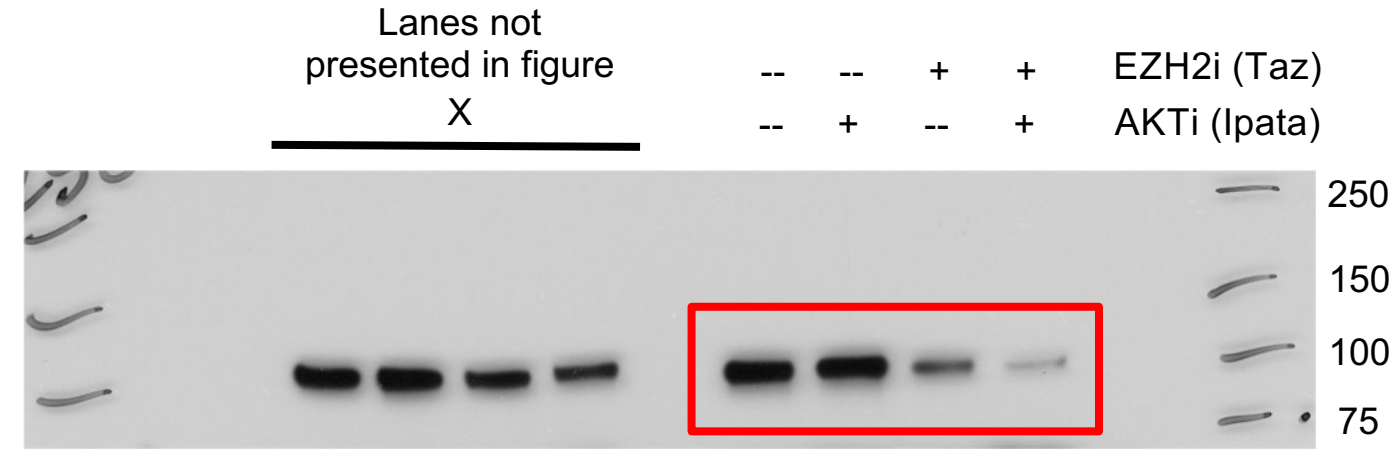

HK2

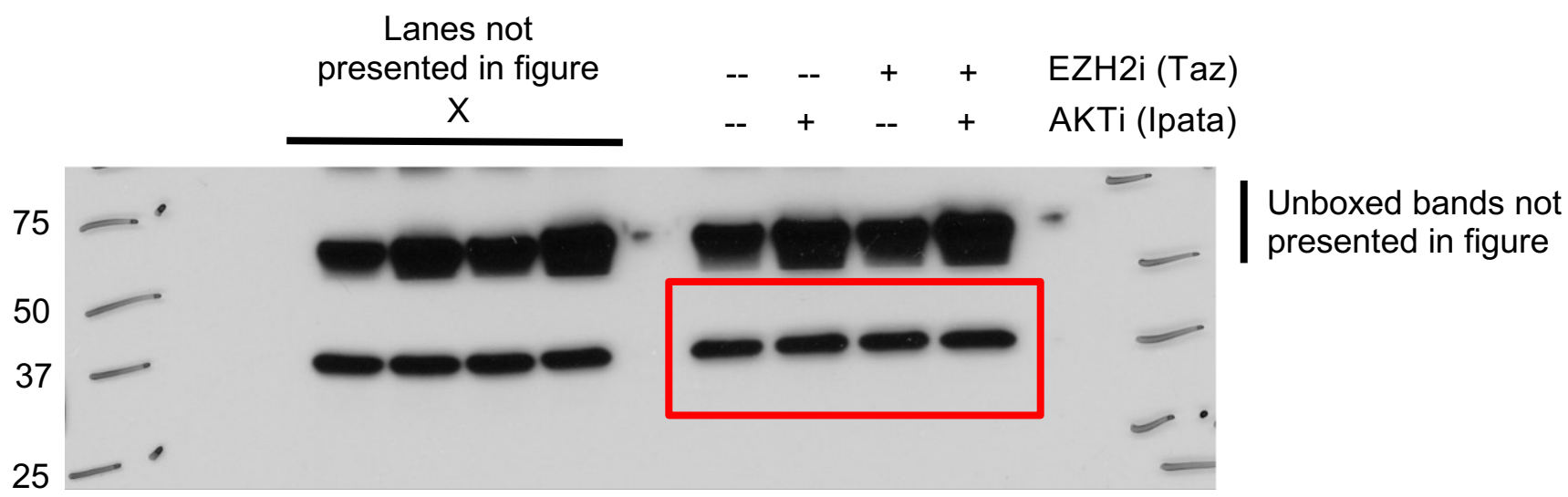

GAPDH

Raw unedited blots for Figure 3F

LuCaP 70-CR PDX

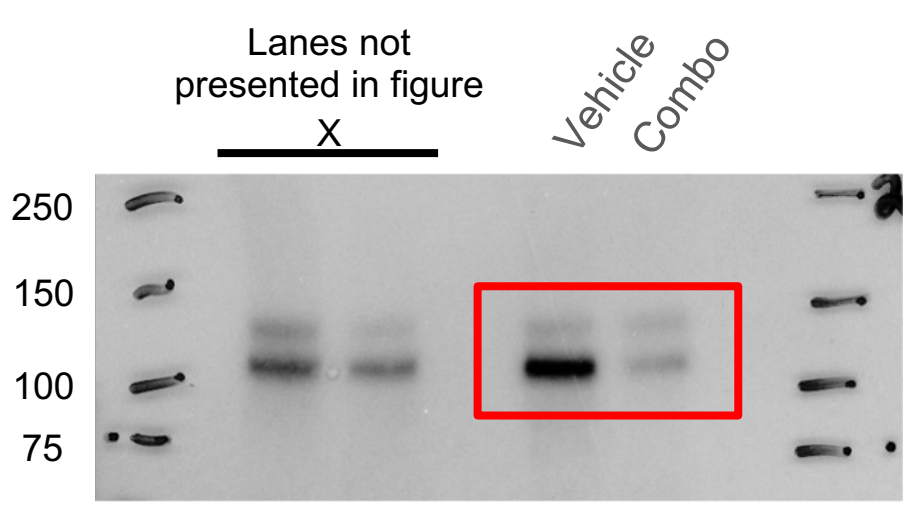

HK2

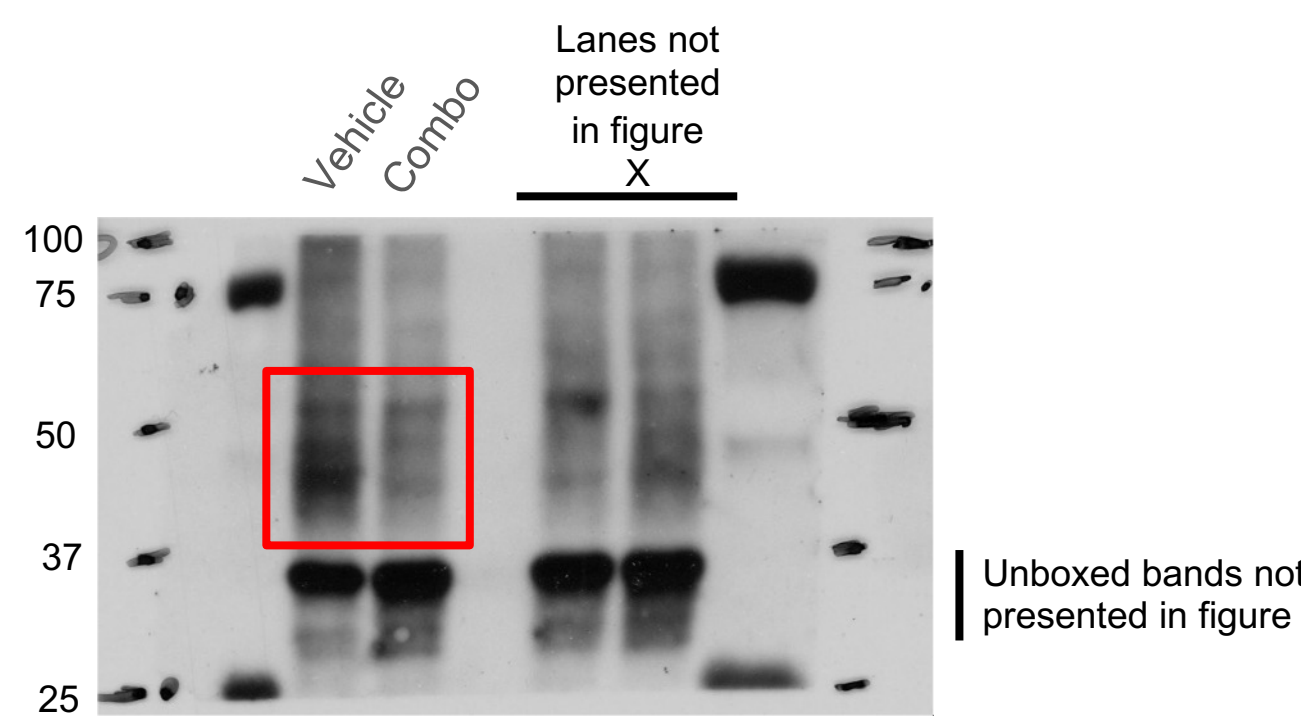

GLUT1

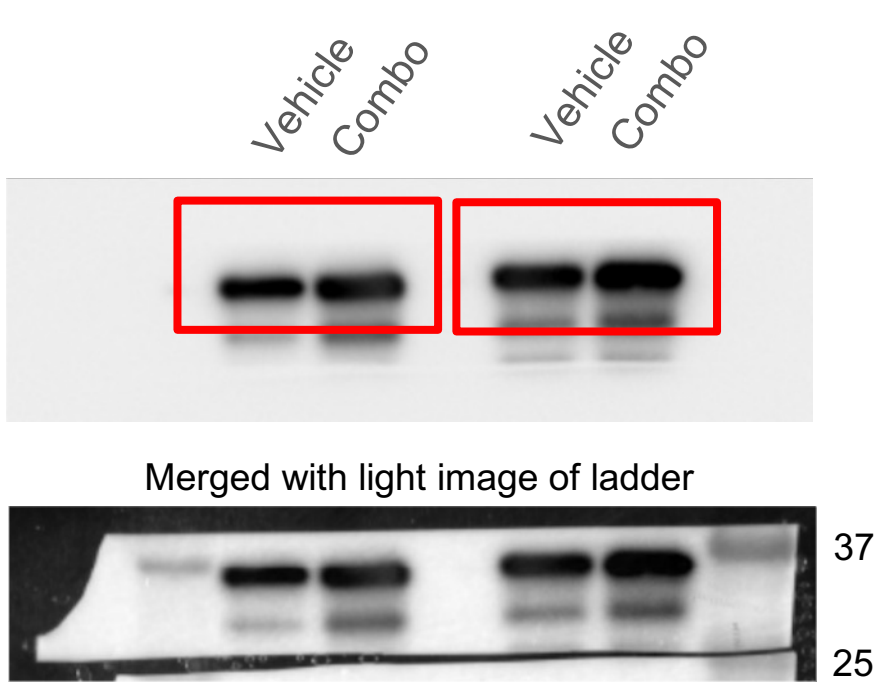

GAPDH

Raw unedited blots for Figure 3M

C4.2B

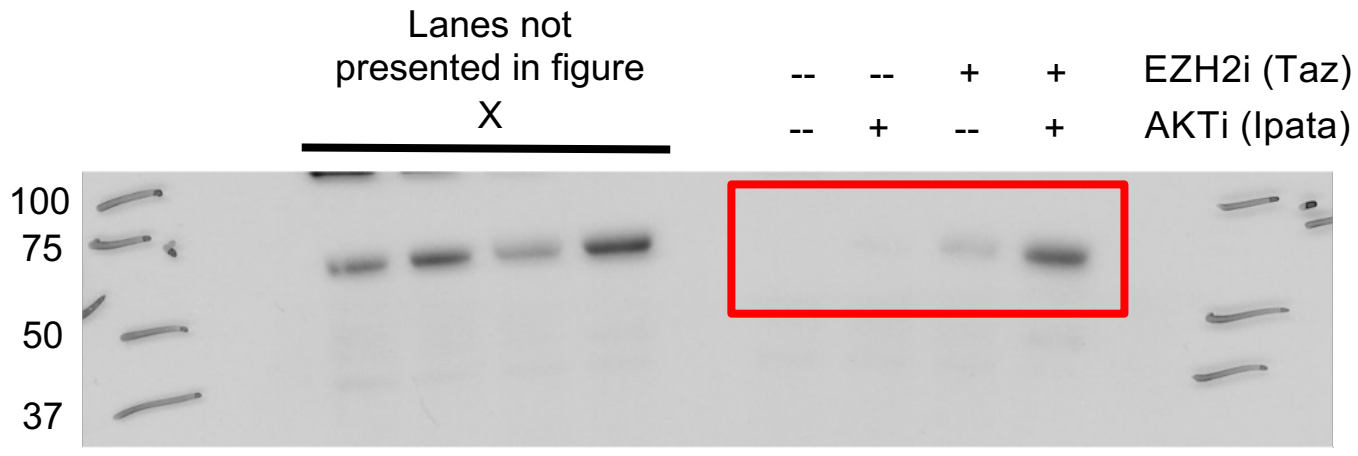

p-AMPK

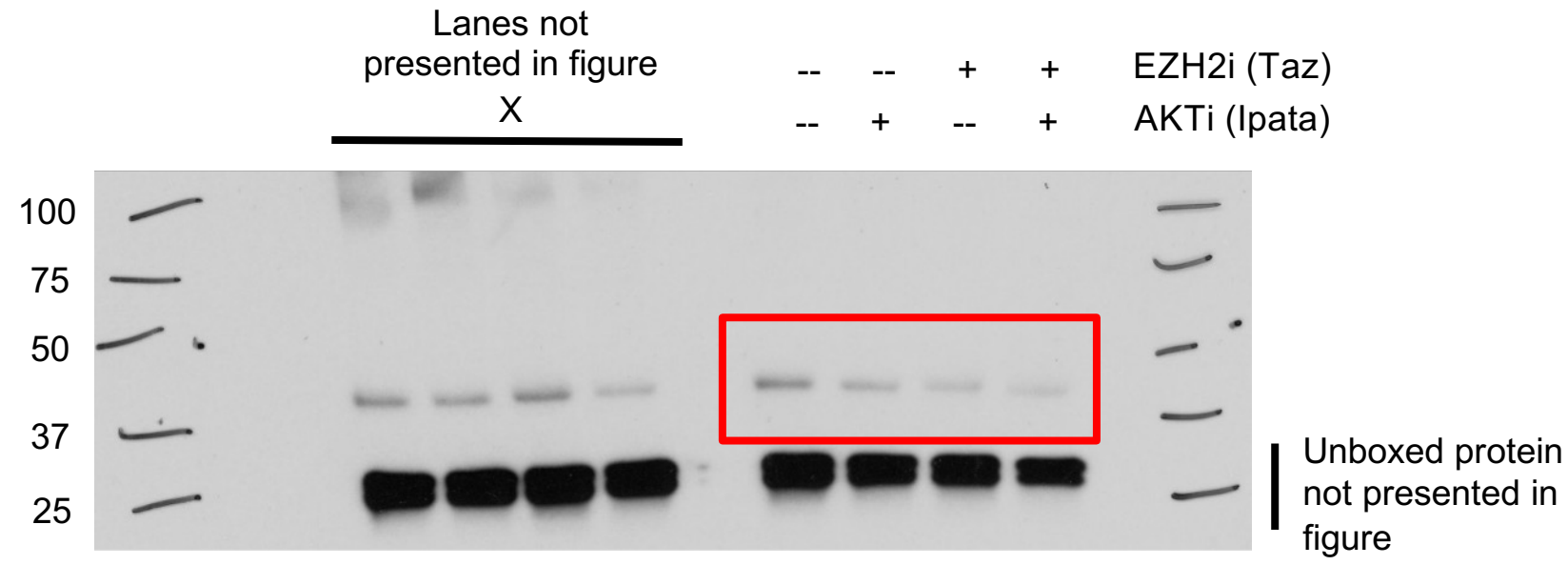

AMPK

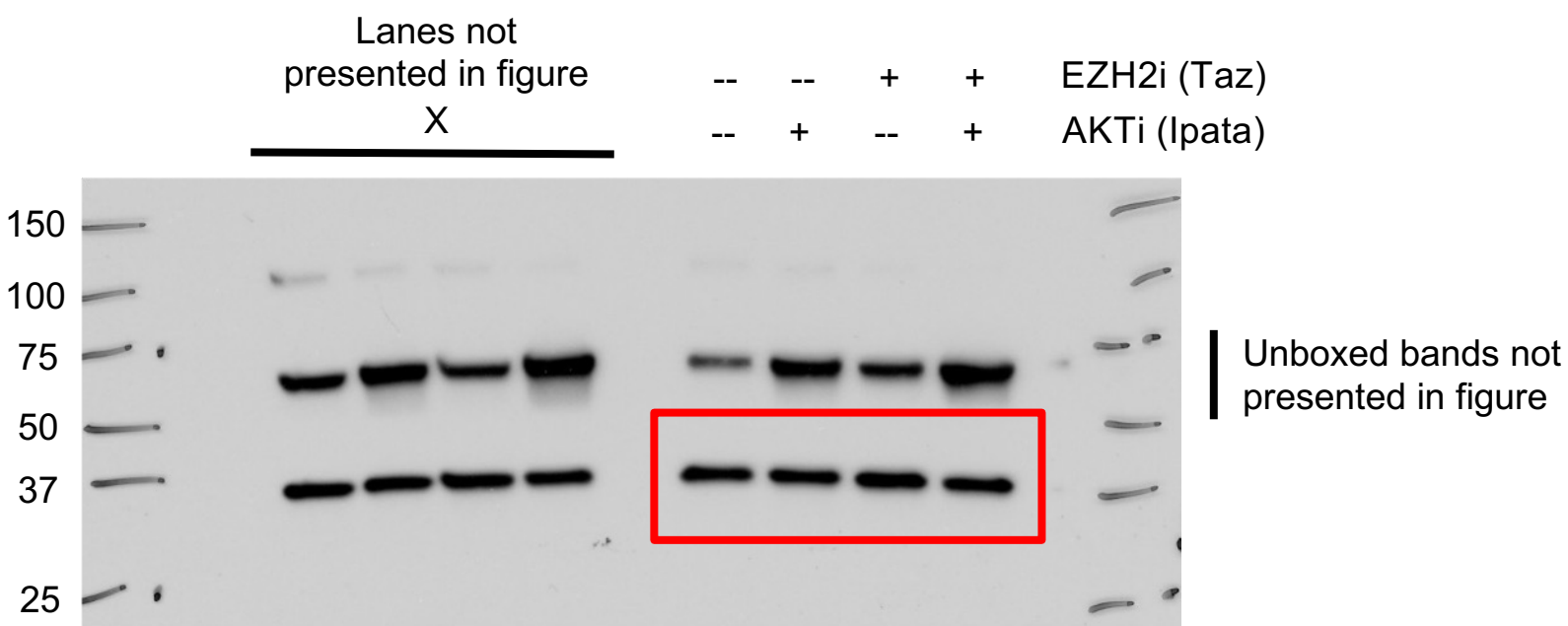

GAPDH

Raw unedited blots for Figure 3N

LuCaP 70-CR PDX

Note: same experiment and GAPDH membrane as Figure 2F but different lanes were used

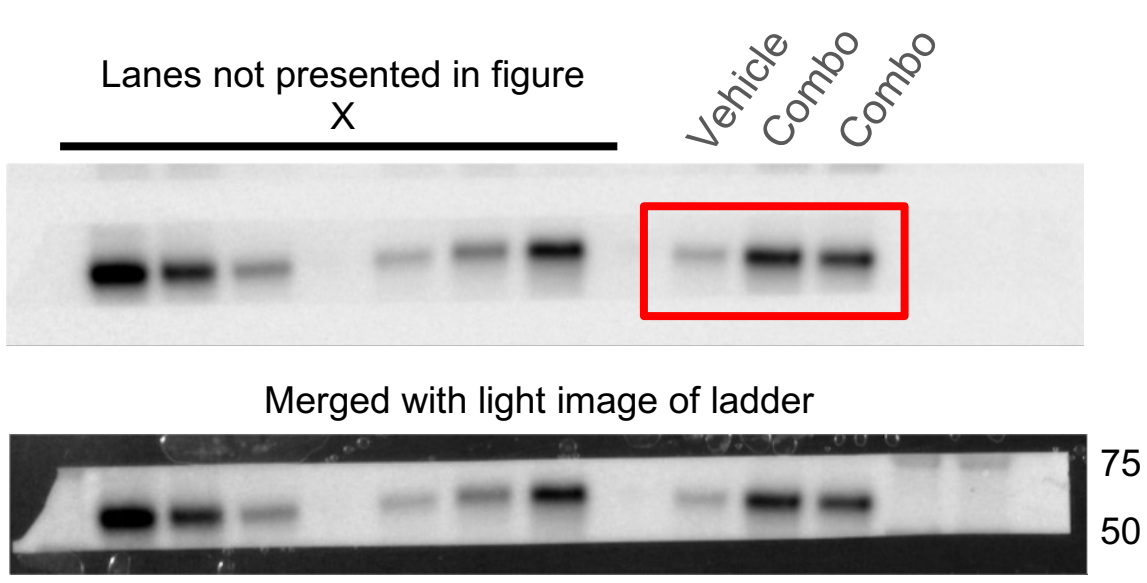

p-AMPK

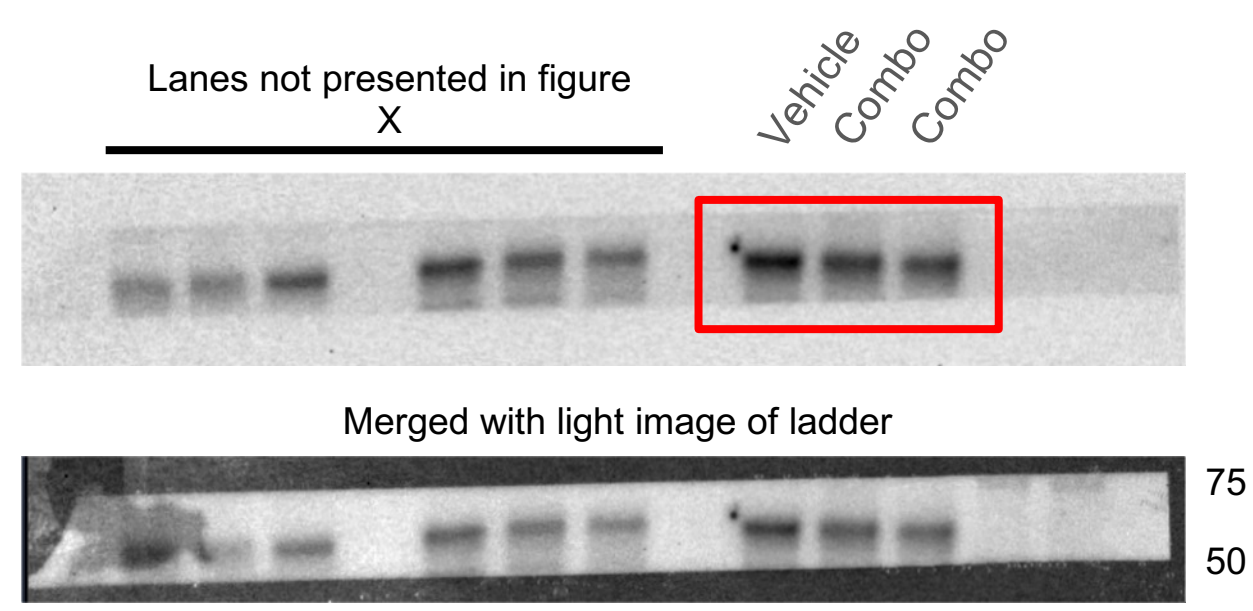

AMPK

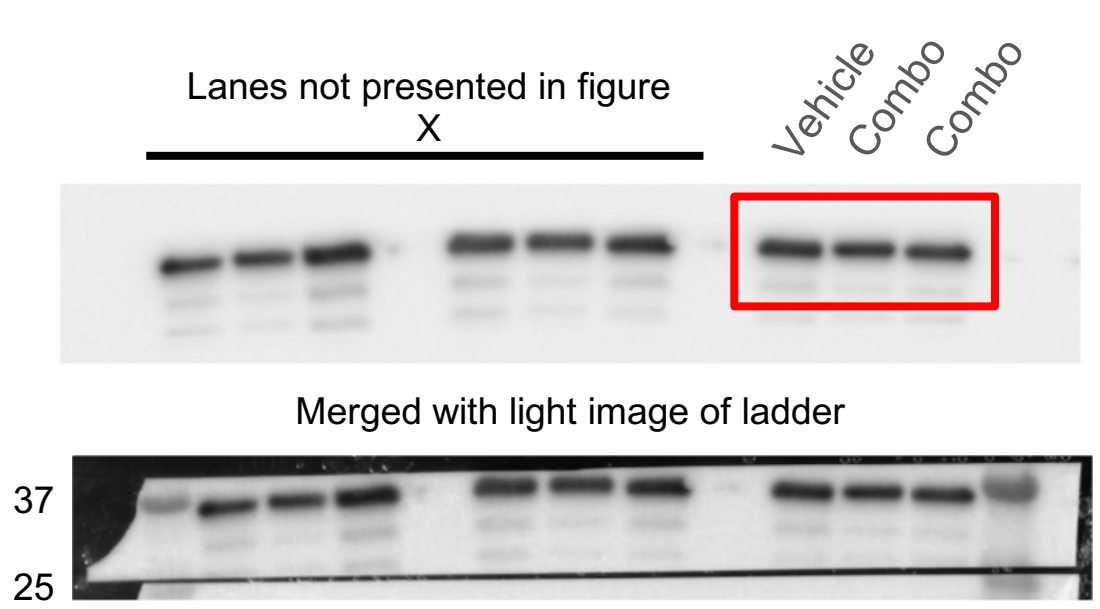

GAPDH

Raw unedited blots for Figure 4C

C4.2B

Note: for 9h - same experiment and GAPDH membrane as Figure 3M but different lanes were used

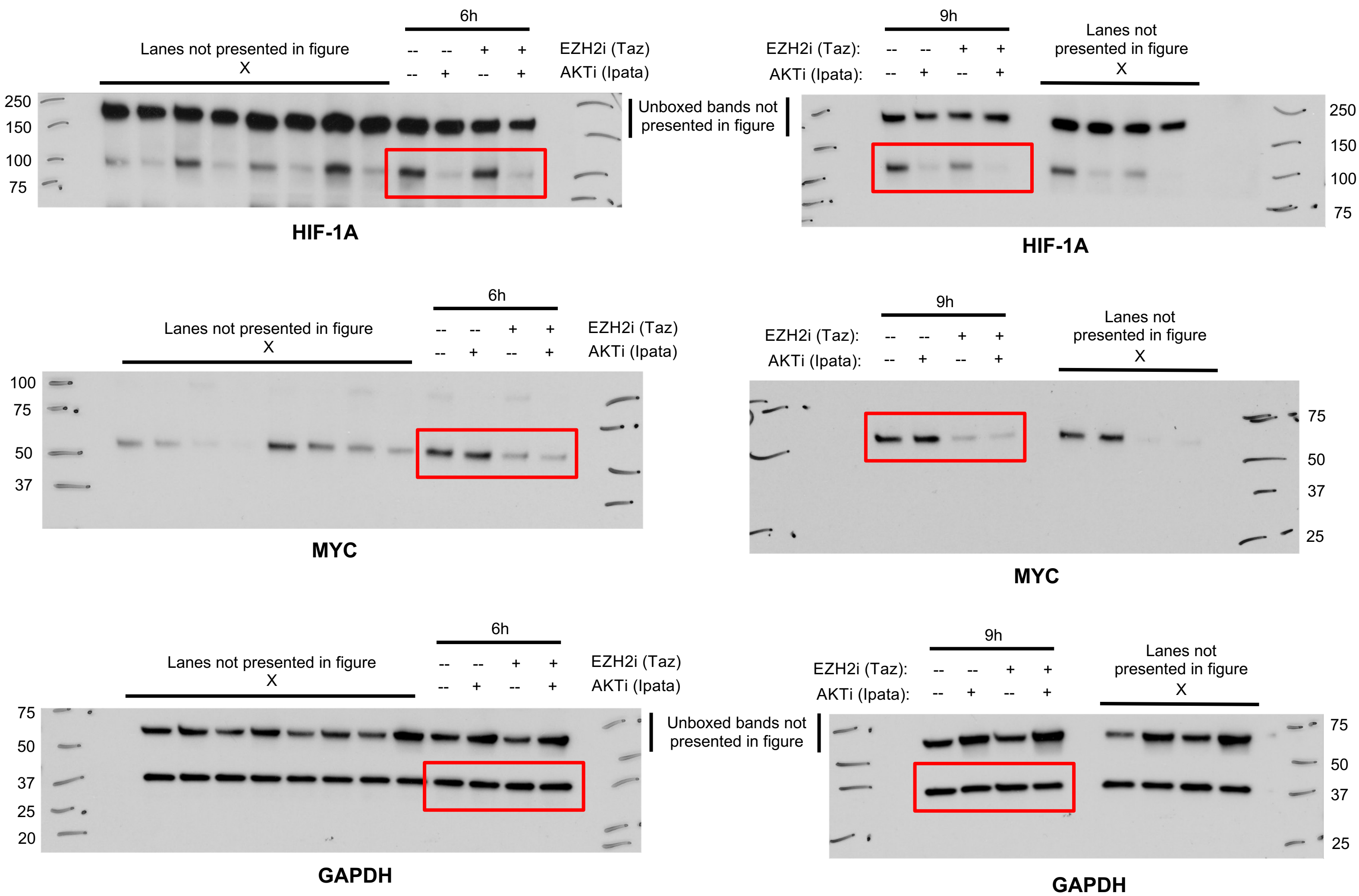

Raw unedited blots for Figure 4D

LuCaP 70-CR PDX

Note: same experiment and same GAPDH membrane as Figure 3F

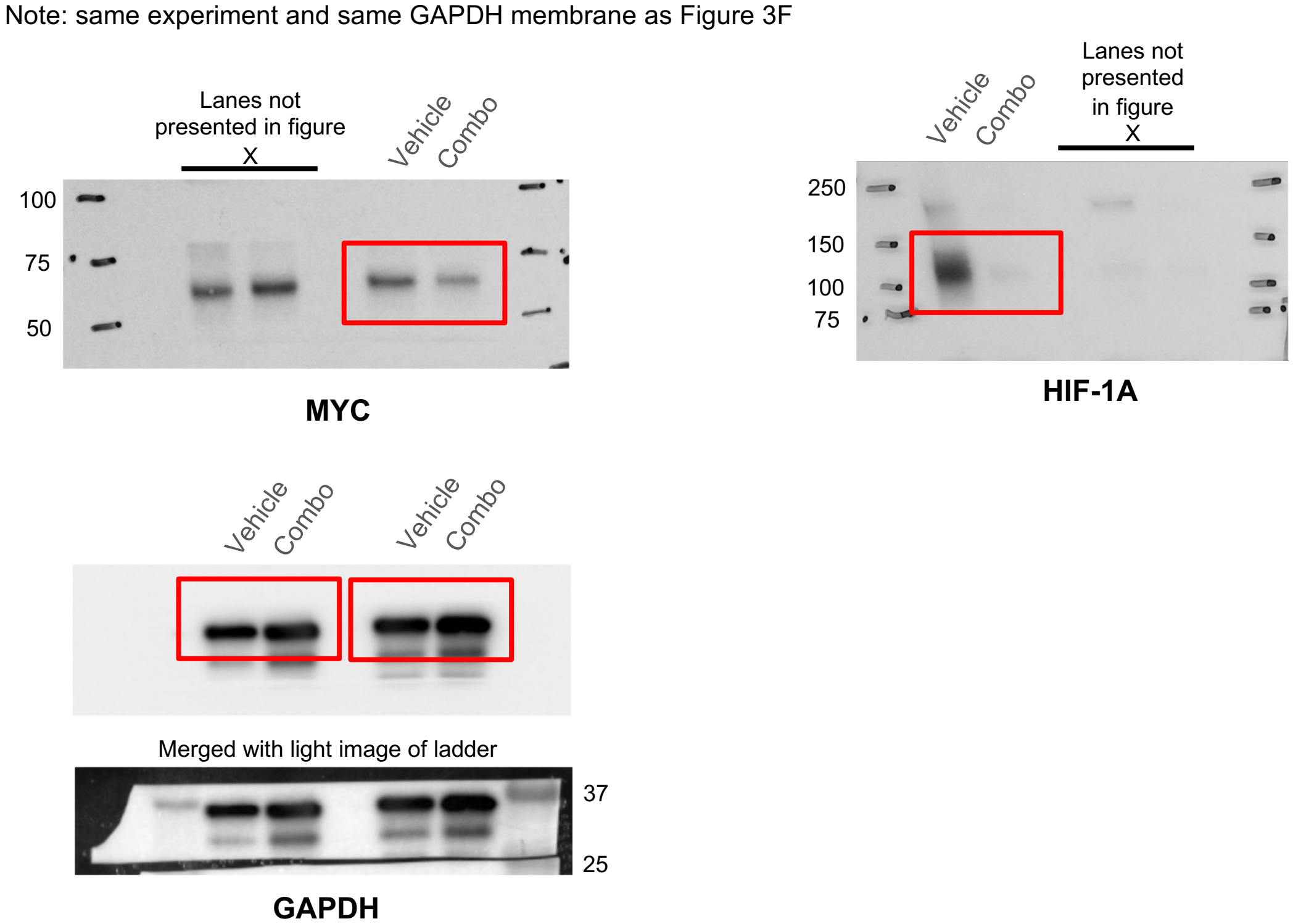

Raw unedited blots for Figure 4F

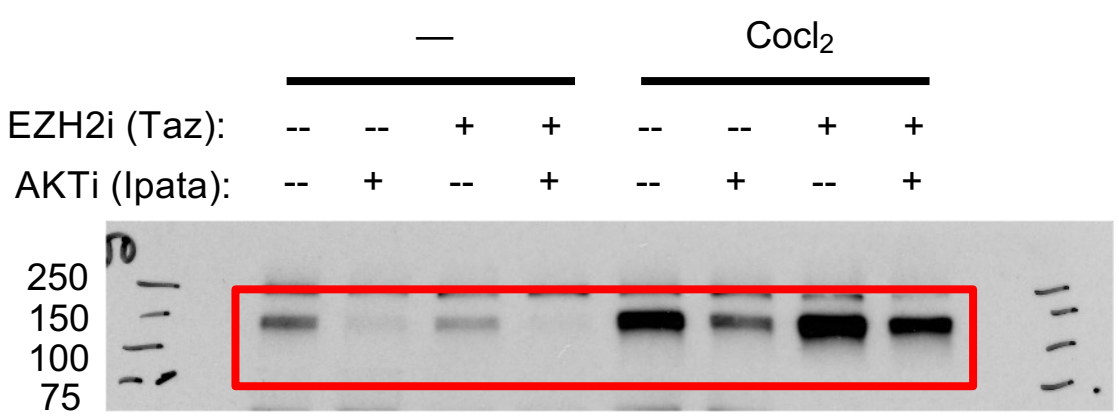

HIF-1A

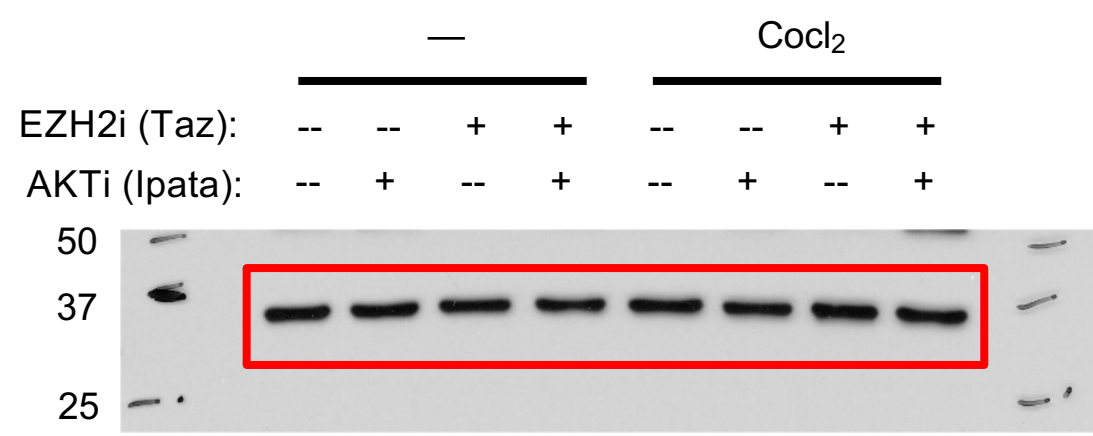

GAPDH

Raw unedited blots for Figure 4H

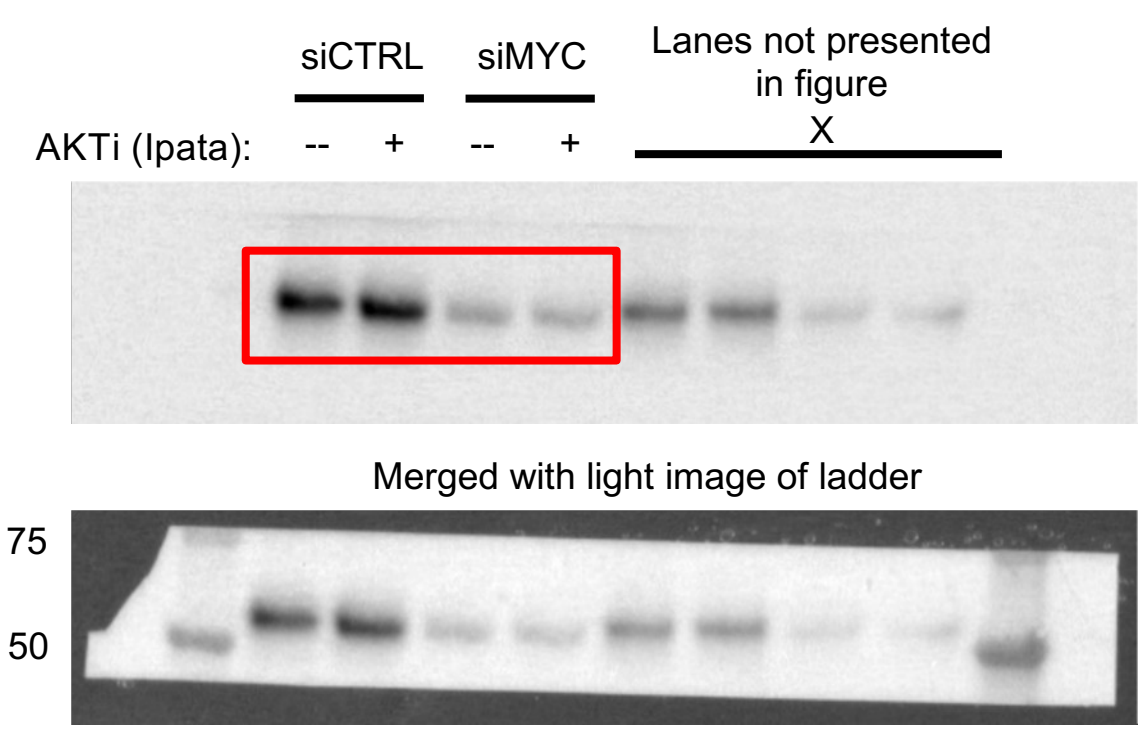

MYC

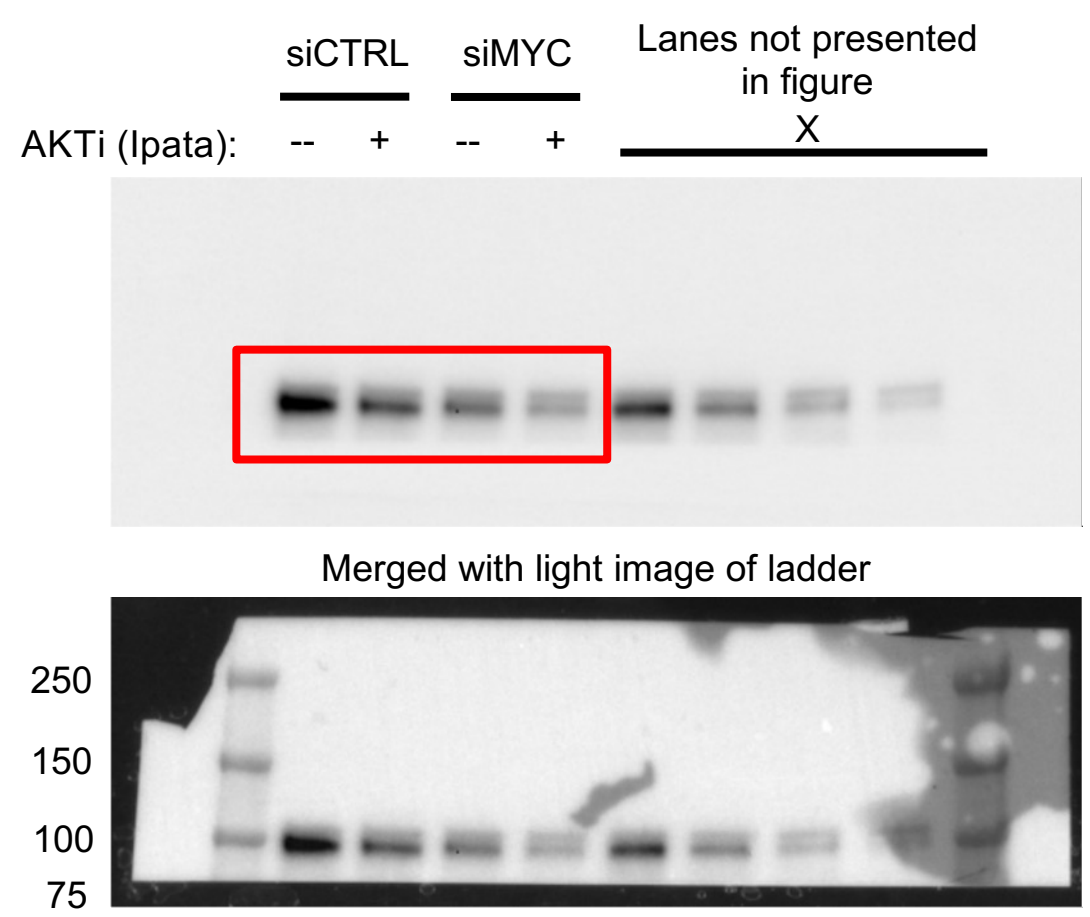

HK2

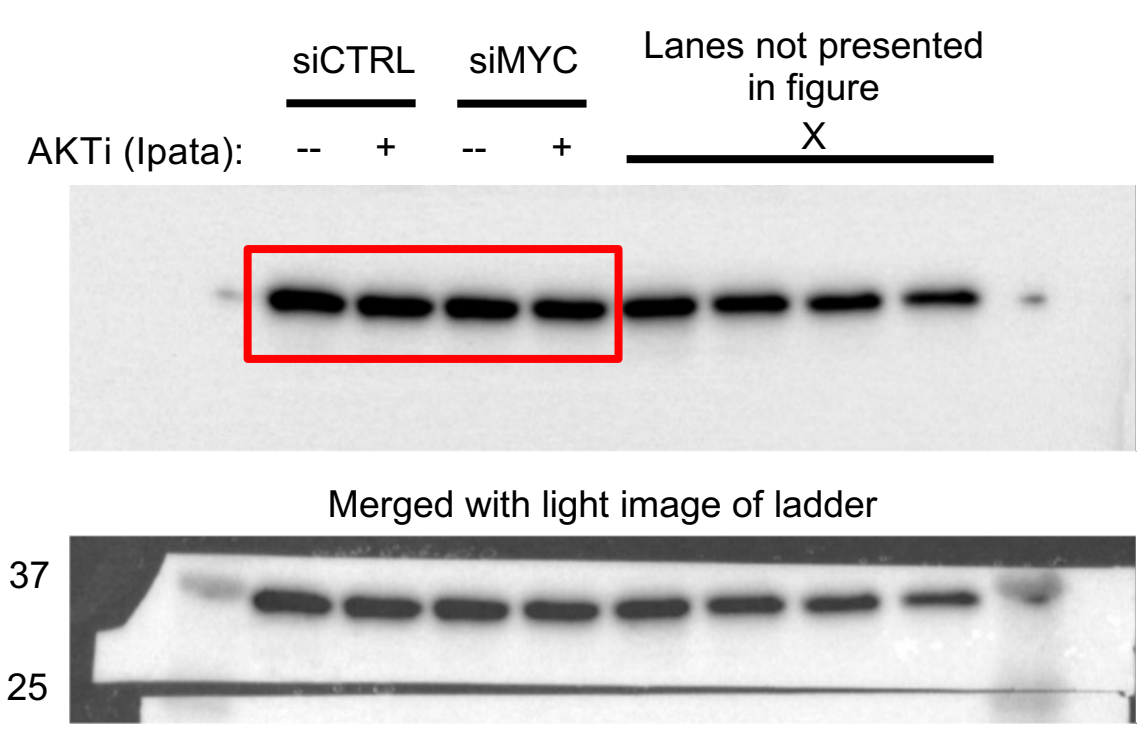

GAPDH

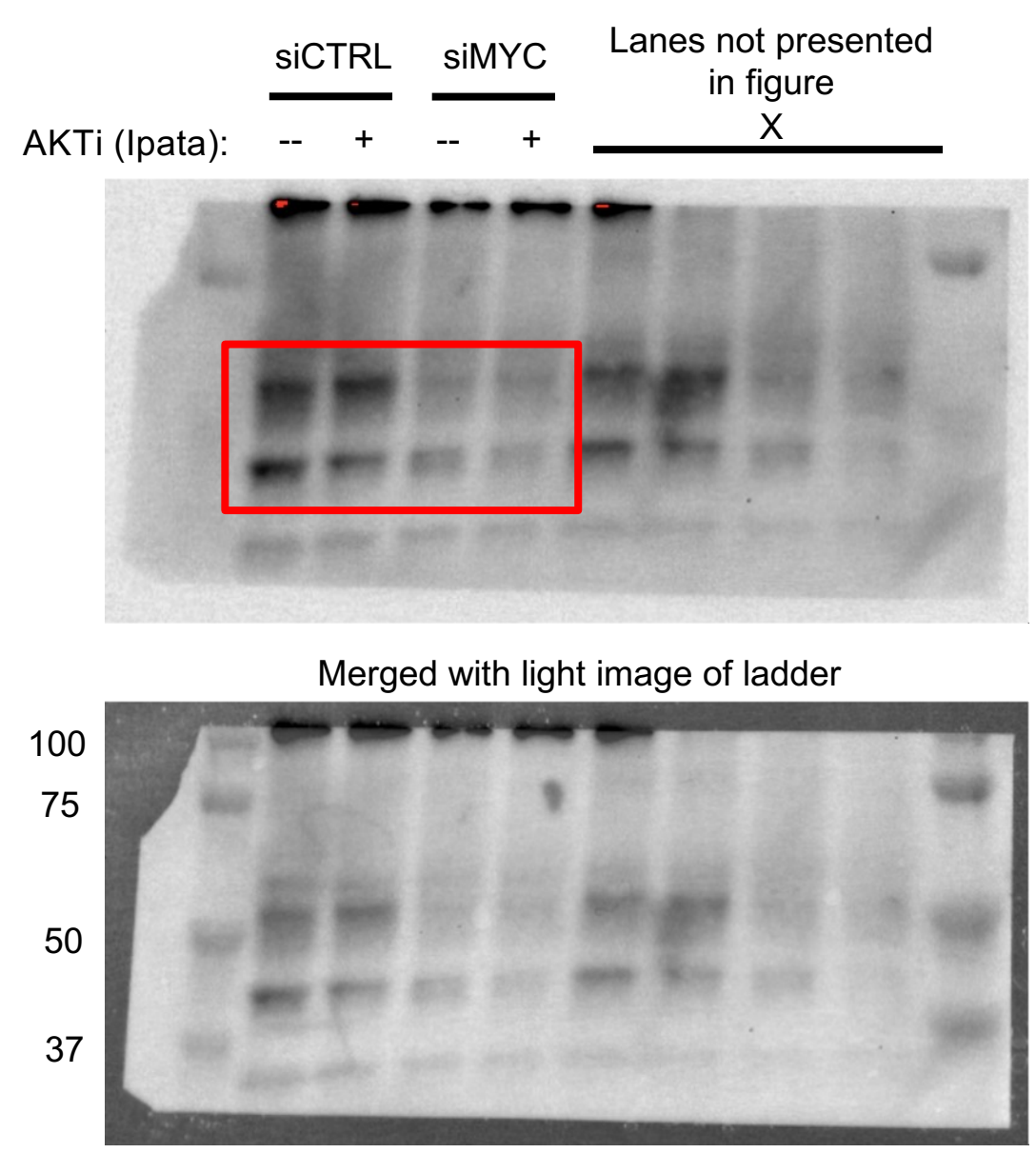

GLUT1

Raw unedited blots for Figure 5D

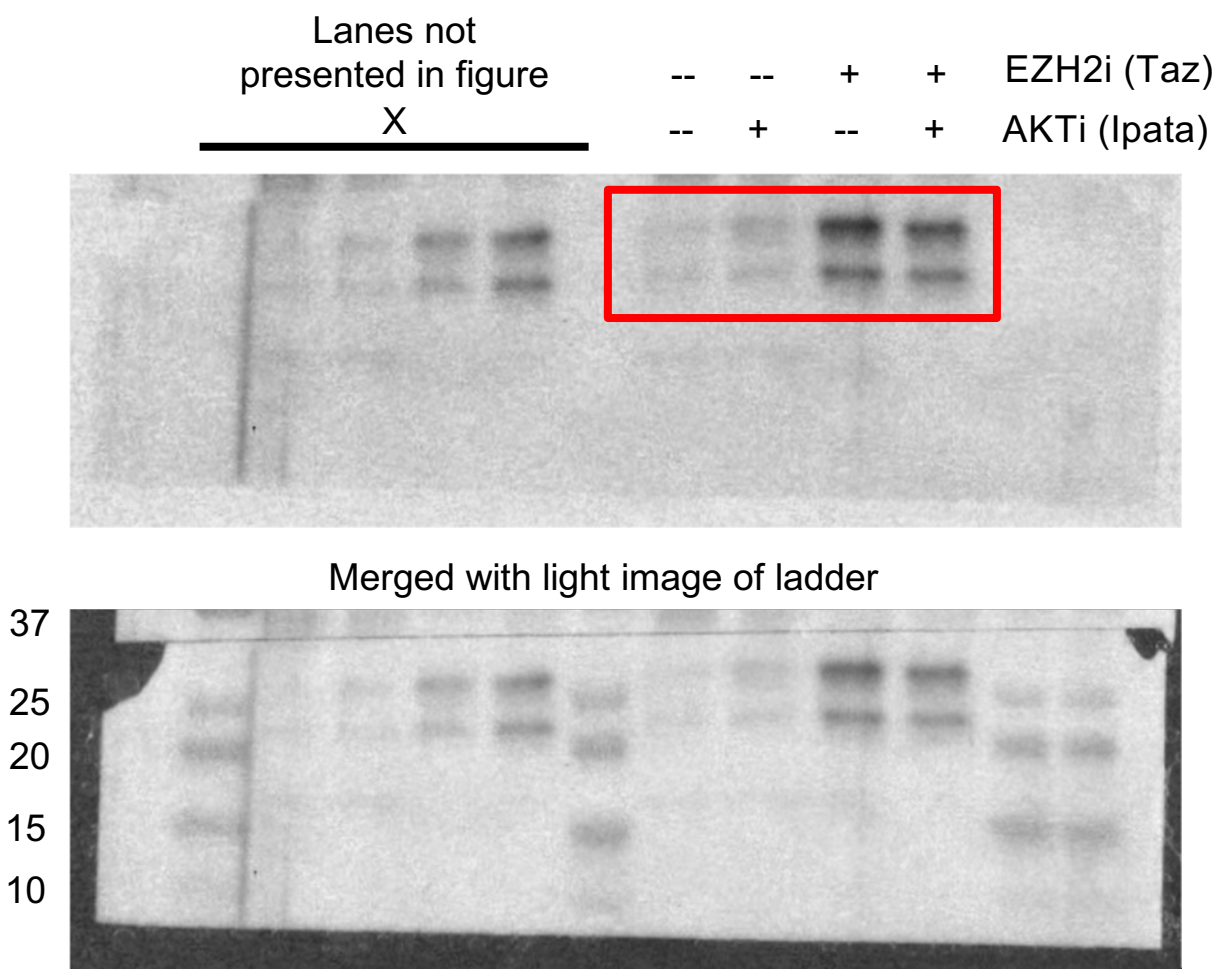

BMF

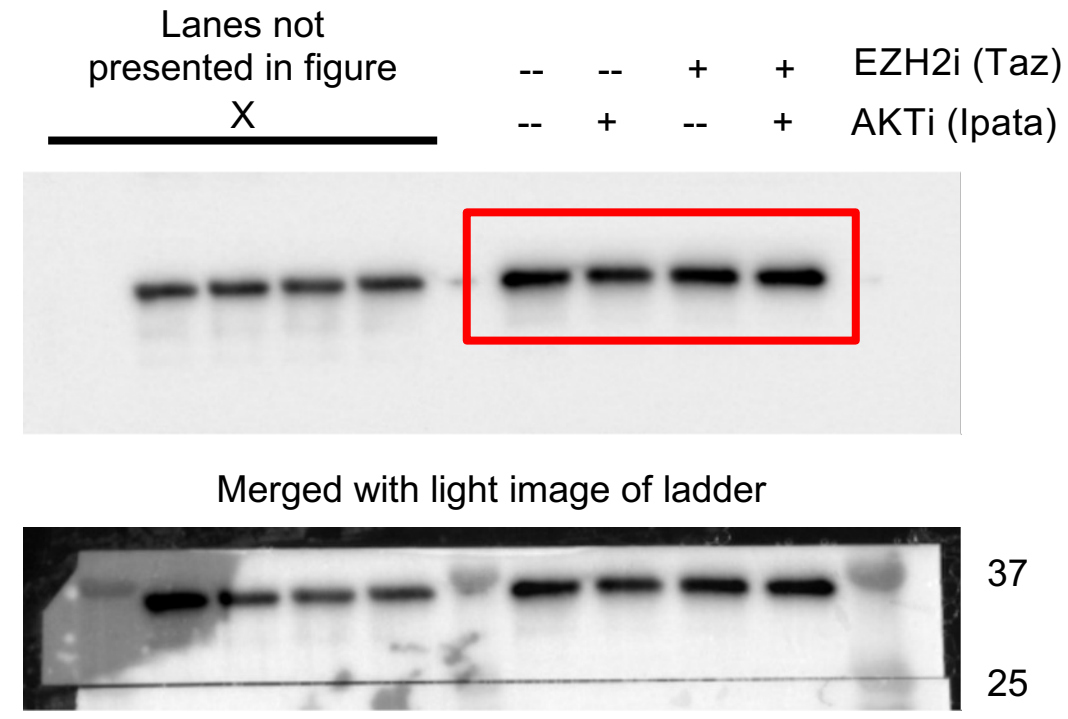

GAPDH

Raw unedited blots for Figure 5F

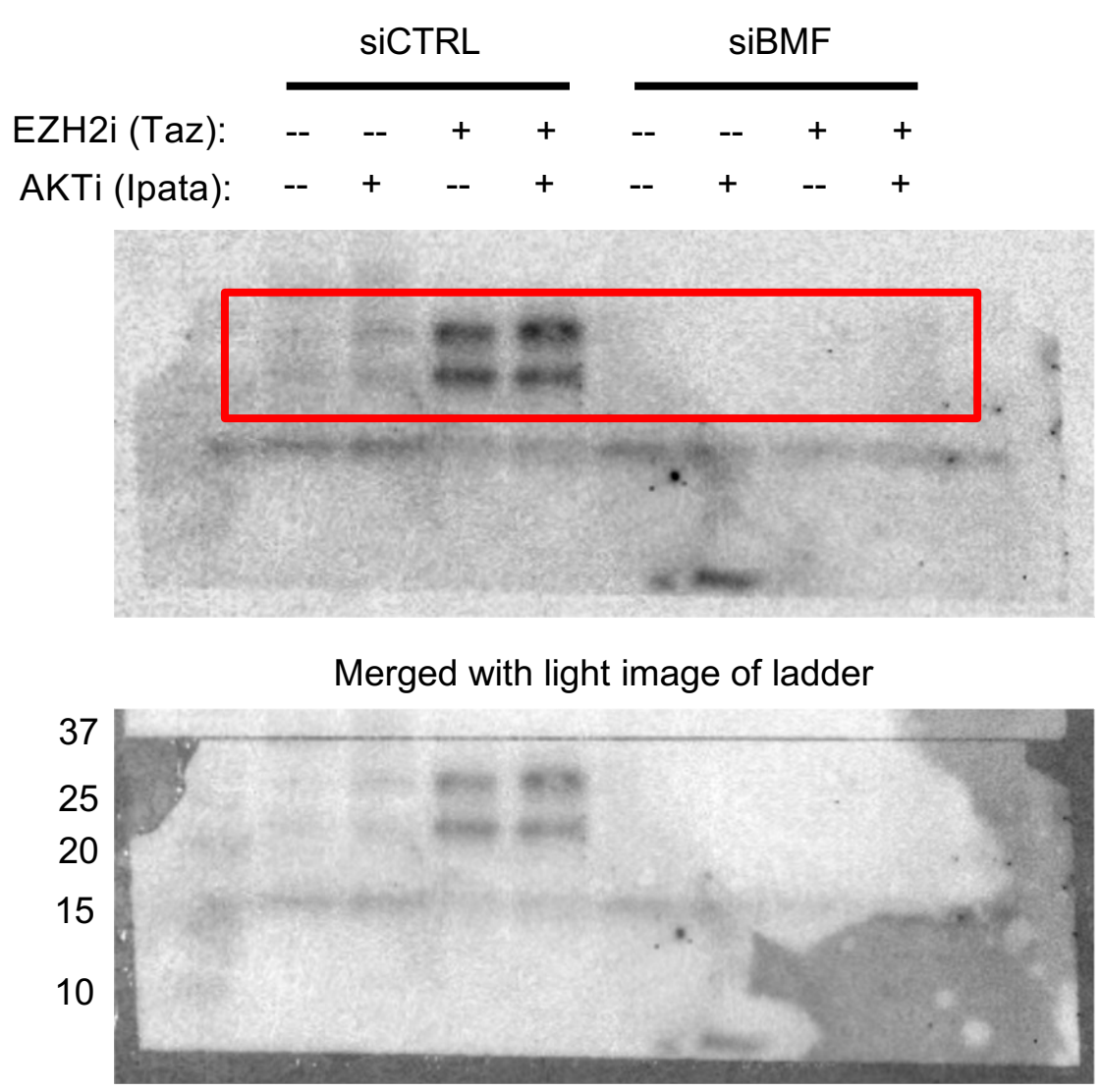

BMF

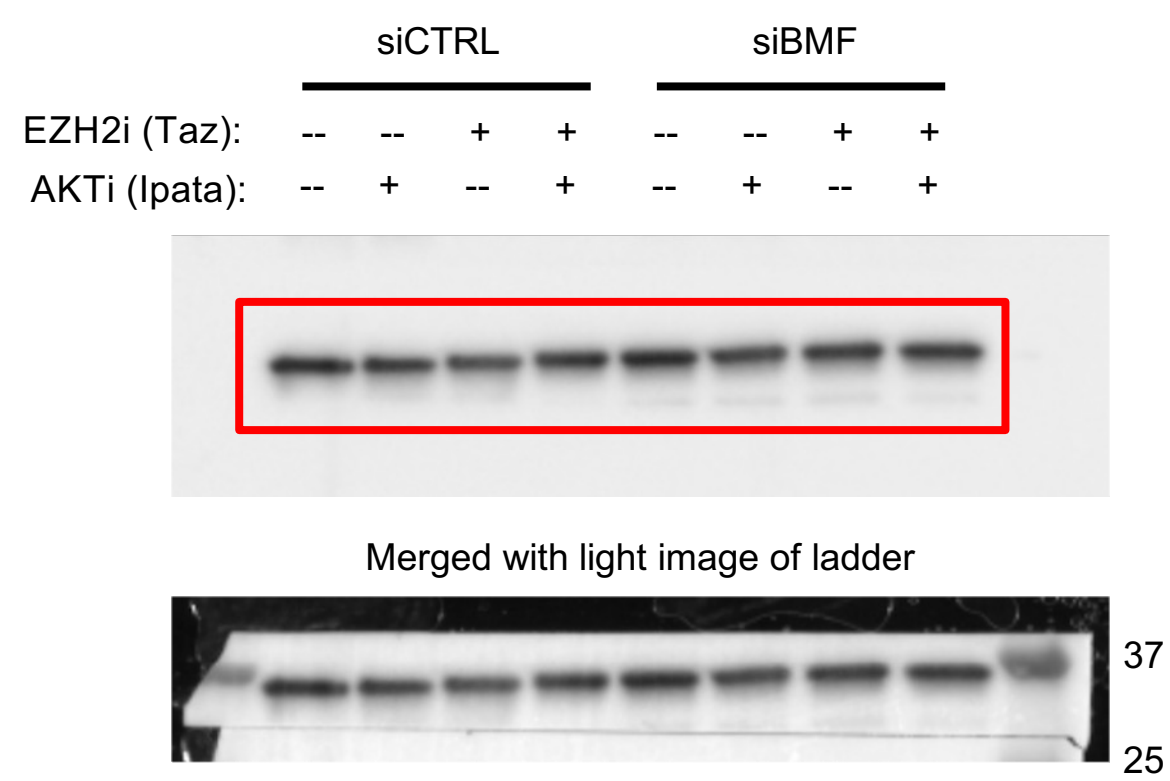

GAPDH

Raw unedited blots for Figure 5H

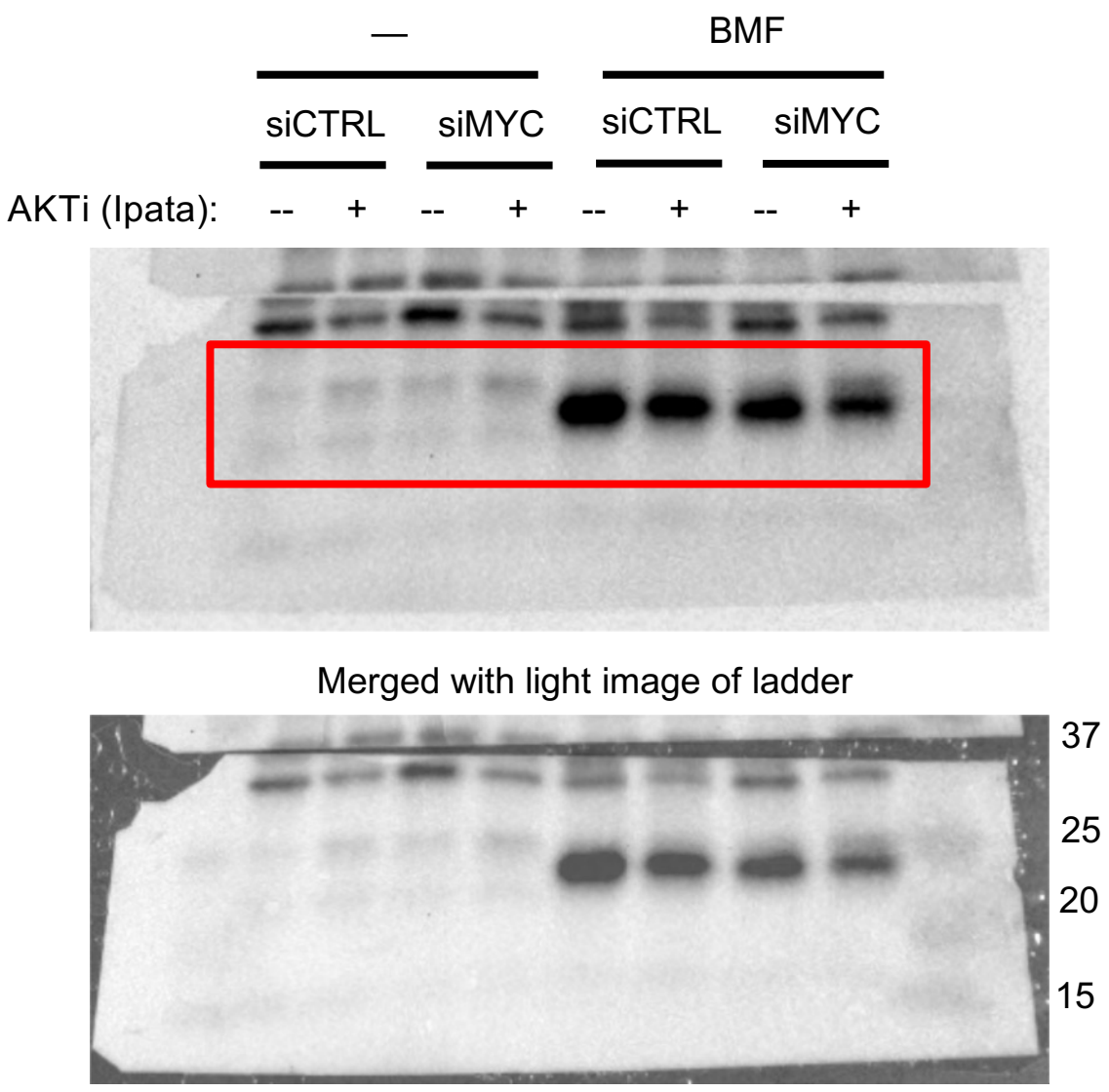

BMF

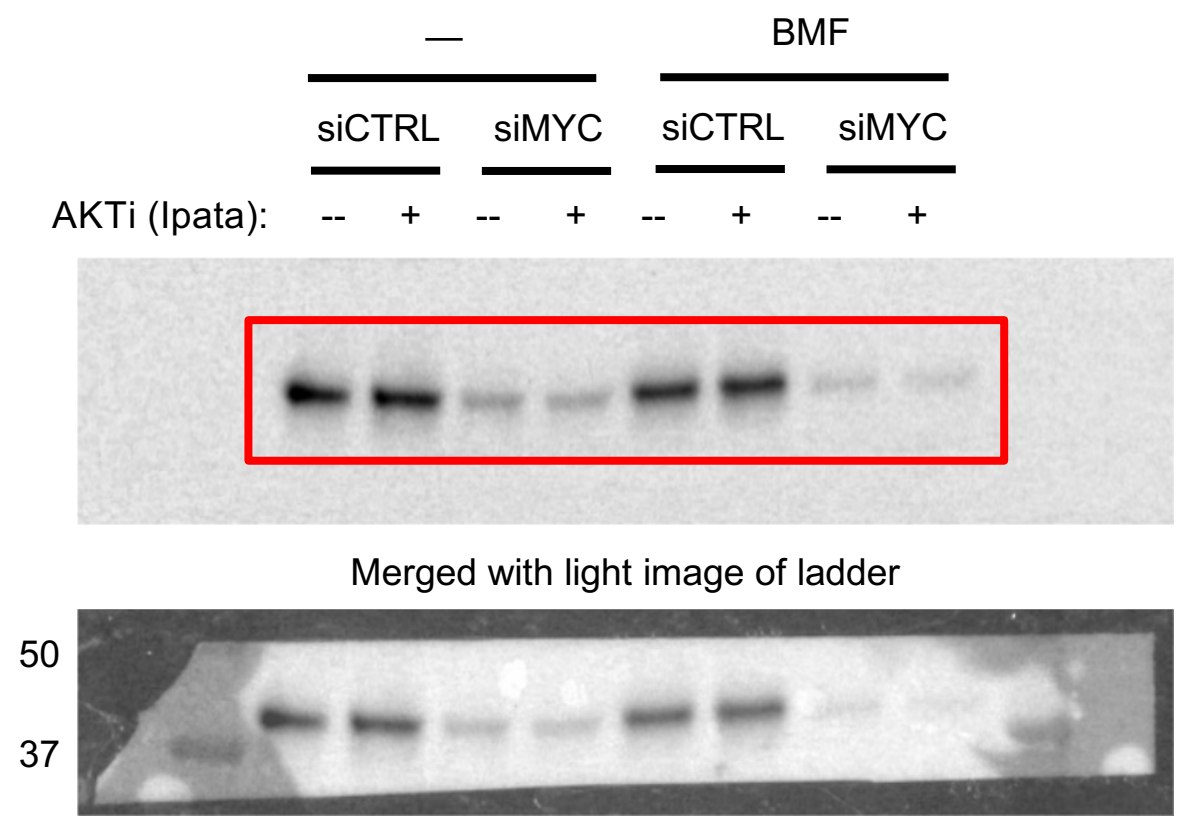

MYC

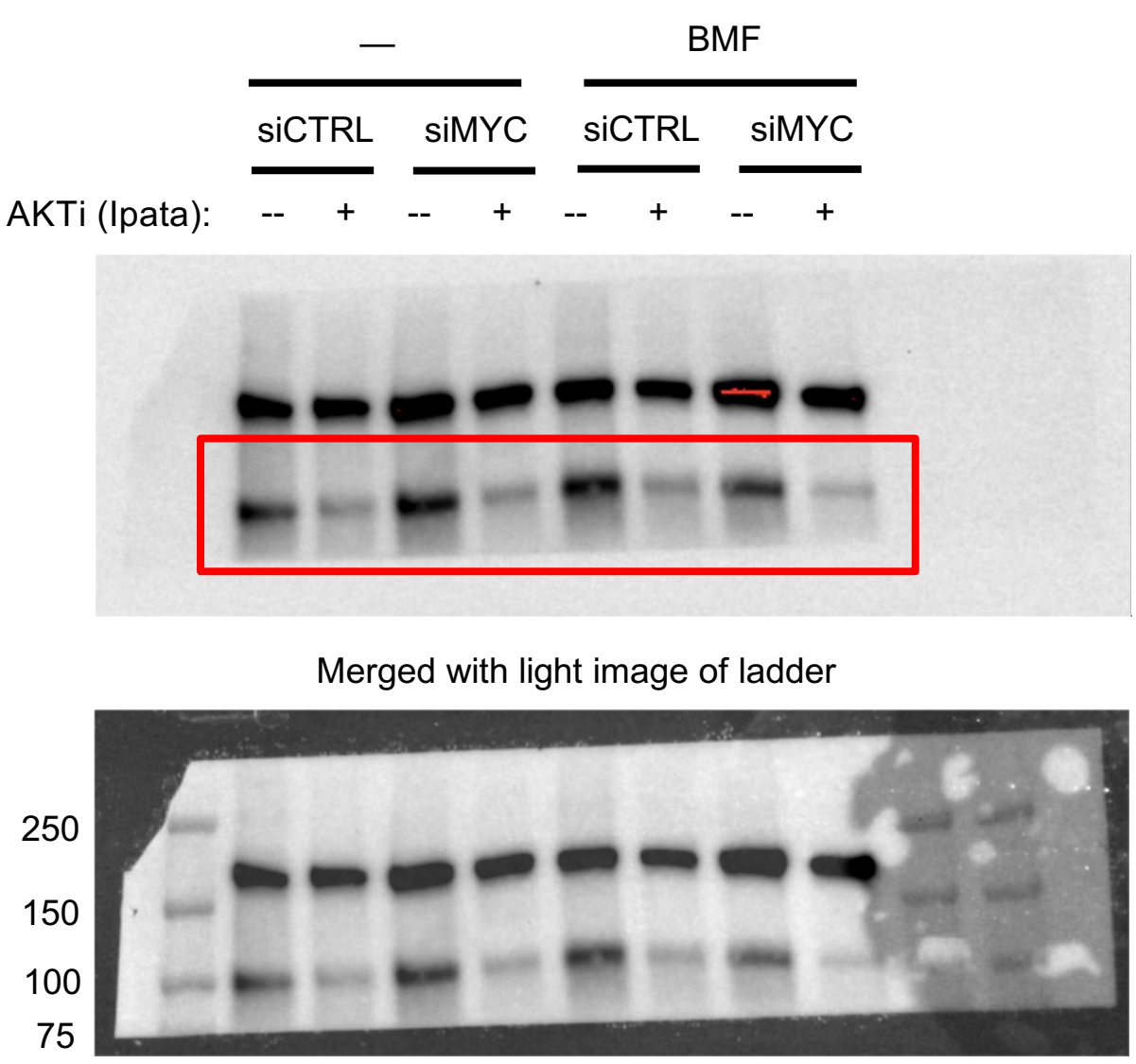

HIF-1A

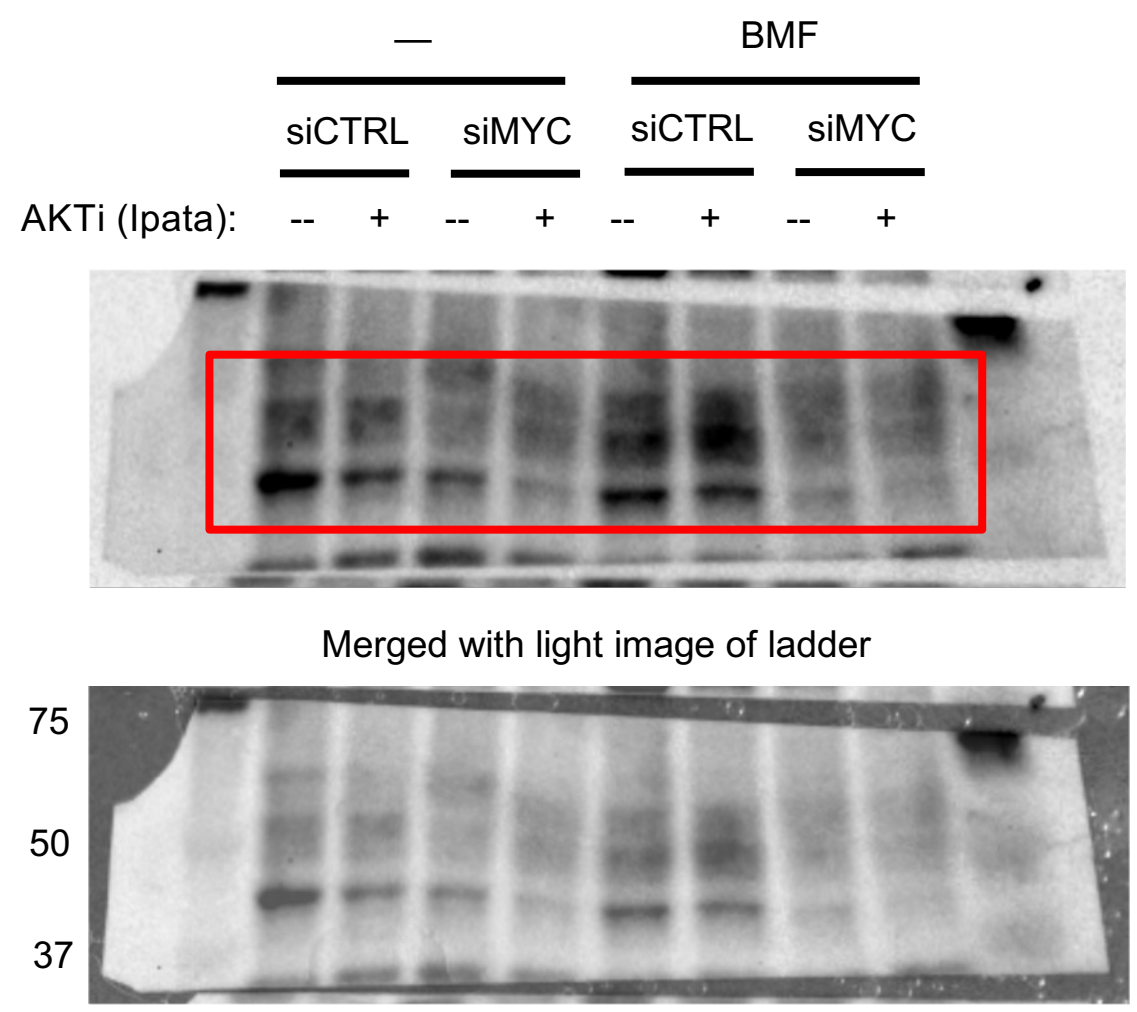

GLUT1

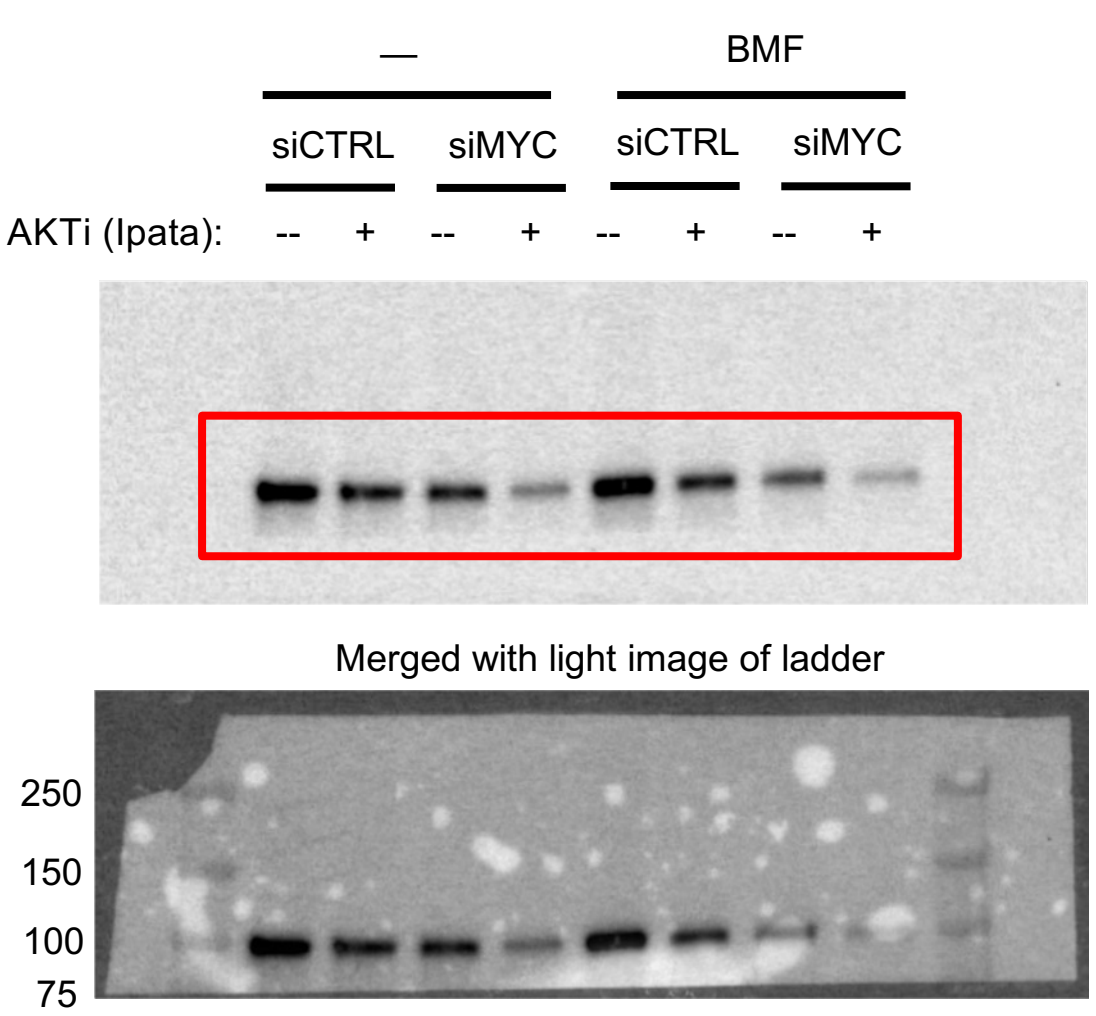

HK2

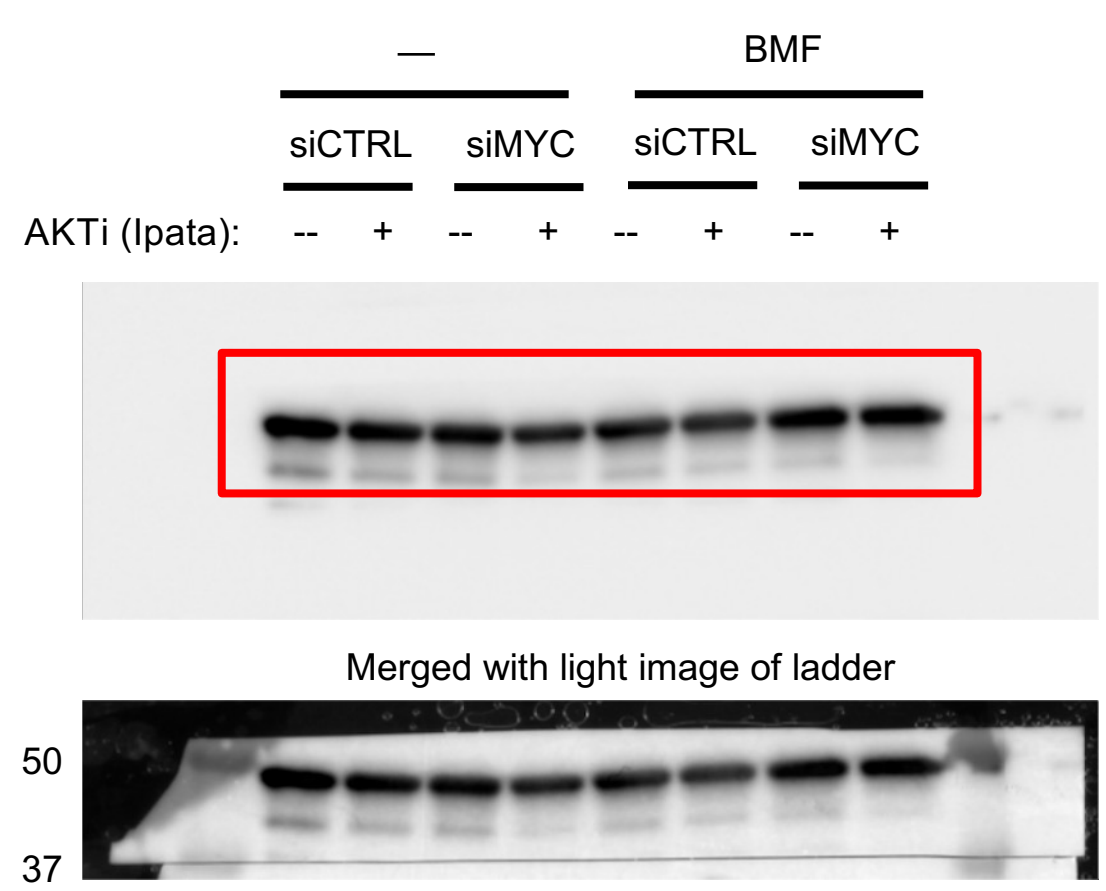

GAPDH

Raw unedited blots for Figure 5J

LuCaP 70-CR PDX

Note: same experiment and same GAPDH membrane as Figure 2F and 3N

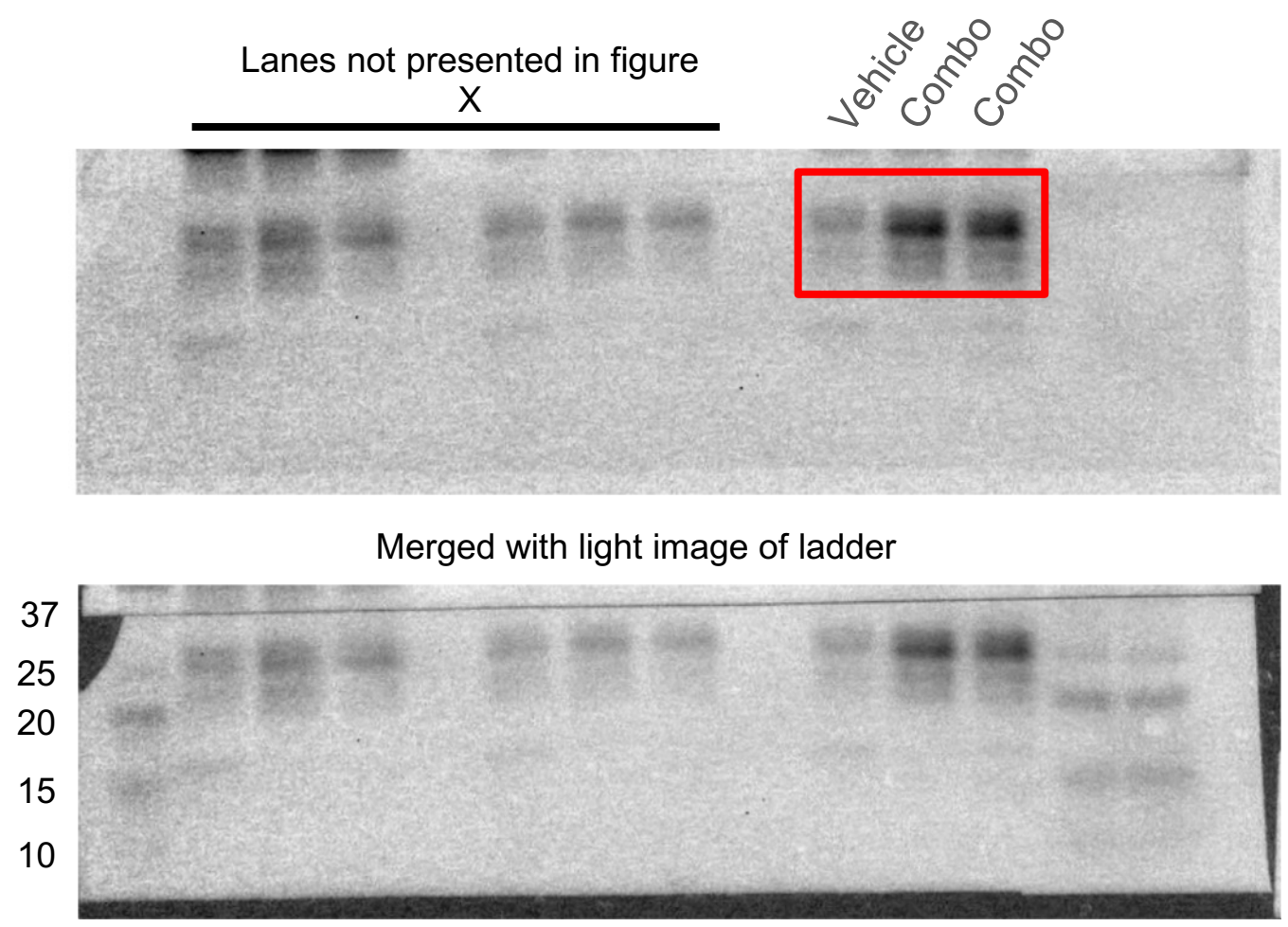

BMF

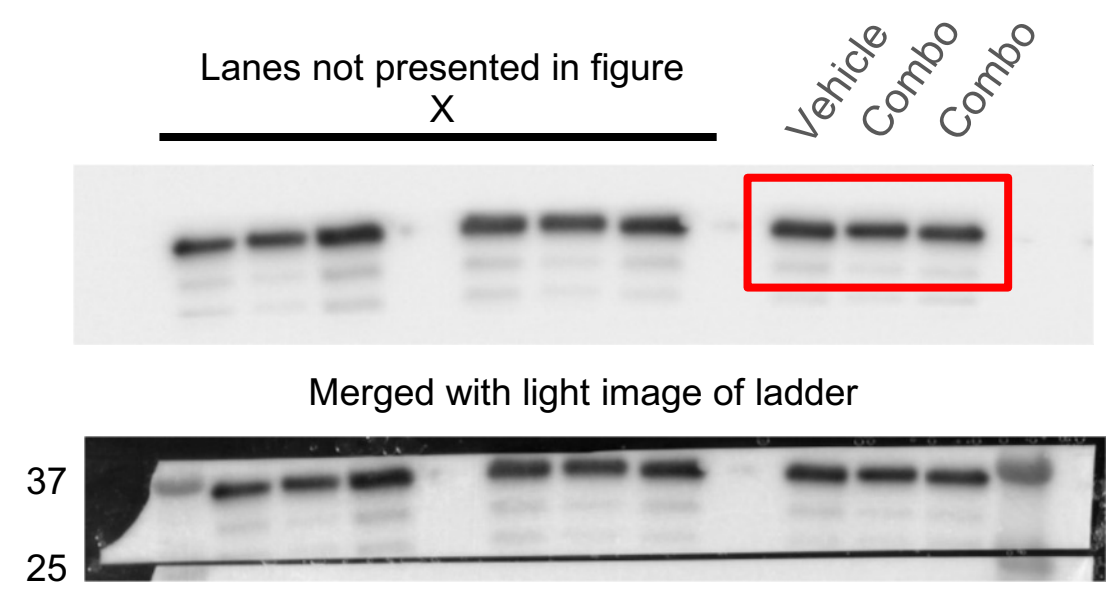

GAPDH

Raw unedited blots for Figure 5K

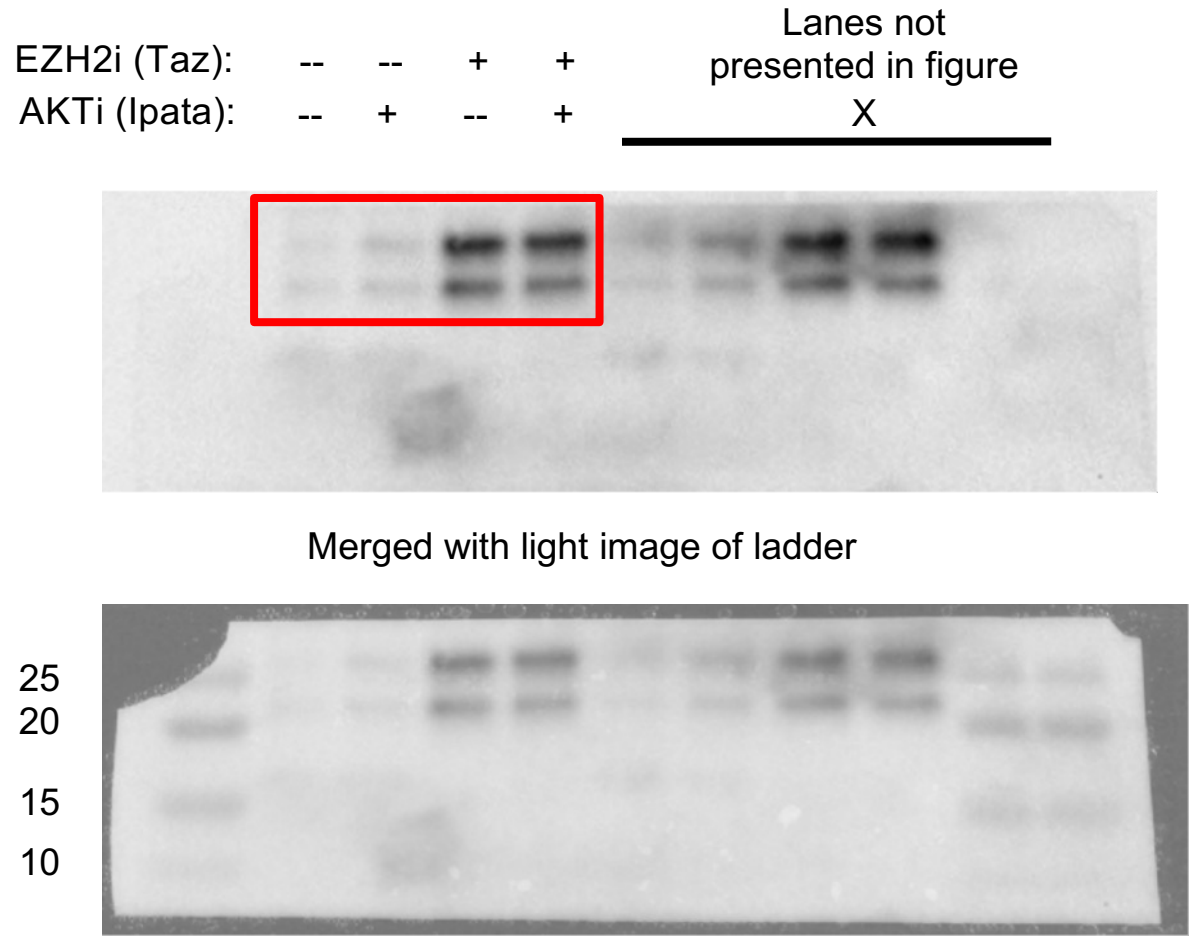

BMF

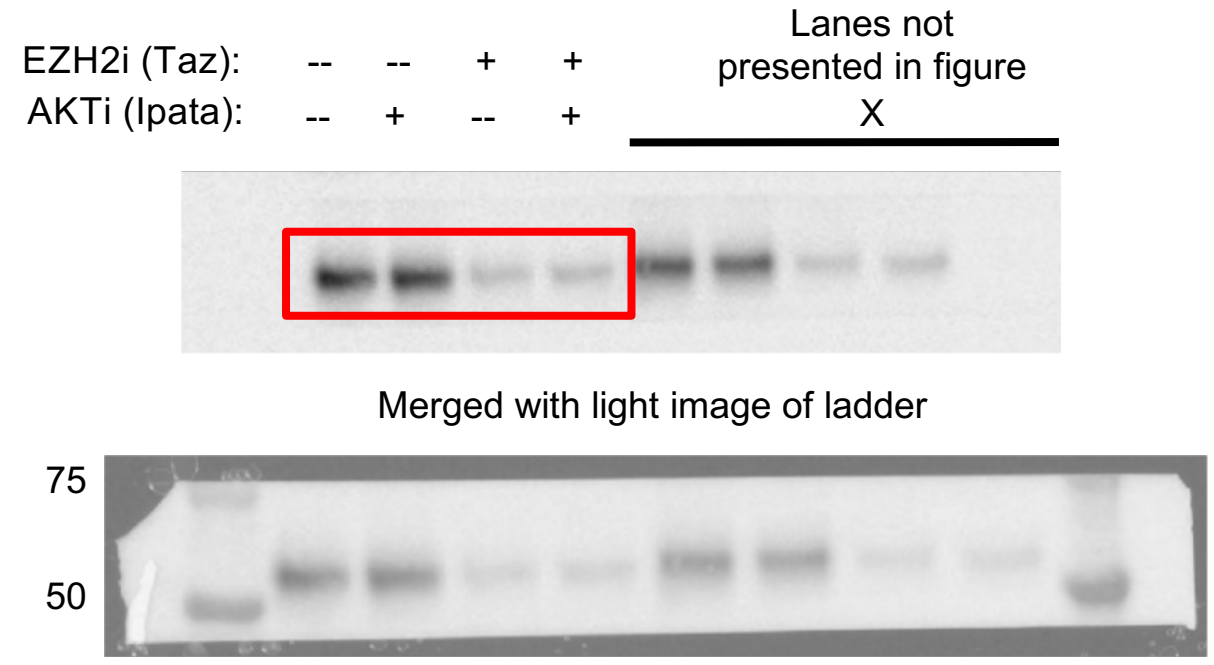

MYC

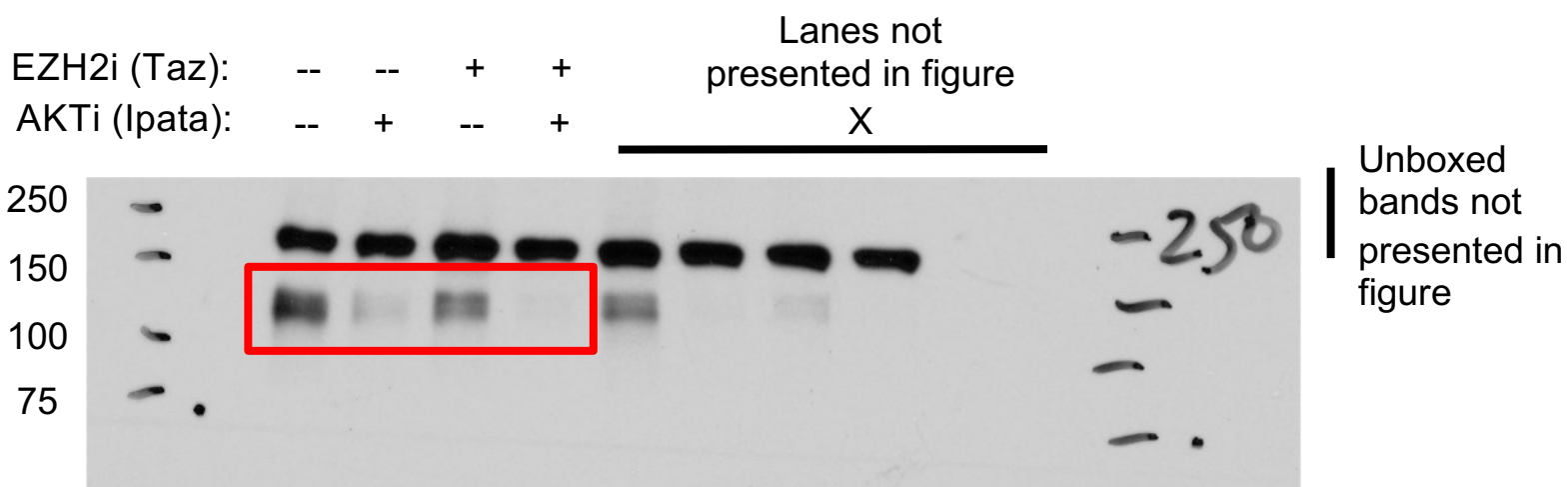

HIF-1A

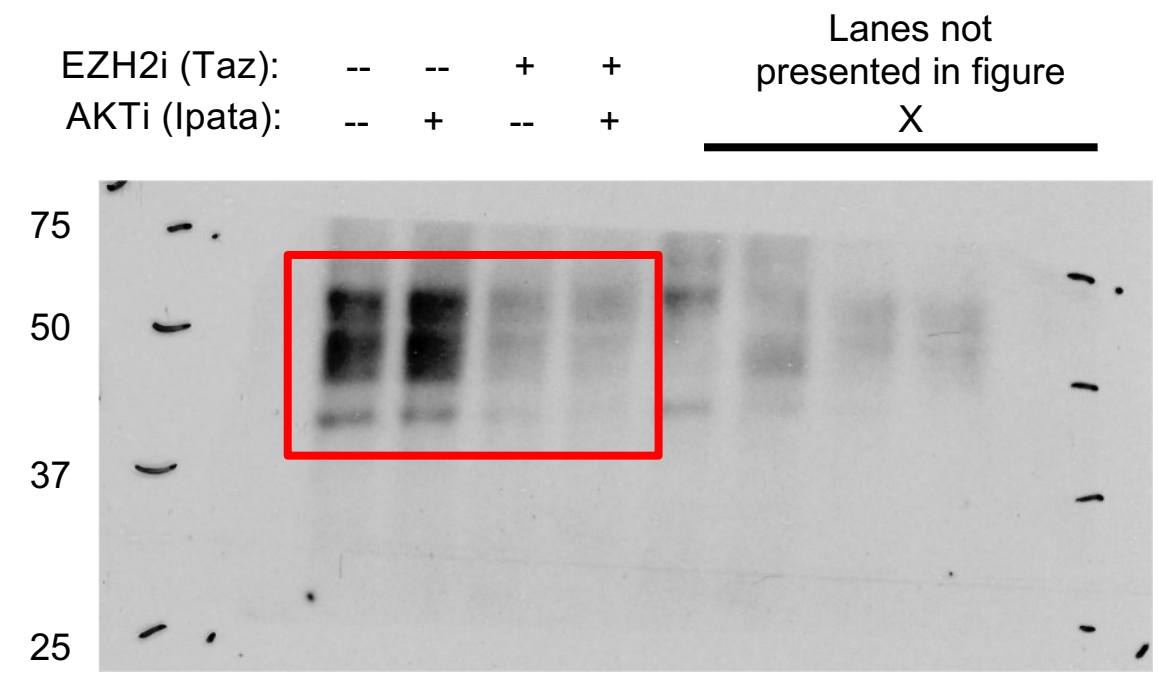

GLUT1

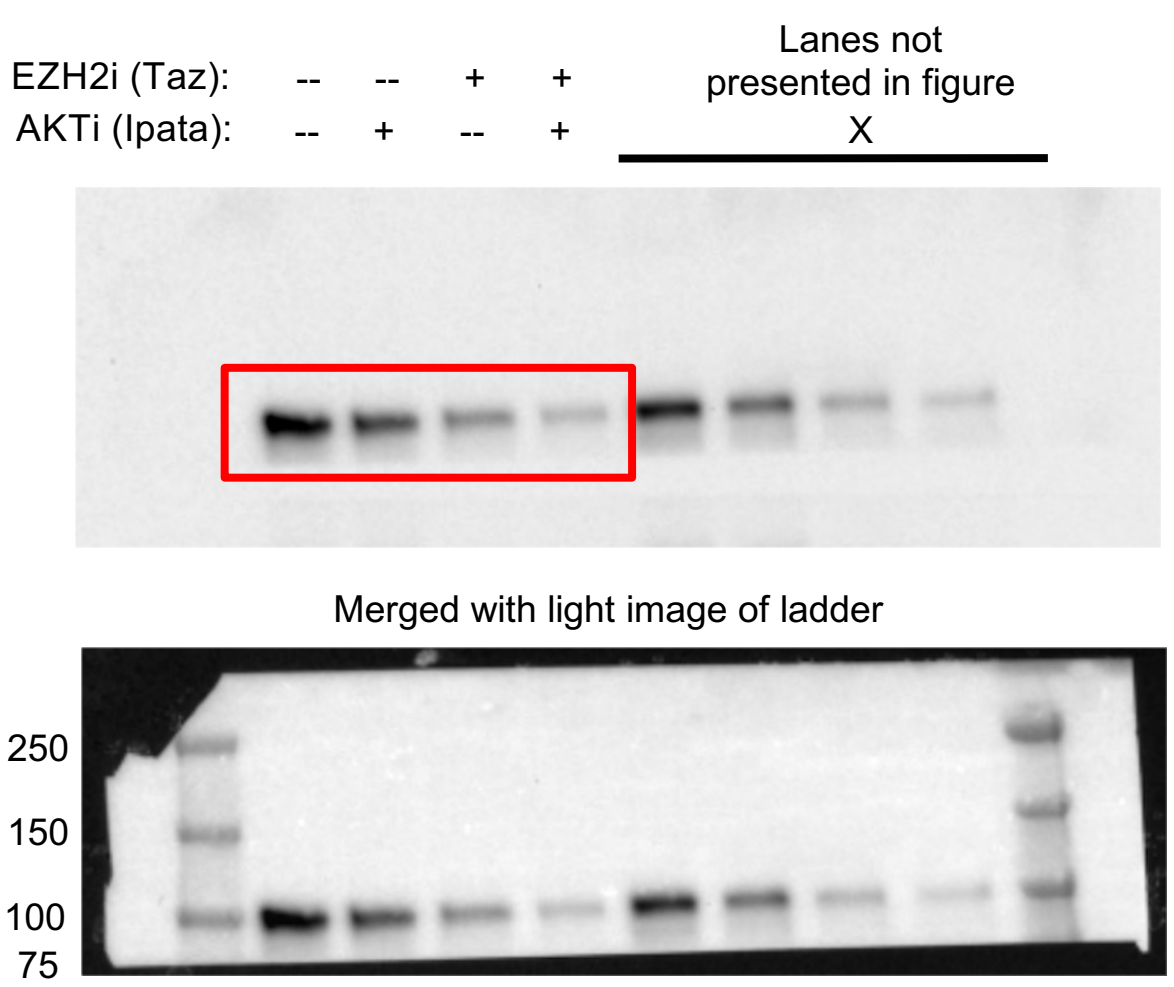

HK2

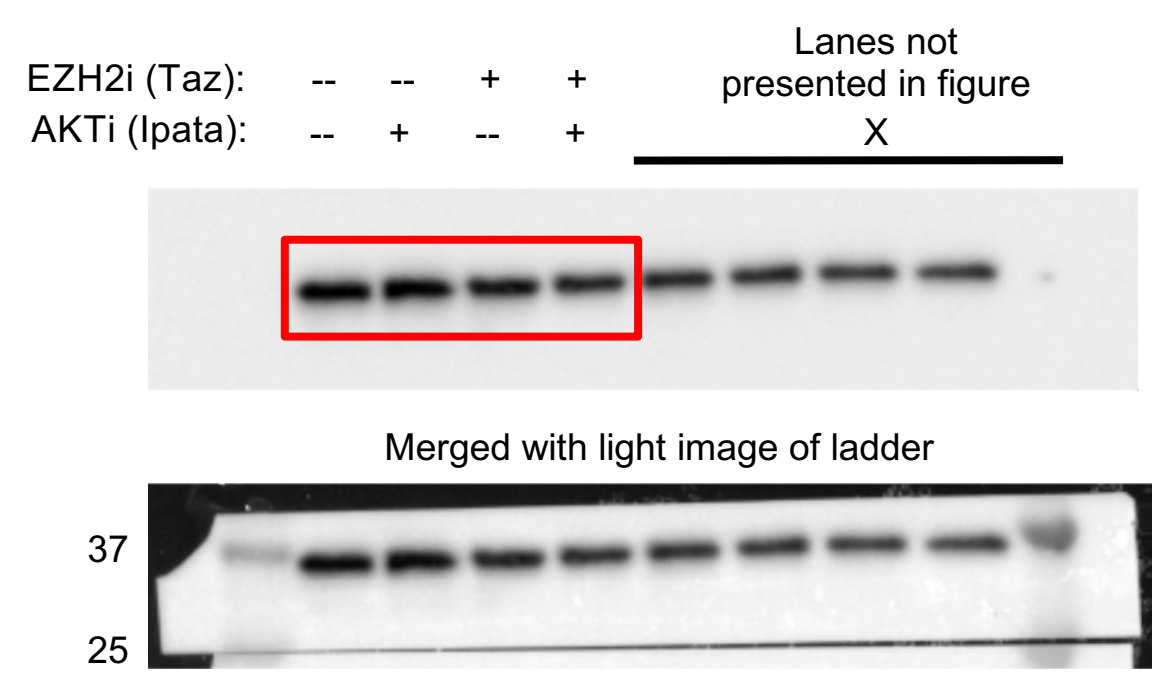

GAPDH

Raw unedited blots for Supplemental Figure 1B

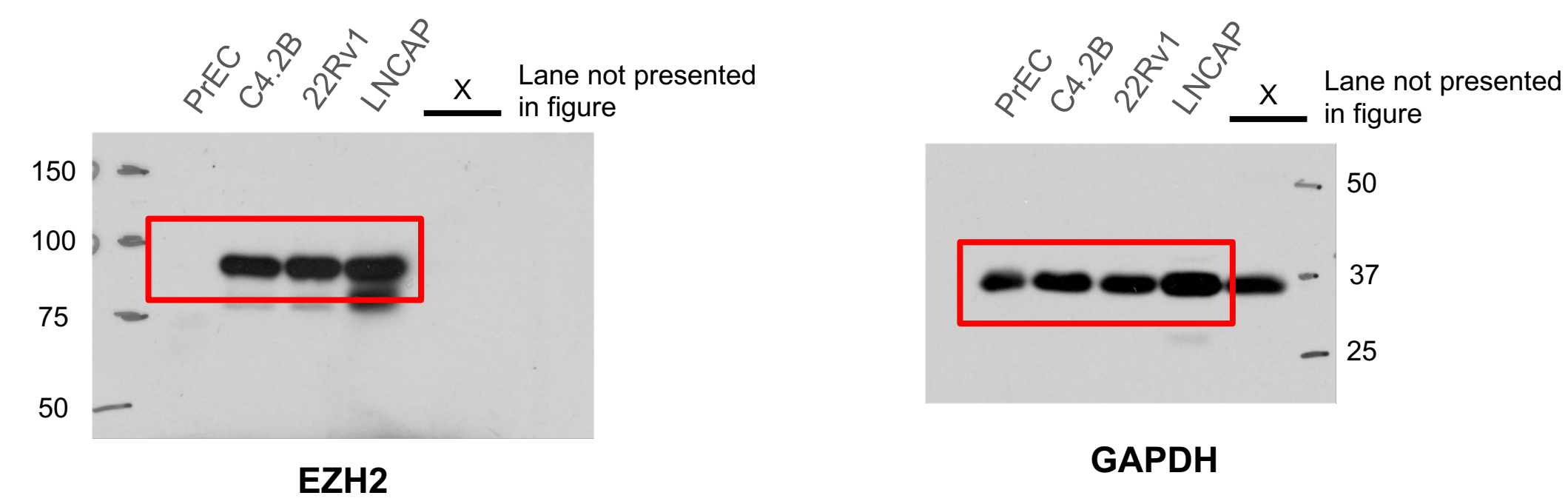

Raw unedited blots for Supplemental Figure 1D

PC3

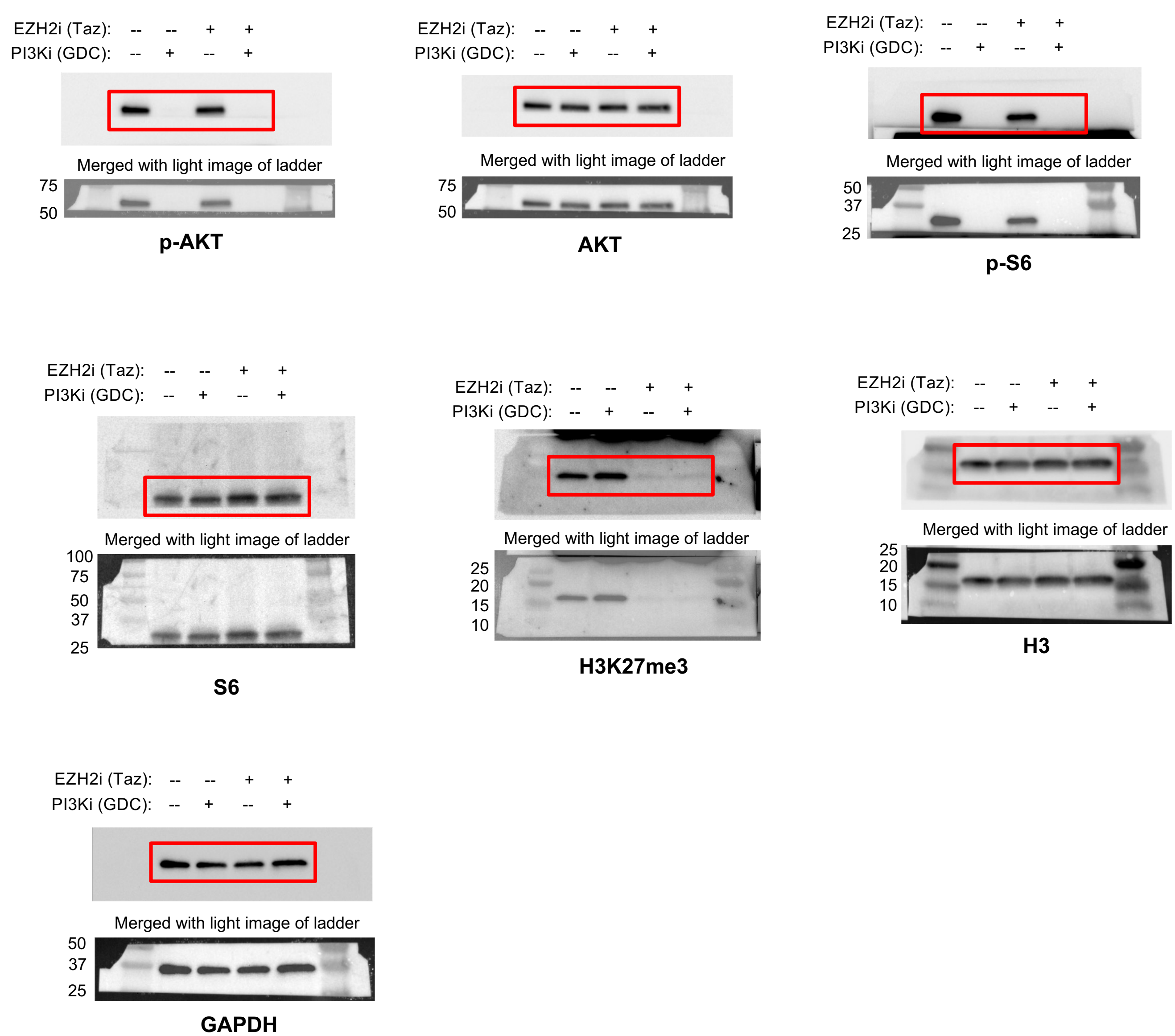

Raw unedited blots for Supplemental Figure 1D

PC3

EZH2i (Taz): -- -- + +
AKTi (Ipata): -- + -- +

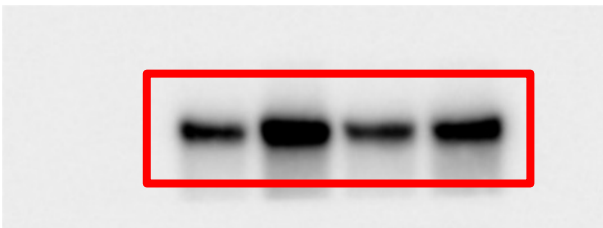

Merged with light image of ladder

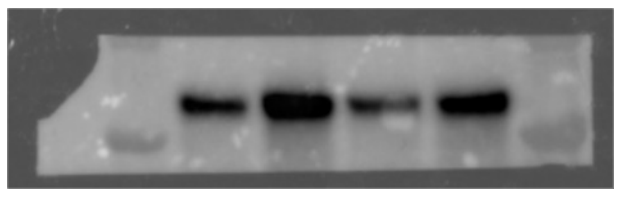

p-AKT

EZH2i (Taz): -- -- + +
AKTi (Ipata): -- + -- +

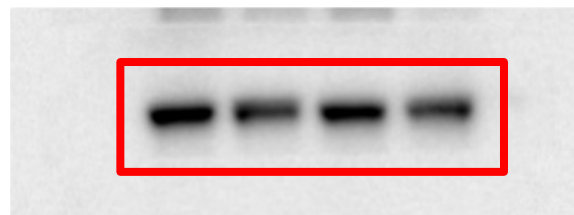

Merged with light image of ladder

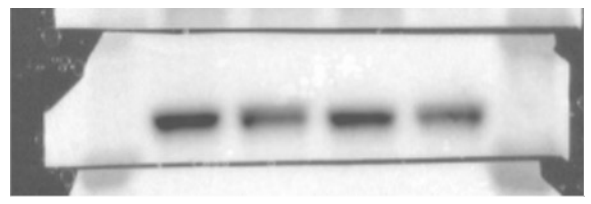

AKT

EZH2i (Taz): -- -- + +
AKTi (Ipata): -- + -- +

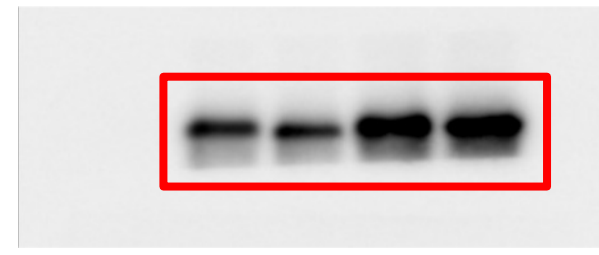

Merged with light image of ladder

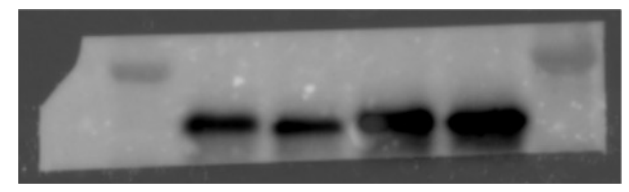

S6

EZH2i (Taz): -- -- + +
AKTi (Ipata): -- + -- +

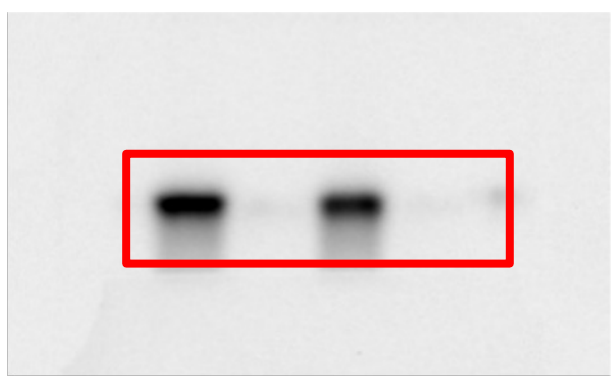

Merged with light image of ladder

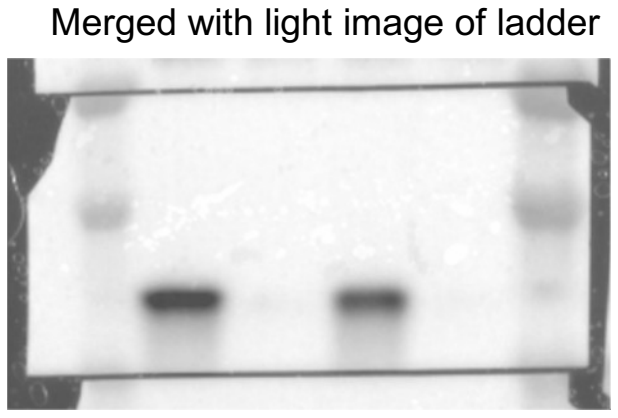

p-S6

EZH2i (Taz): -- -- + +
AKTi (Ipata): -- + -- +

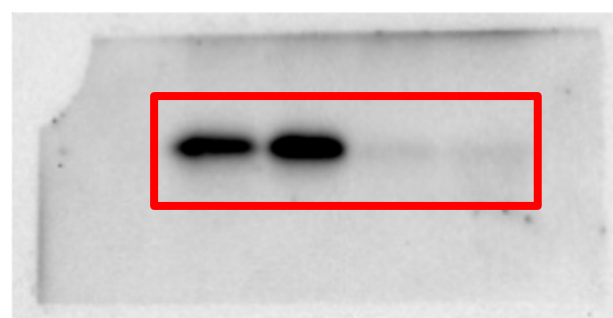

Merged with light image of ladder

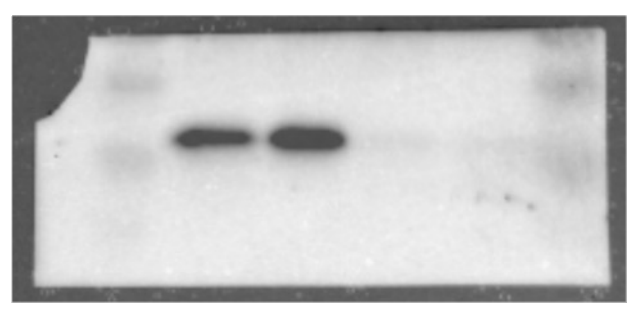

H3K27me3

EZH2i (Taz): -- -- + +
AKTi (Ipata): -- + -- +

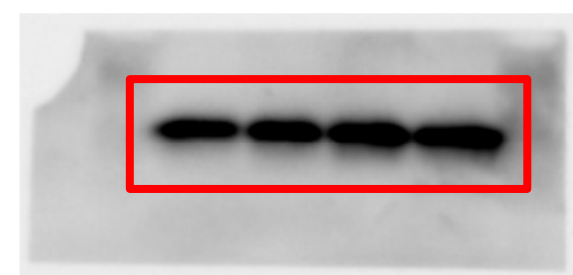

Merged with light image of ladder

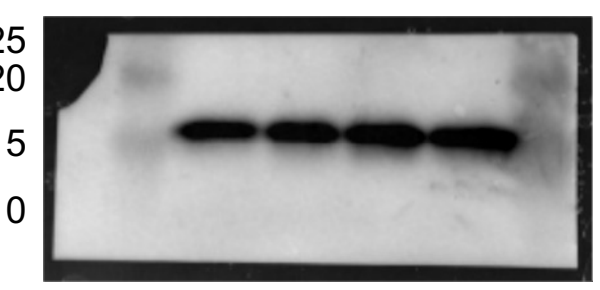

H3

EZH2i (Taz): -- -- + +
AKTi (Ipata): -- + -- +

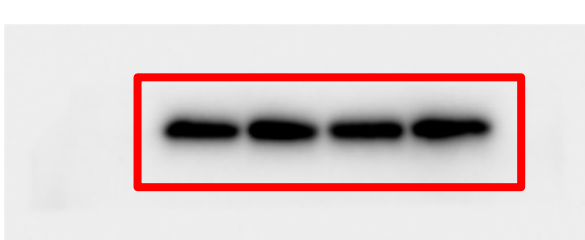

Merged with light image of ladder

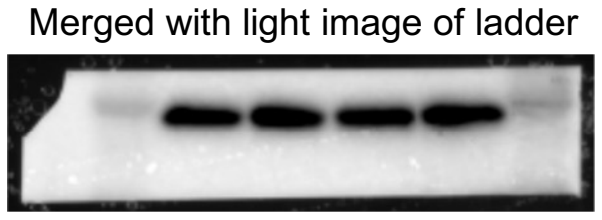

GAPDH

Raw unedited blots for Supplemental Figure 1D

PC3

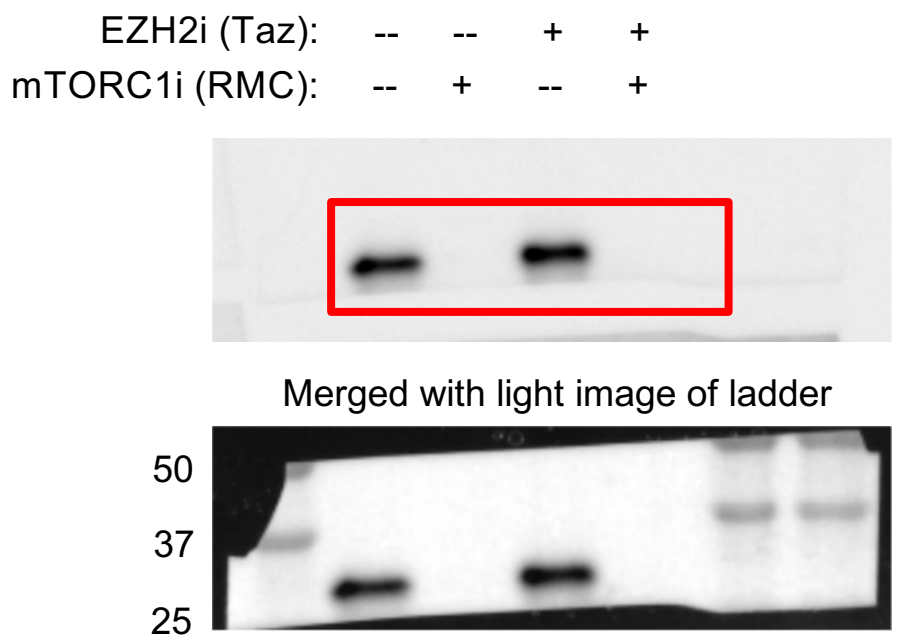

p-S6

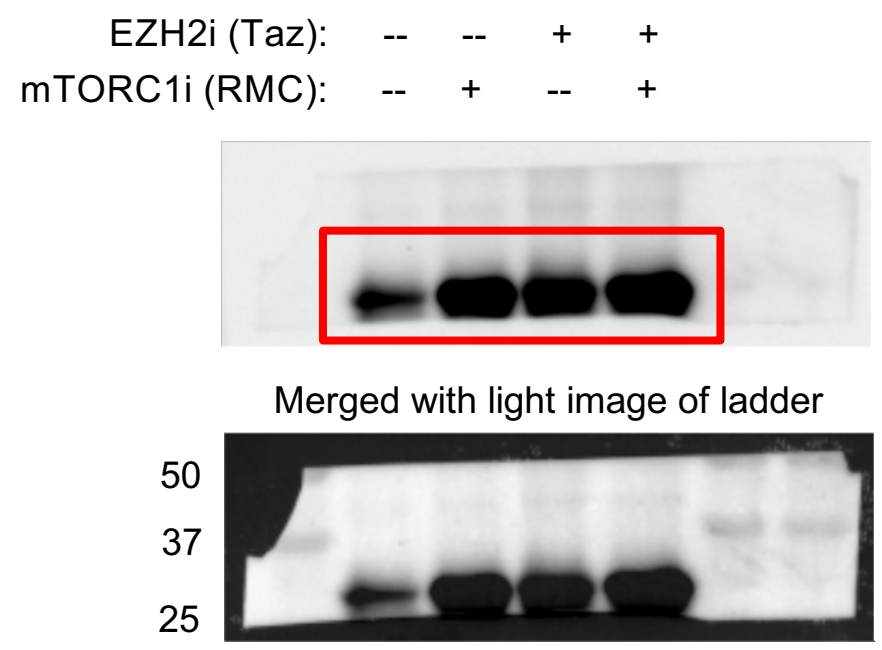

S6

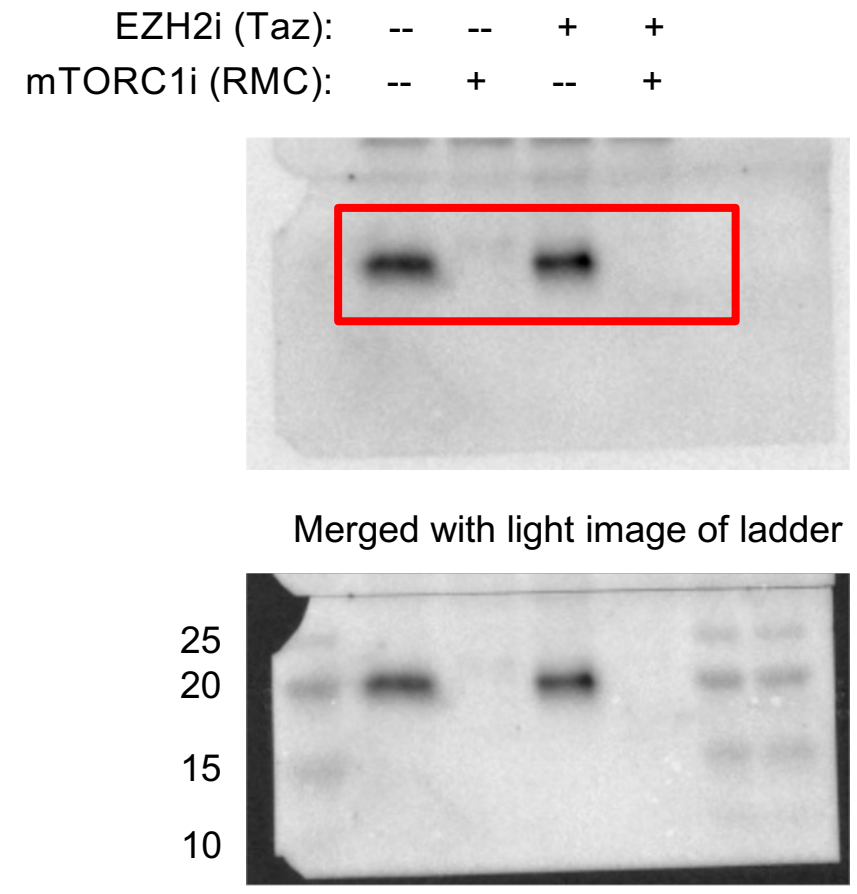

p-4EBP1

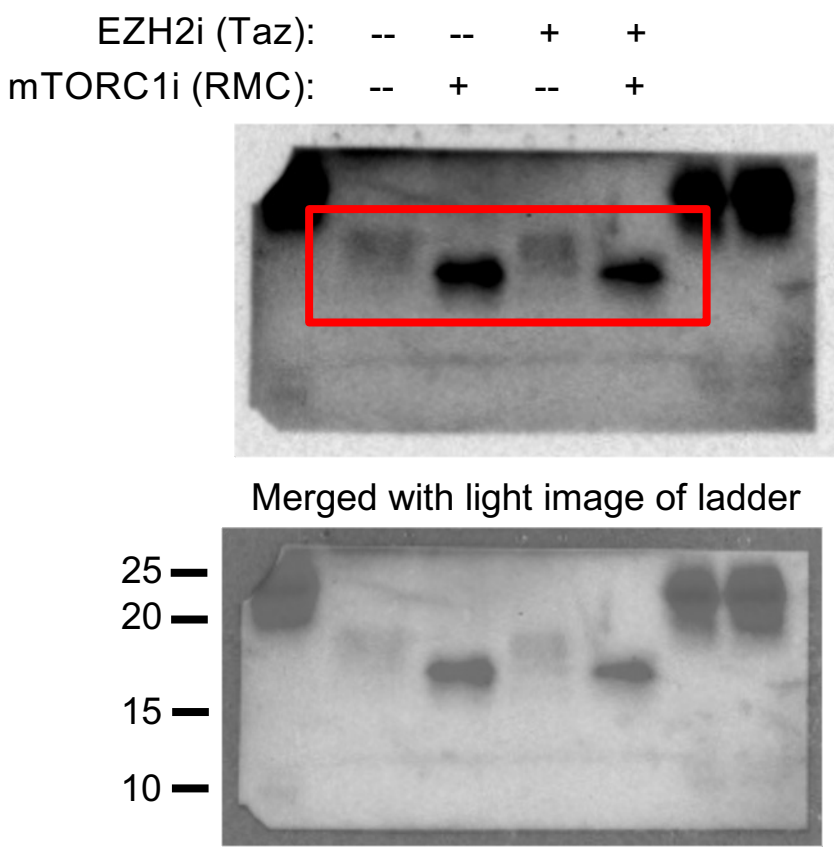

4EBP1

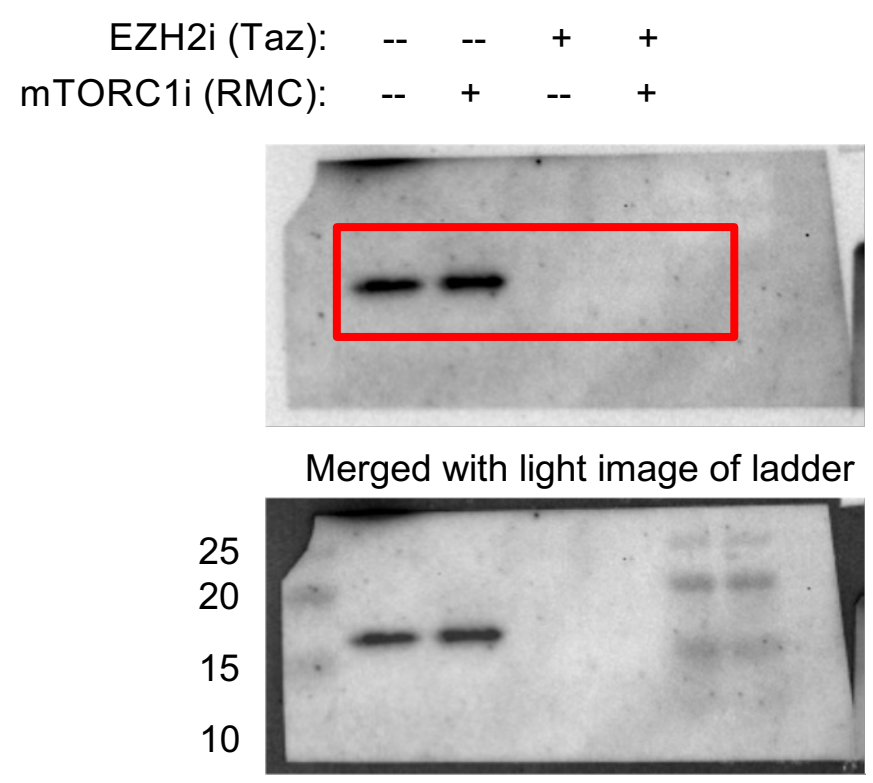

H3K27me3

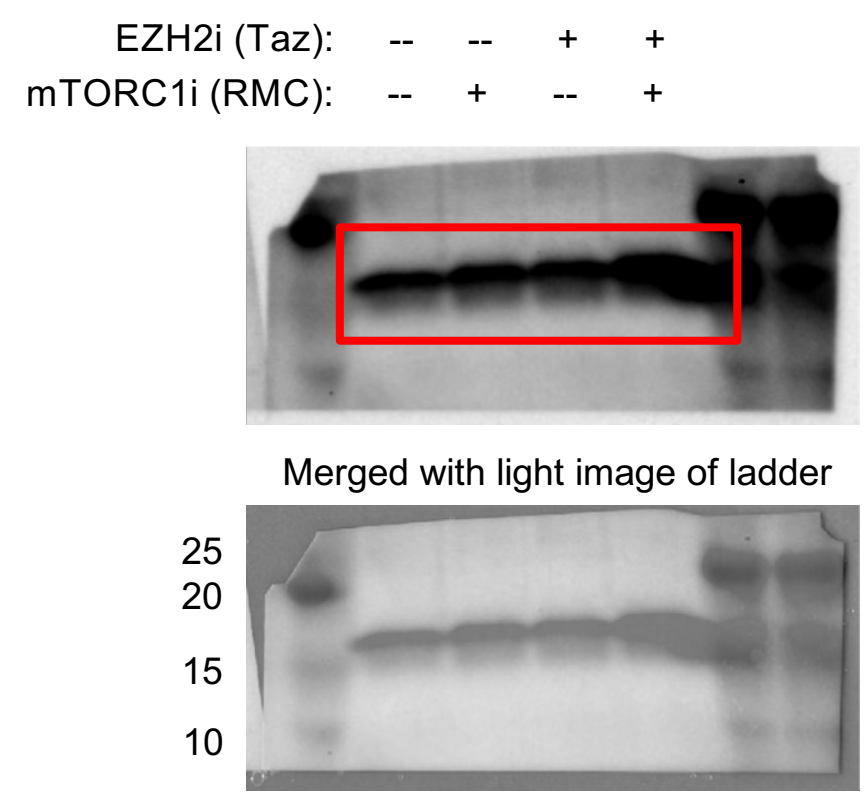

H3

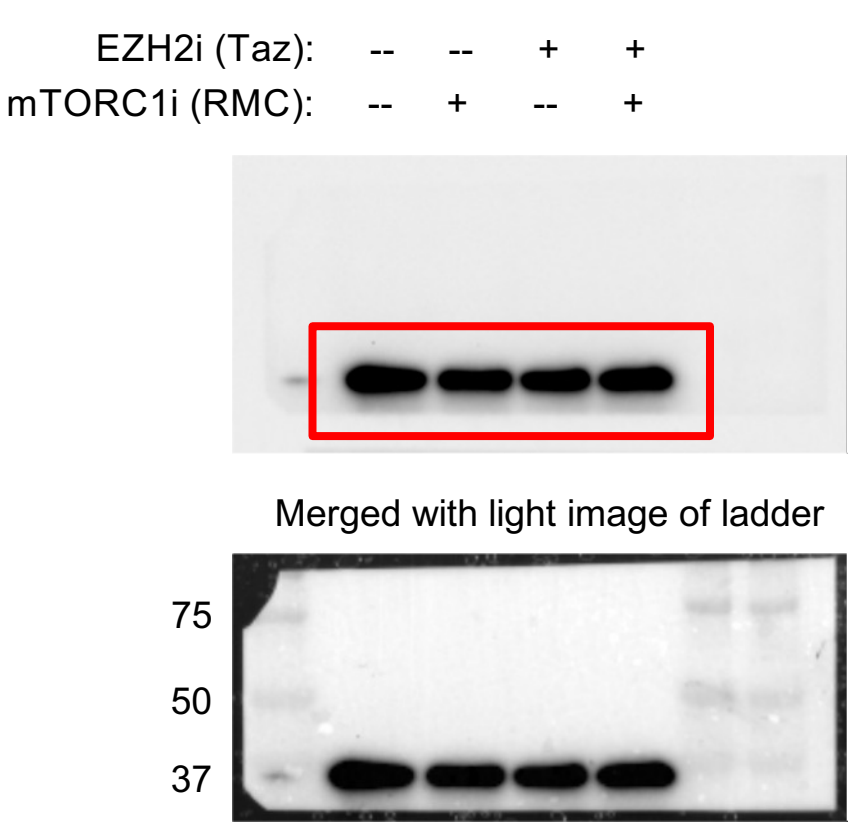

GAPDH

Raw unedited blots for Supplemental Figure 1E

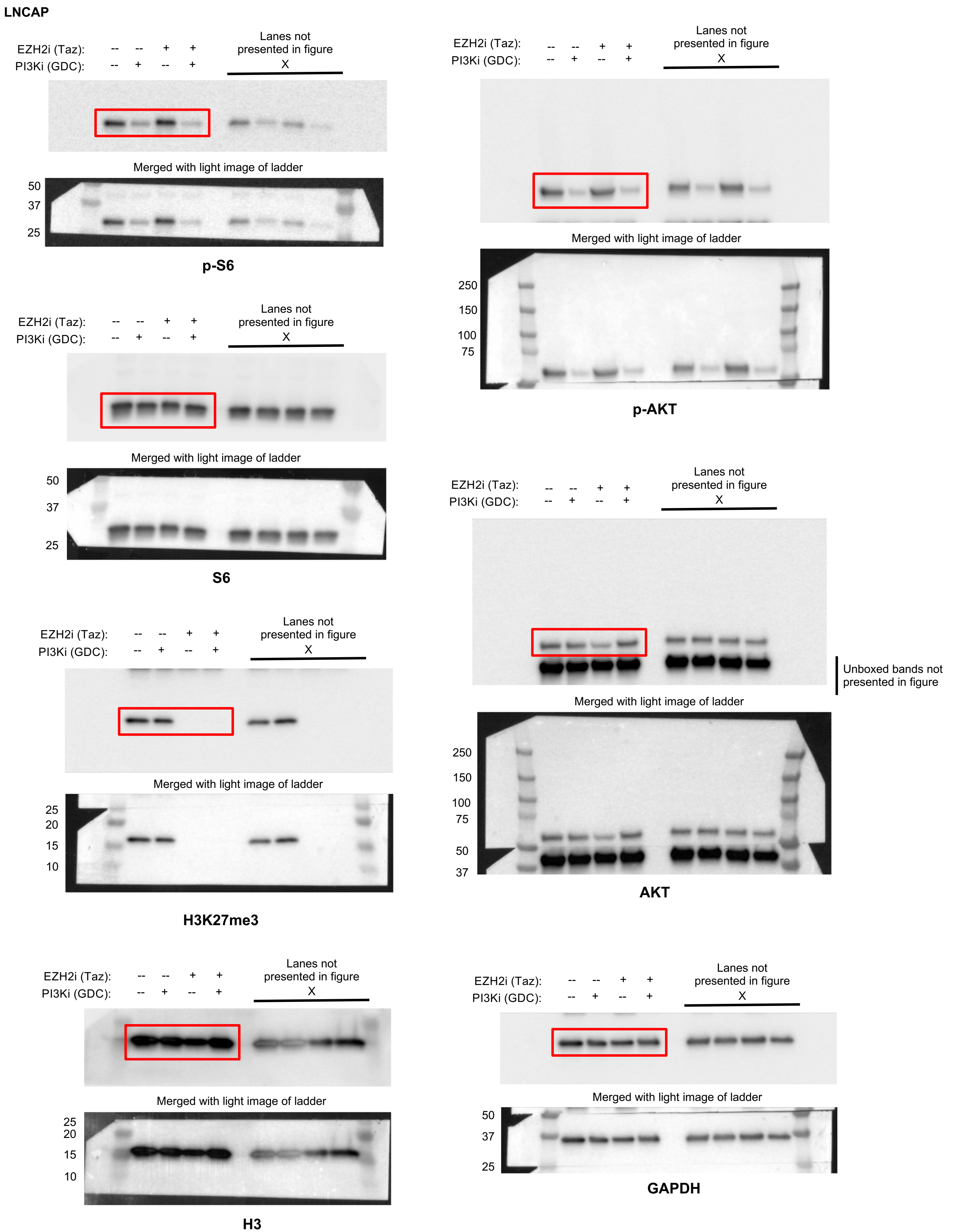

Raw unedited blots for Supplemental Figure 1E

LNCAP

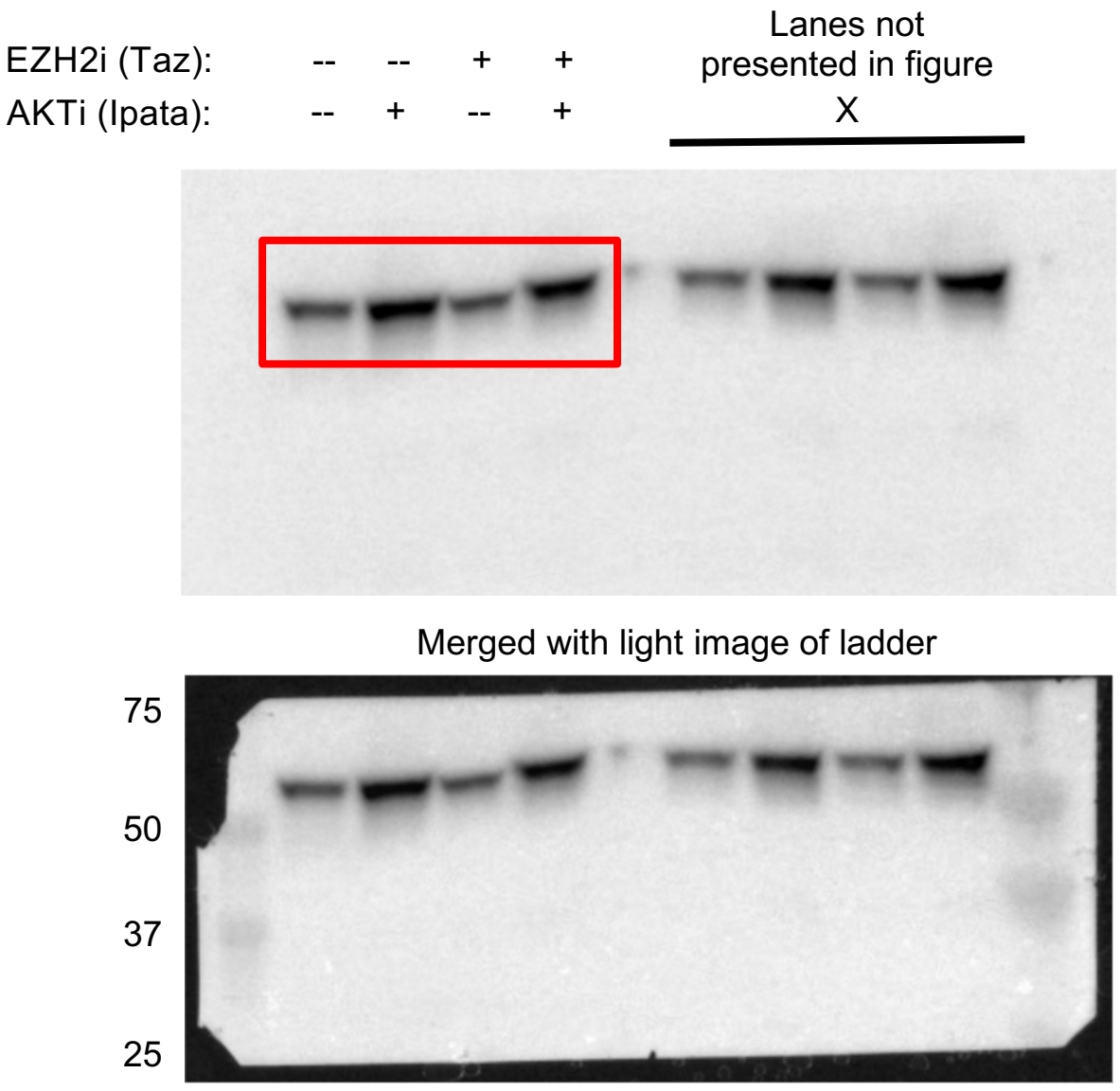

p-AKT

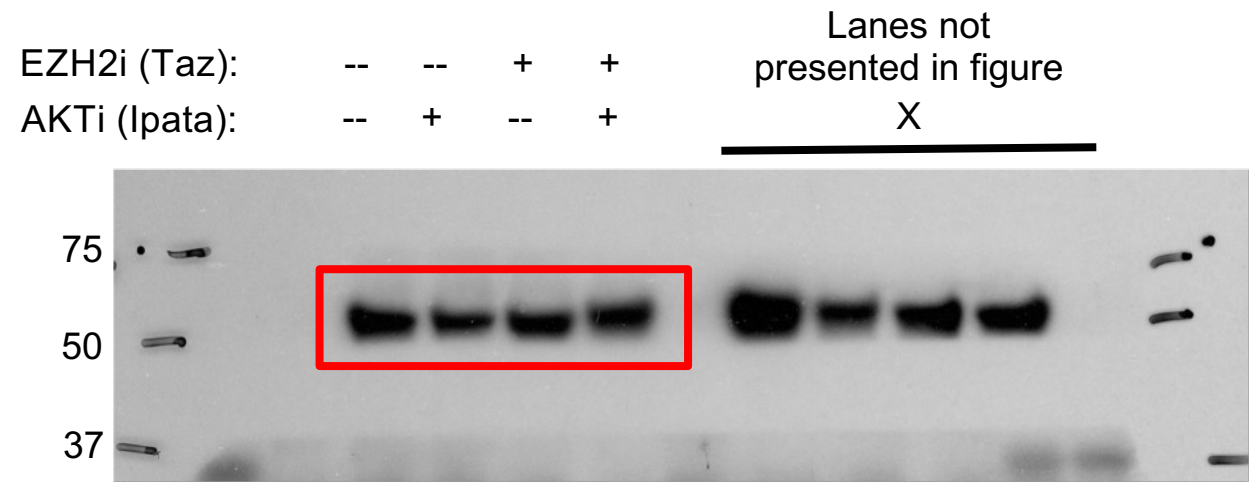

AKT

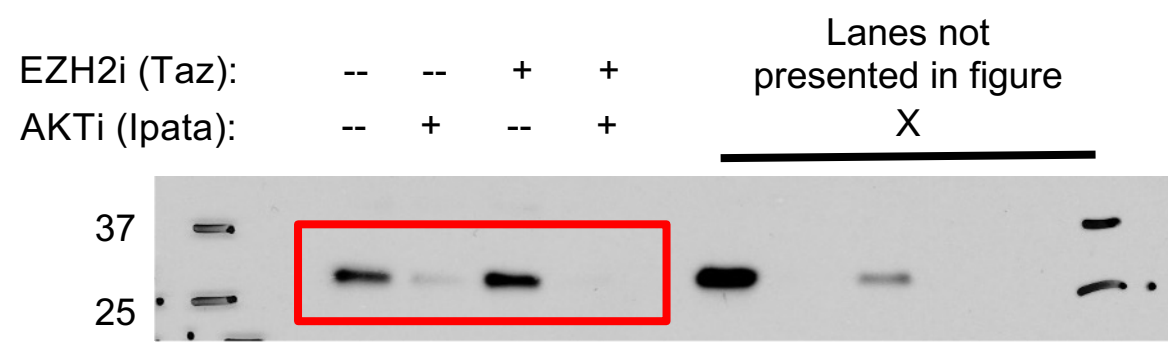

p-S6

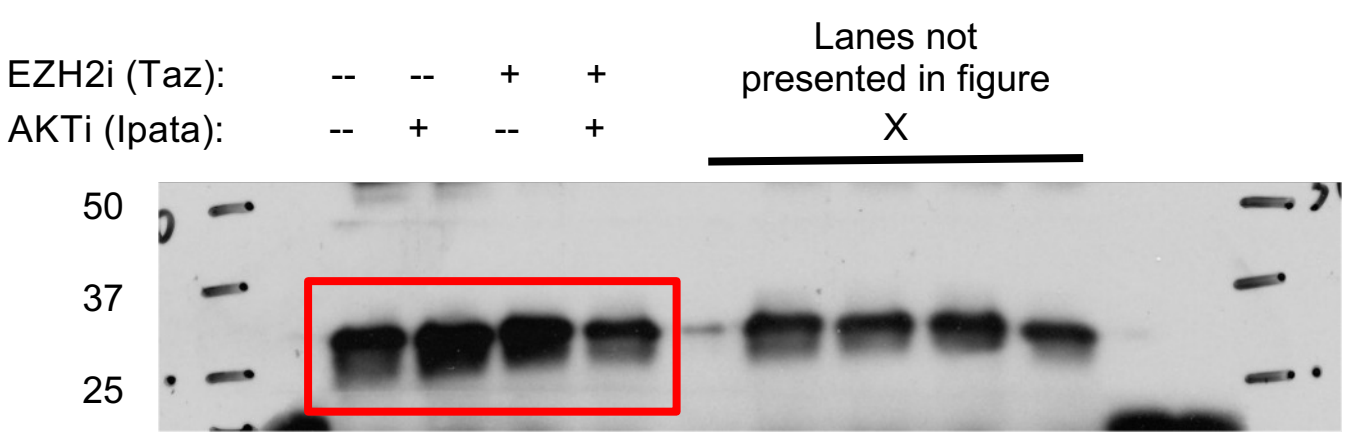

S6

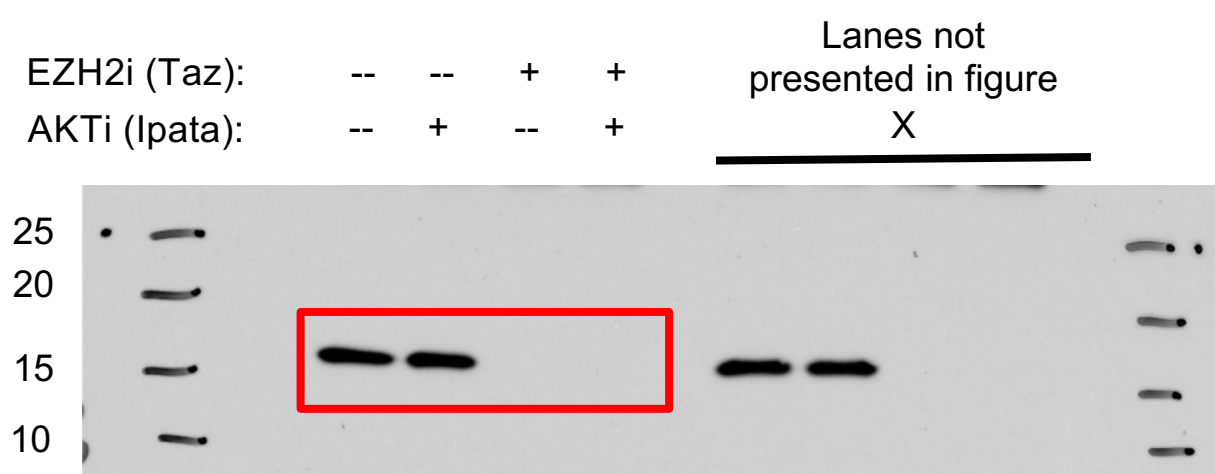

H3K27me3

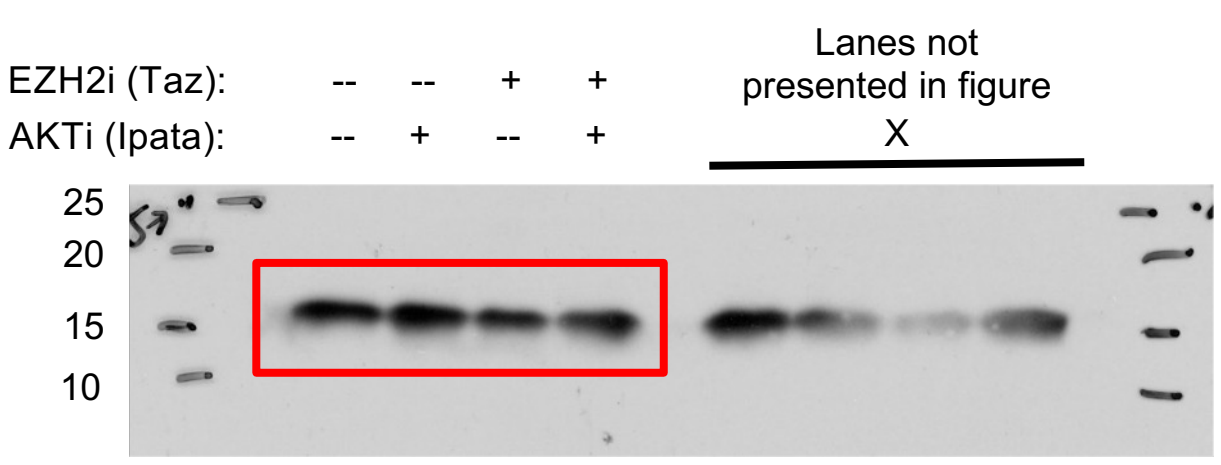

H3

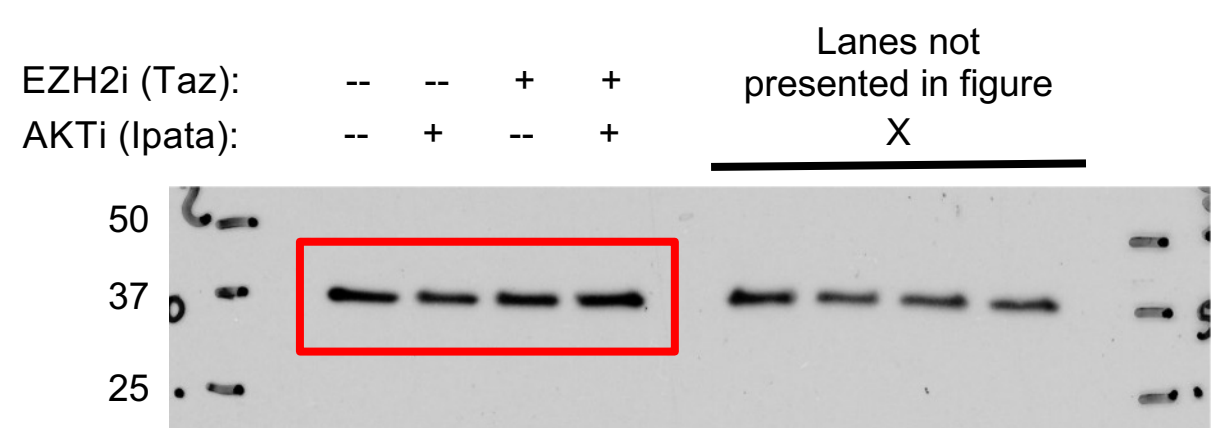

GAPDH

Raw unedited blots for Supplemental Figure 1E

LNCAP

EZH2i (Taz): -- -- + + | Lanes not presented in figure
mTORC1i (RMC): -- + -- + | X

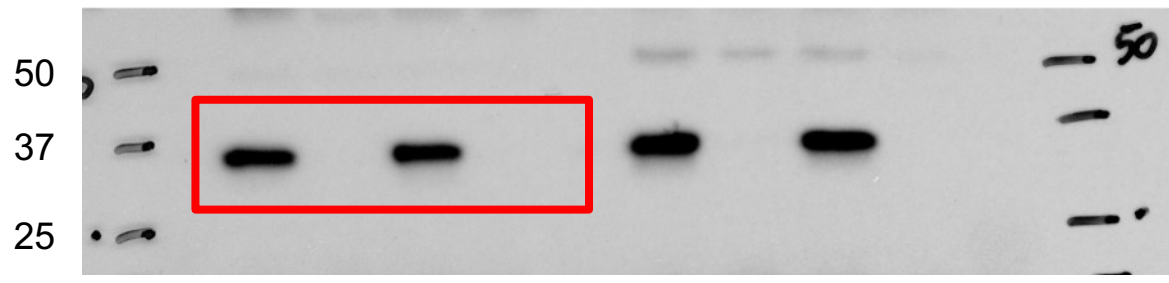

p-S6

EZH2i (Taz): -- -- + + | Lanes not presented in figure
mTORC1i (RMC): -- + -- + | X

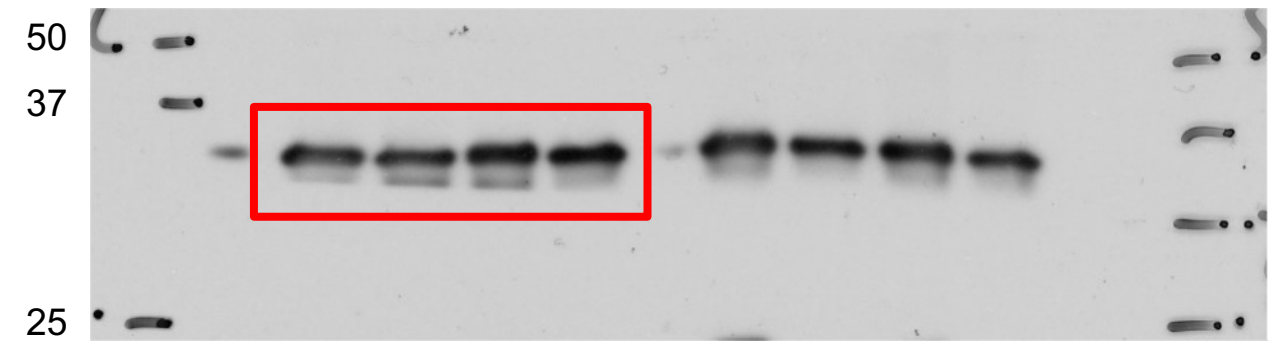

S6

EZH2i (Taz): -- -- + + | Lanes not presented in figure
mTORC1i (RMC): -- + -- + | X

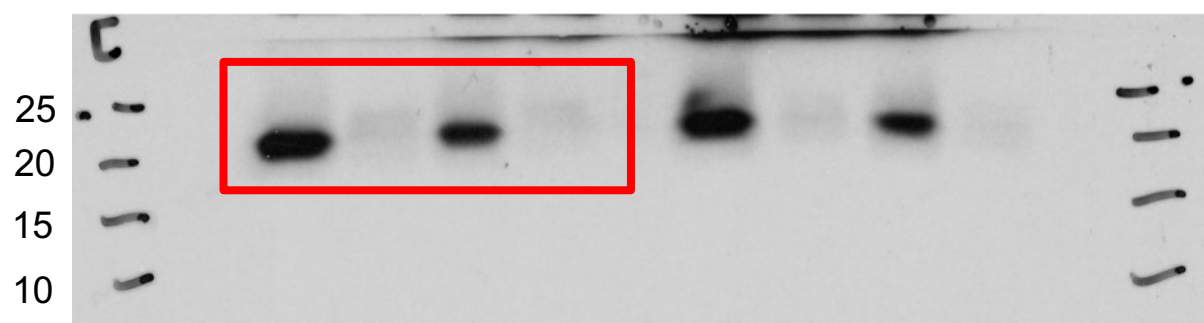

p-4EBP1

EZH2i (Taz): -- -- + + | Lanes not presented in figure
mTORC1i (RMC): -- + -- + | X

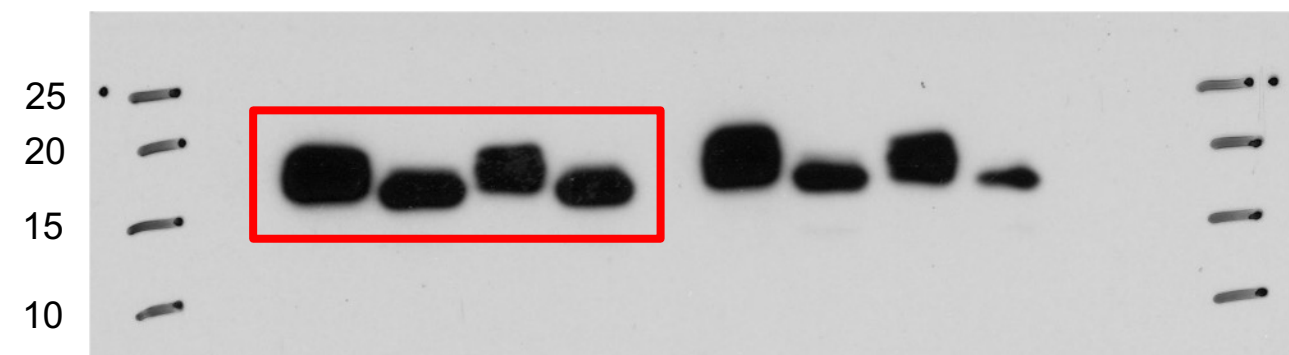

4EBP1

EZH2i (Taz): -- -- + + | Lanes not presented in figure
mTORC1i (RMC): -- + -- + | X

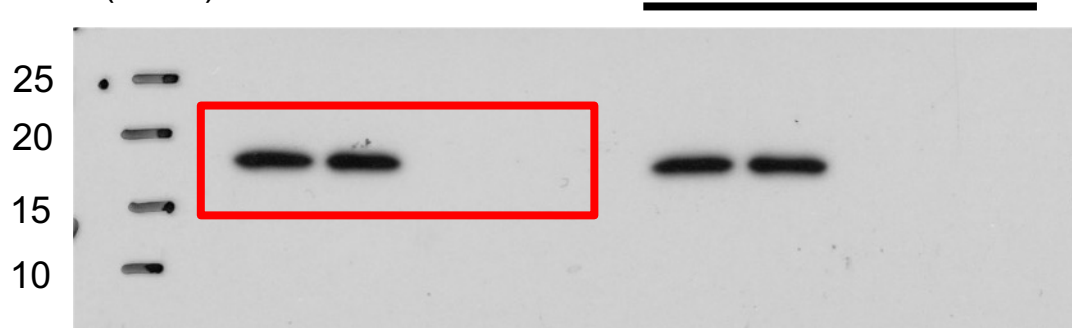

H3K27me3

EZH2i (Taz): -- -- + + | Lanes not presented in figure
mTORC1i (RMC): -- + -- + | X

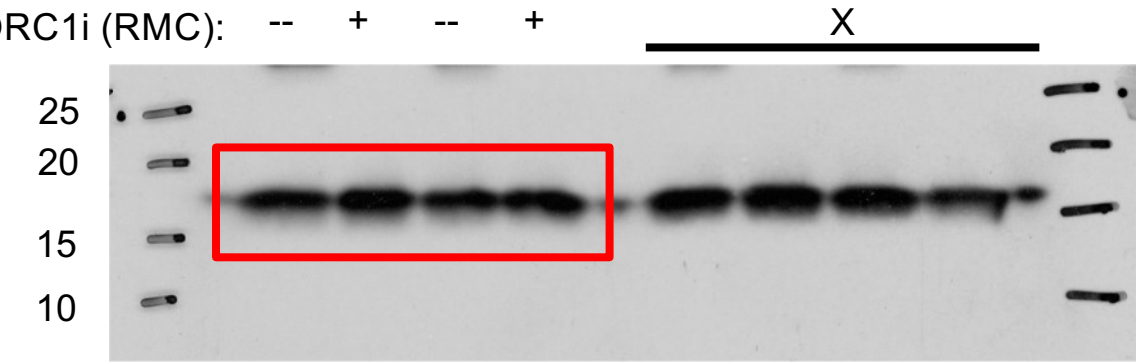

H3

EZH2i (Taz): -- -- + + | Lanes not presented in figure
mTORC1i (RMC): -- + -- + | X

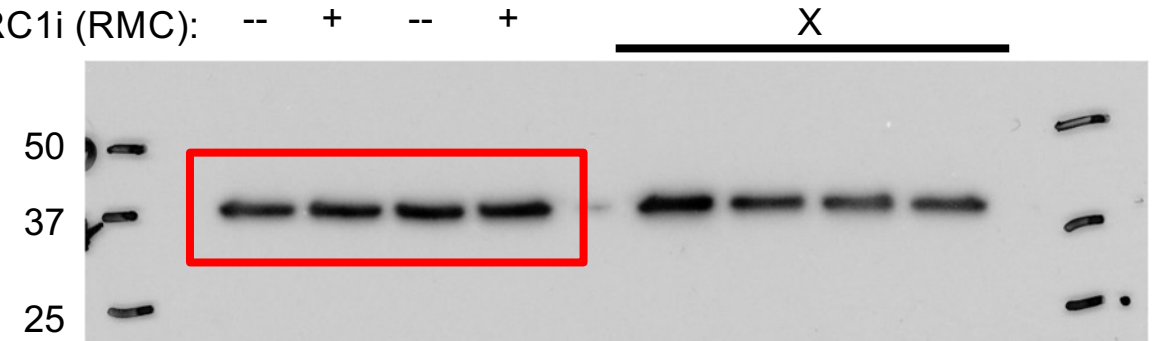

GAPDH

Raw unedited blots for Supplemental Figure 1H

EEDi (MAK):	--	--	--	--	+	+
EZH2i (Taz):	--	--	+	+	--	--
AKTi (Ipata):	--	+	--	+	--	+

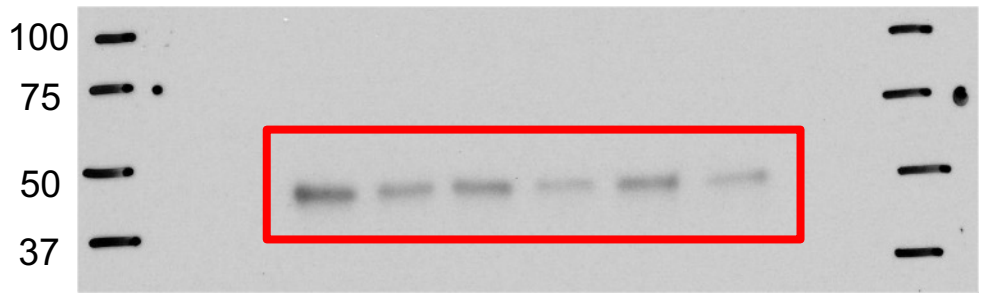

p-PRAS40

EEDi (MAK):	--	--	--	--	+	+
EZH2i (Taz):	--	--	+	+	--	--
AKTi (Ipata):	--	+	--	+	--	+

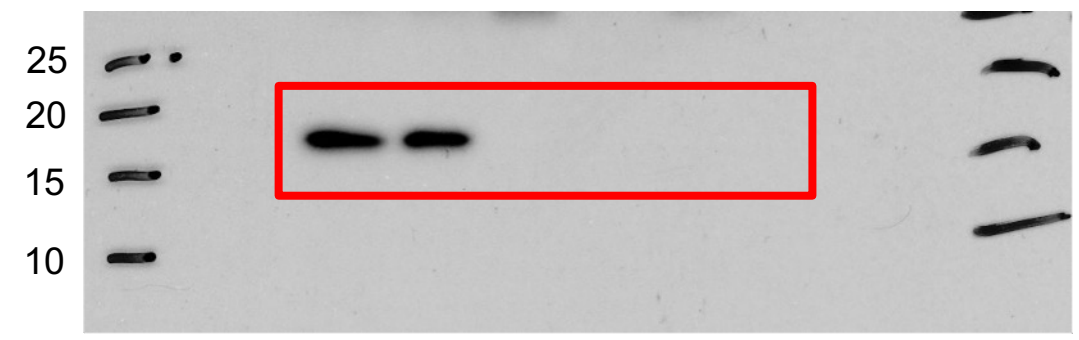

H3K27me3

EEDi (MAK):	--	--	--	--	+	+
EZH2i (Taz):	--	--	+	+	--	--
AKTi (Ipata):	--	+	--	+	--	+

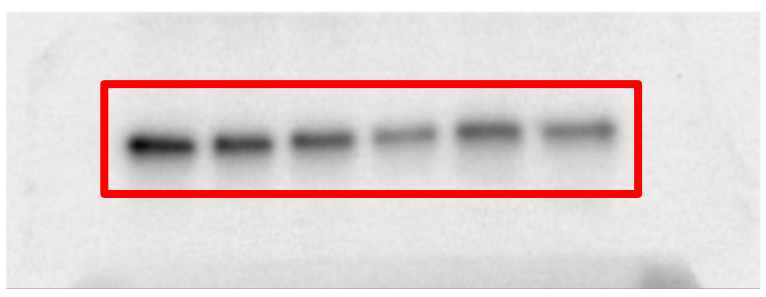

Merged with light image of ladder

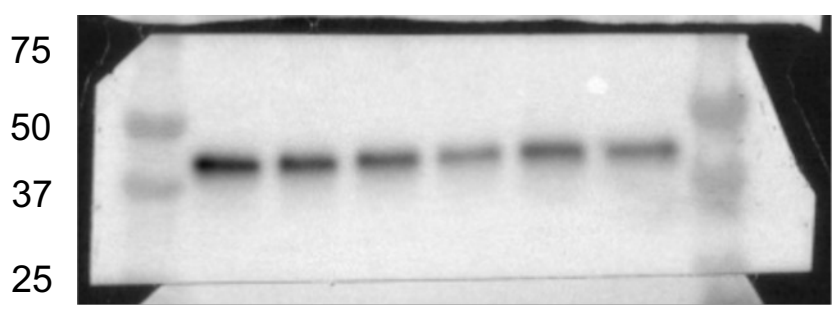

PRAS40

EEDi (MAK):	--	--	--	--	+	+
EZH2i (Taz):	--	--	+	+	--	--
AKTi (Ipata):	--	+	--	+	--	+

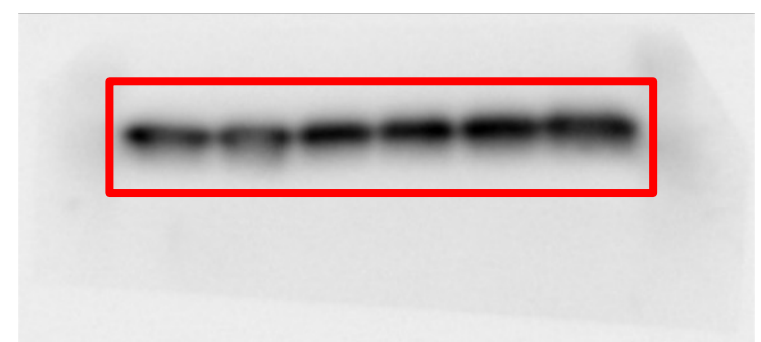

Merged with light image of ladder

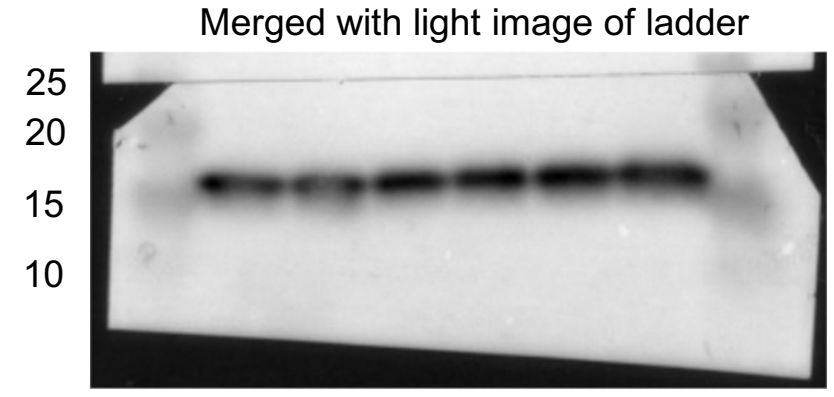

H3

EEDi (MAK):	--	--	--	--	+	+
EZH2i (Taz):	--	--	+	+	--	--
AKTi (Ipata):	--	+	--	+	--	+

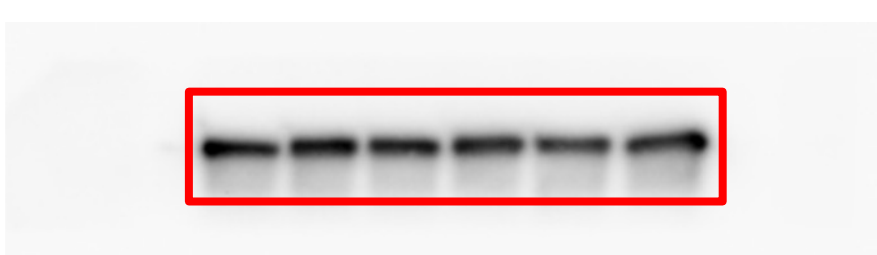

Merged with light image of ladder

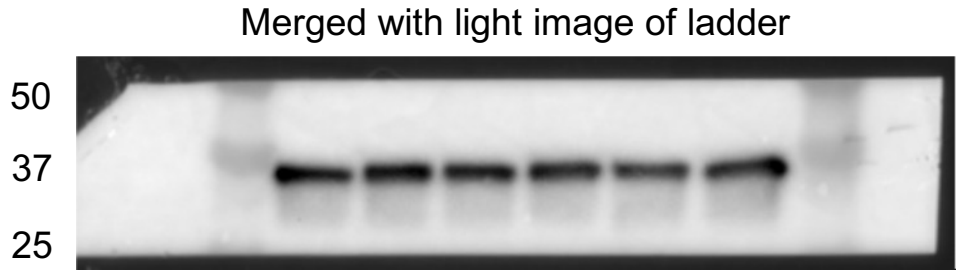

GAPDH

Raw unedited blots for Supplemental Figure 3C

Note: HK2 blot is from the same experiment and has the same GAPDH membrane as Figure 1D

EZH2i (Taz): -- -- + +
AKTi (Ipata): -- + -- +
Lanes not presented in figure X

250
150
100
75

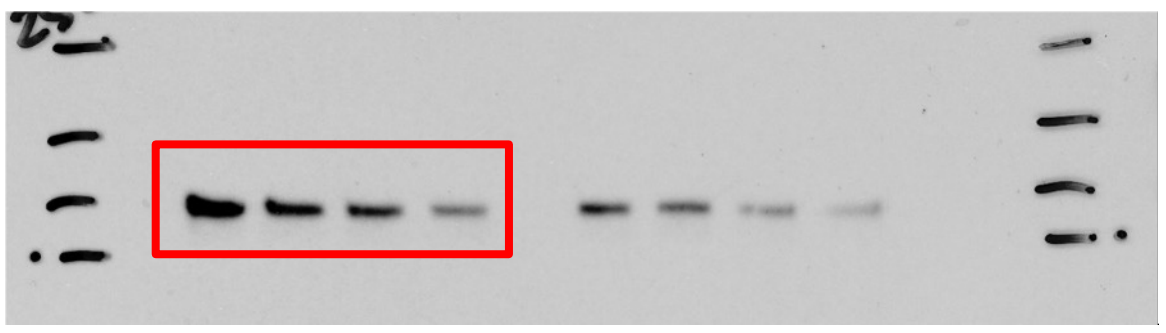

HK2

EZH2i (Taz): -- -- + +
mTORC1i (RMC): -- + -- +
Lanes not presented in figure X

100
75
50
37
25

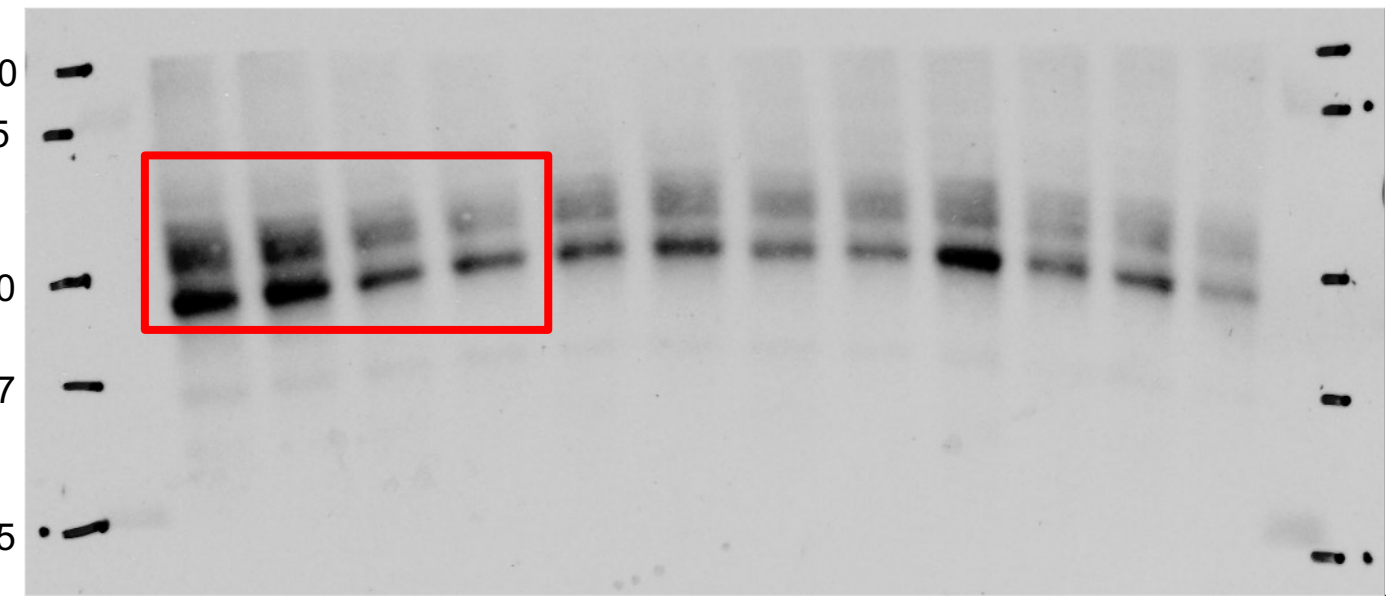

GLUT1

EZH2i (Taz): -- -- + +
AKTi (Ipata): -- + -- +
Lanes not presented in figure X

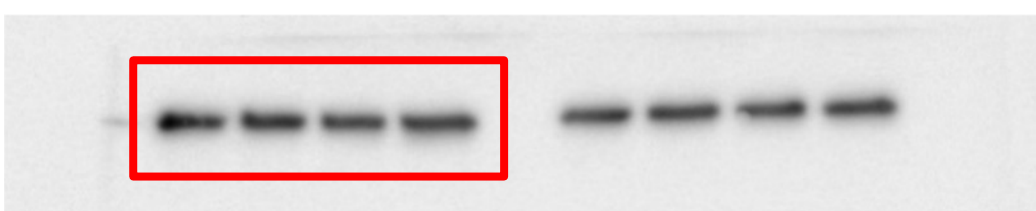

Merged with light image of ladder

50
37
25

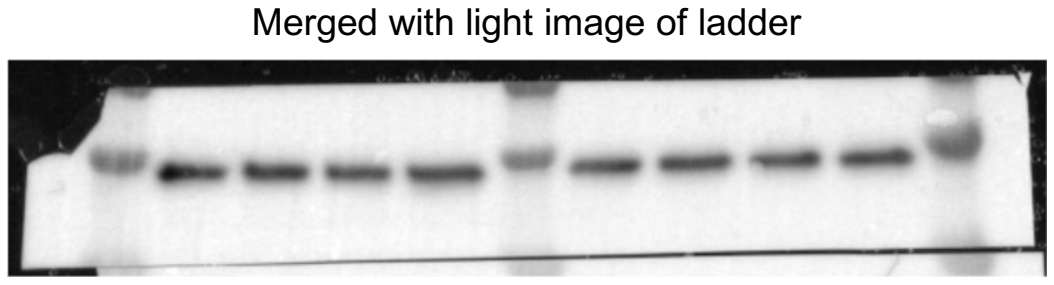

GAPDH

EZH2i (Taz): -- -- + +
mTORC1i (RMC): -- + -- +
Lanes not presented in figure X

50
37
25

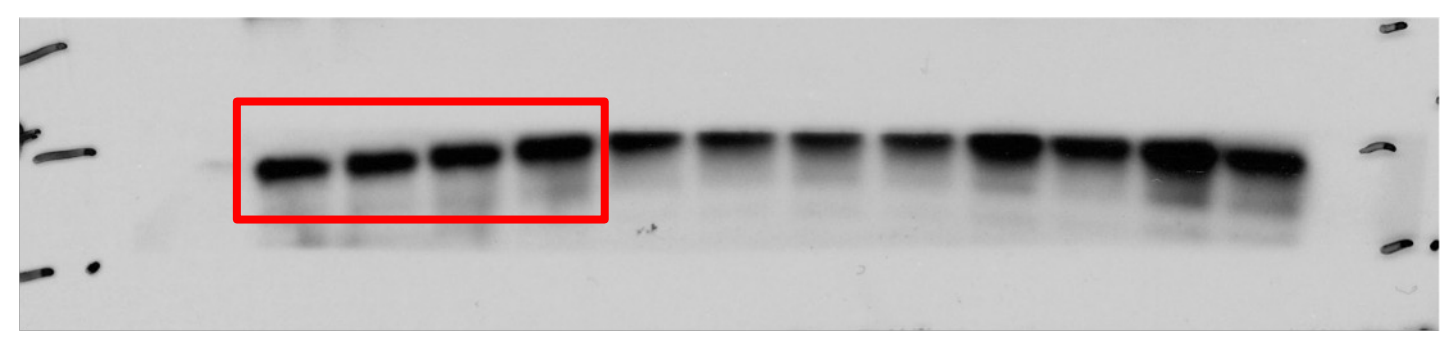

GAPDH

Raw unedited blots for Supplemental Figure 4A

Note: these blots are from the same experiment and have the same GAPDH membranes as Figure 1D

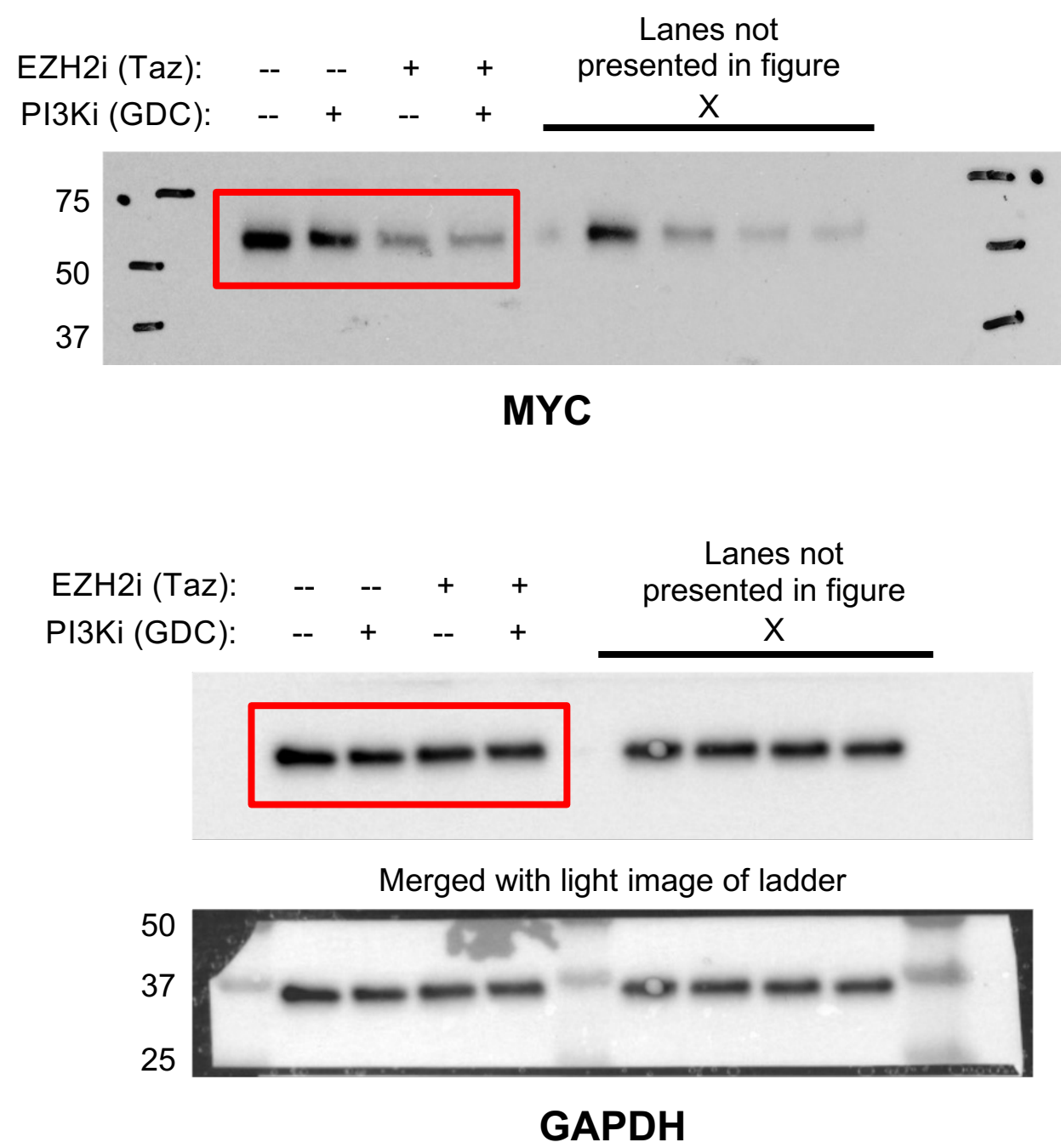

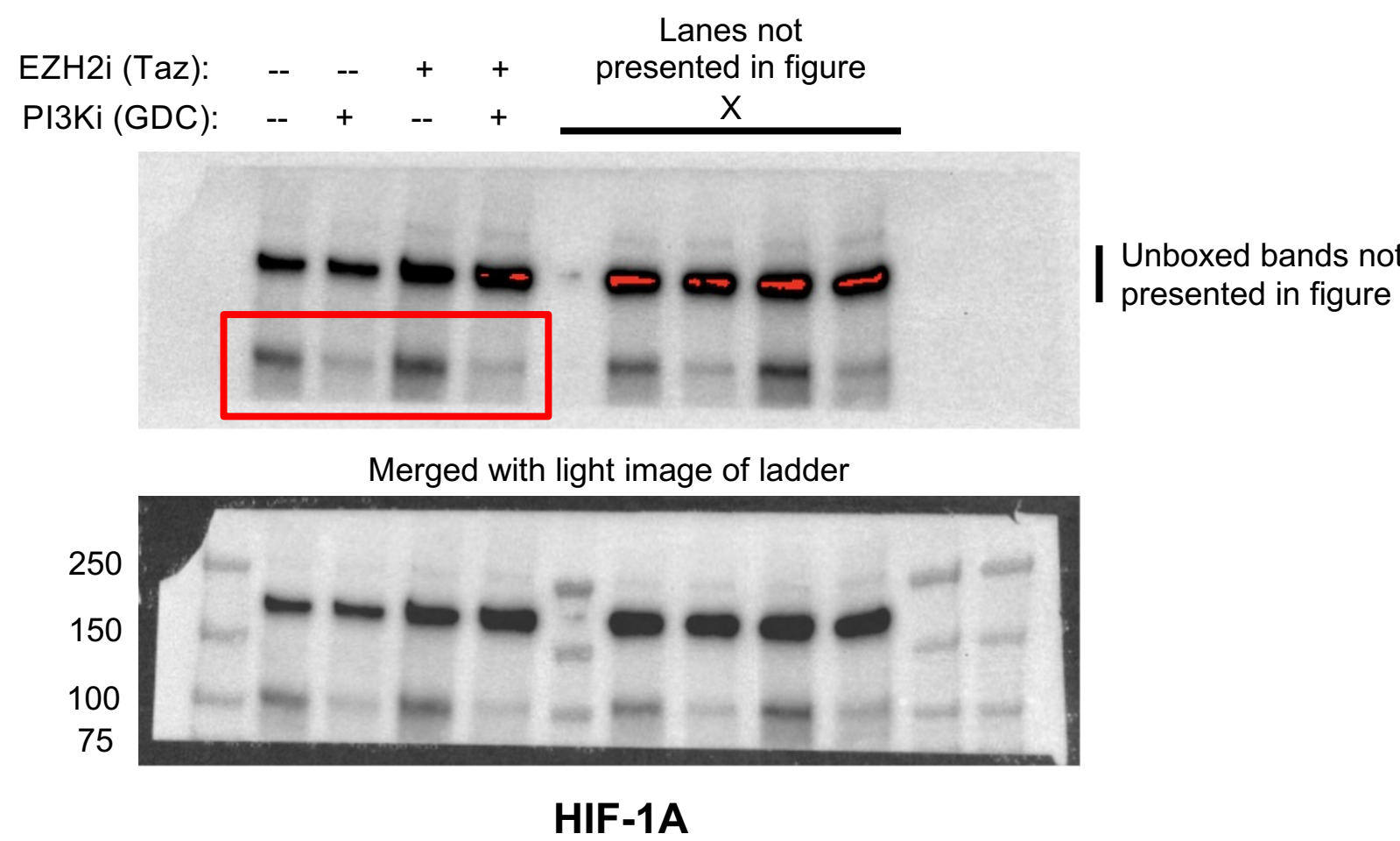

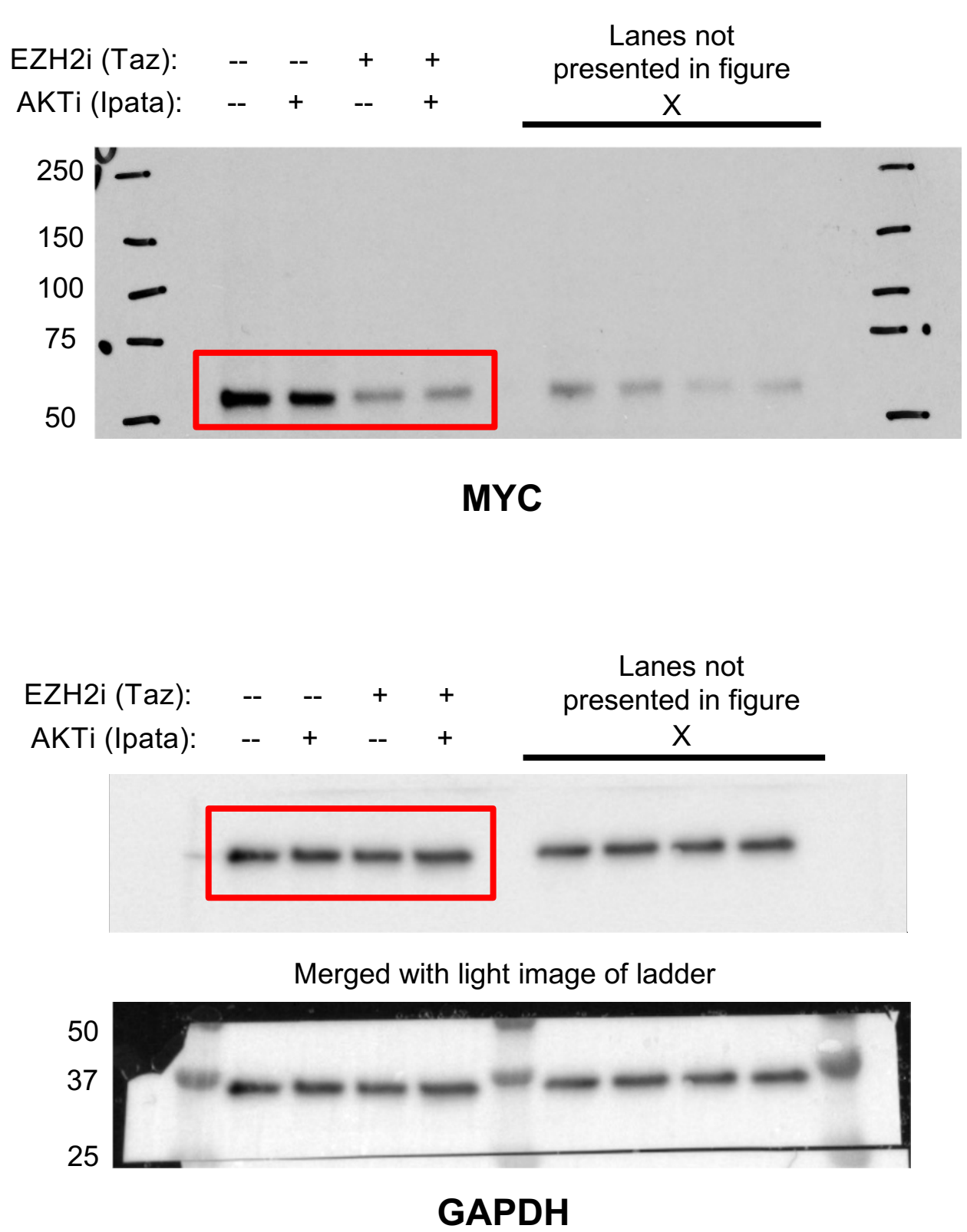

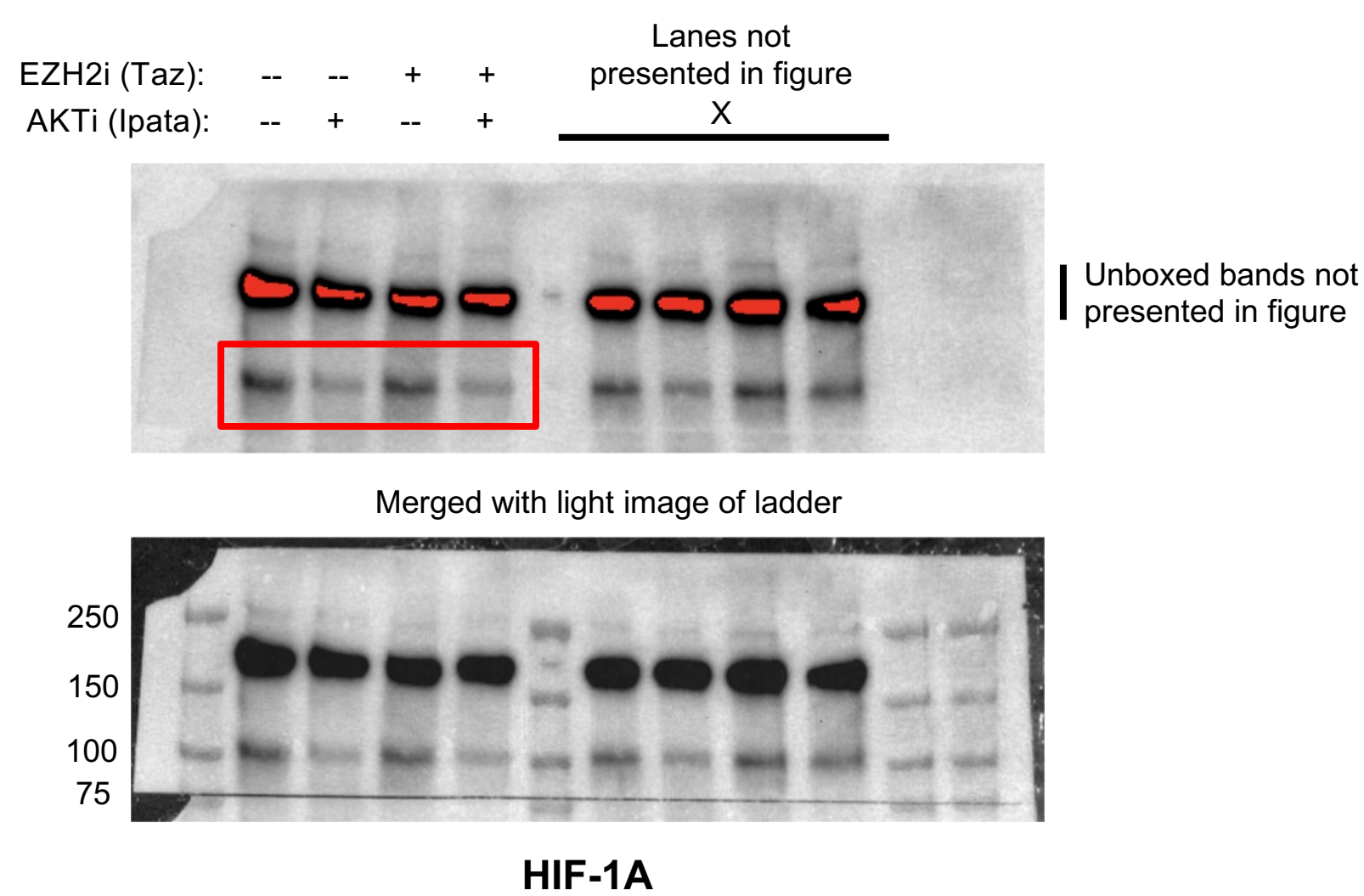

Raw unedited blots for Supplemental Figure 4B

Note: these blots are from the same experiment and have the same GAPDH membrane as Figure 1D

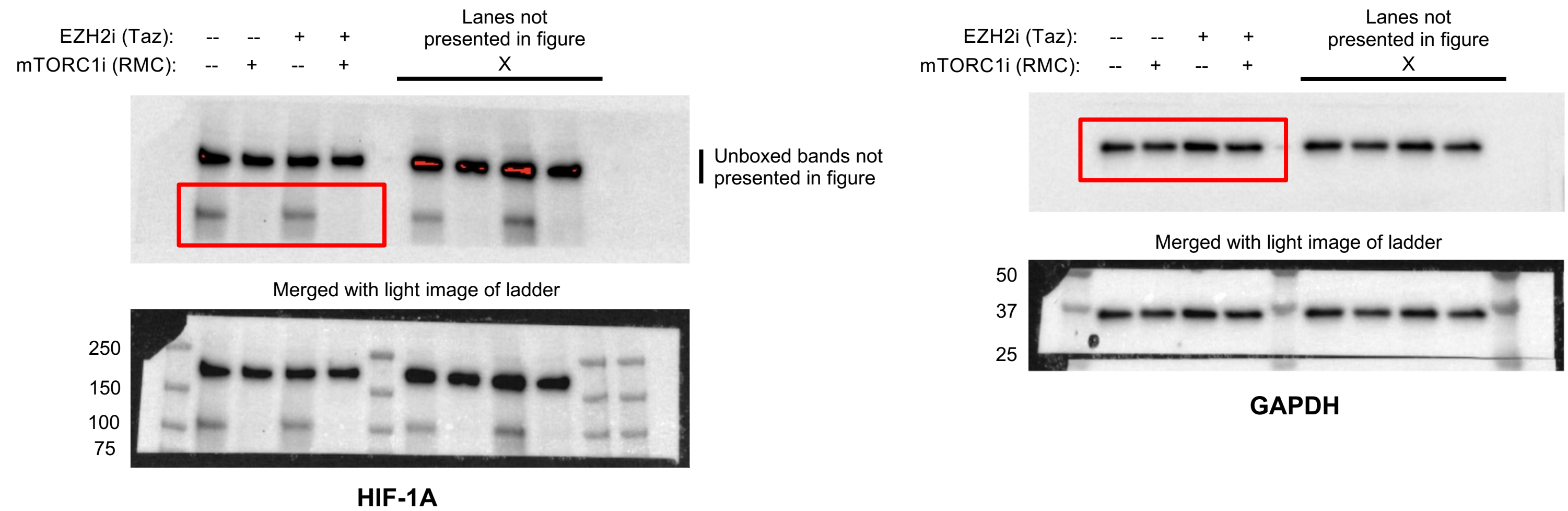

Raw unedited blots for Supplemental Figure 4C

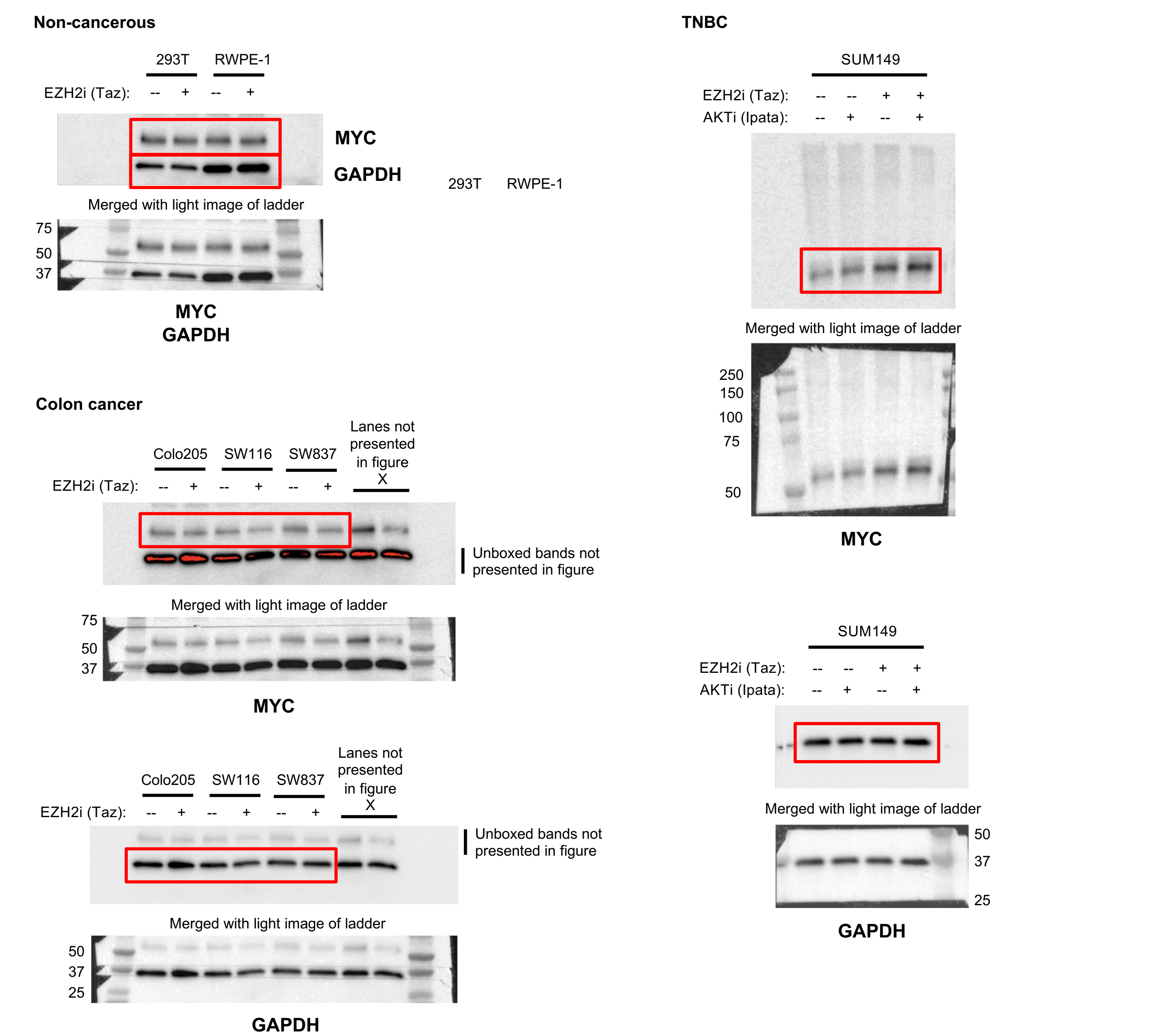

Raw unedited blots for Supplemental Figure 5C

Note: same experiment and GAPDH membrane as Figure 1D, but different lanes were used

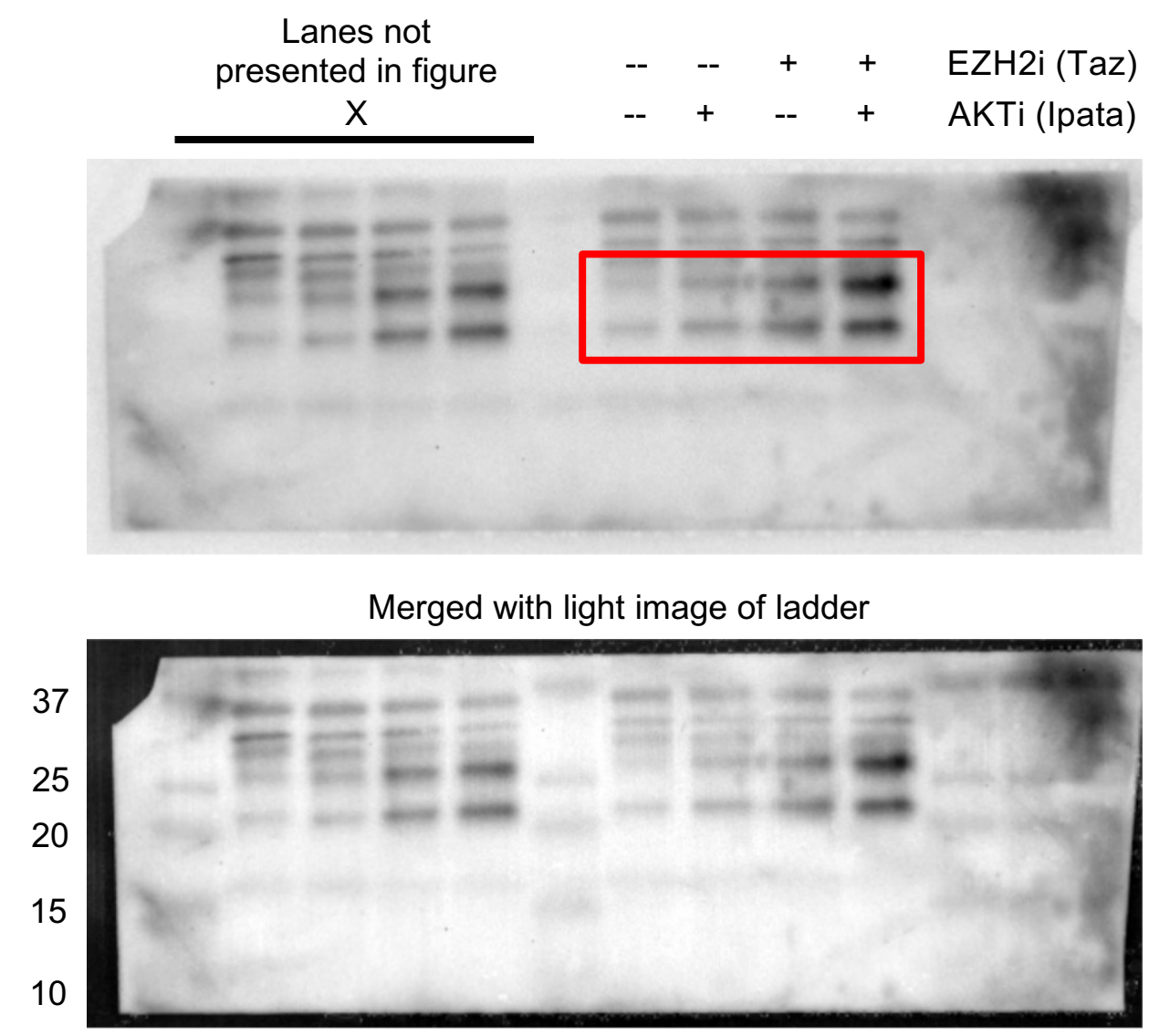

BMF

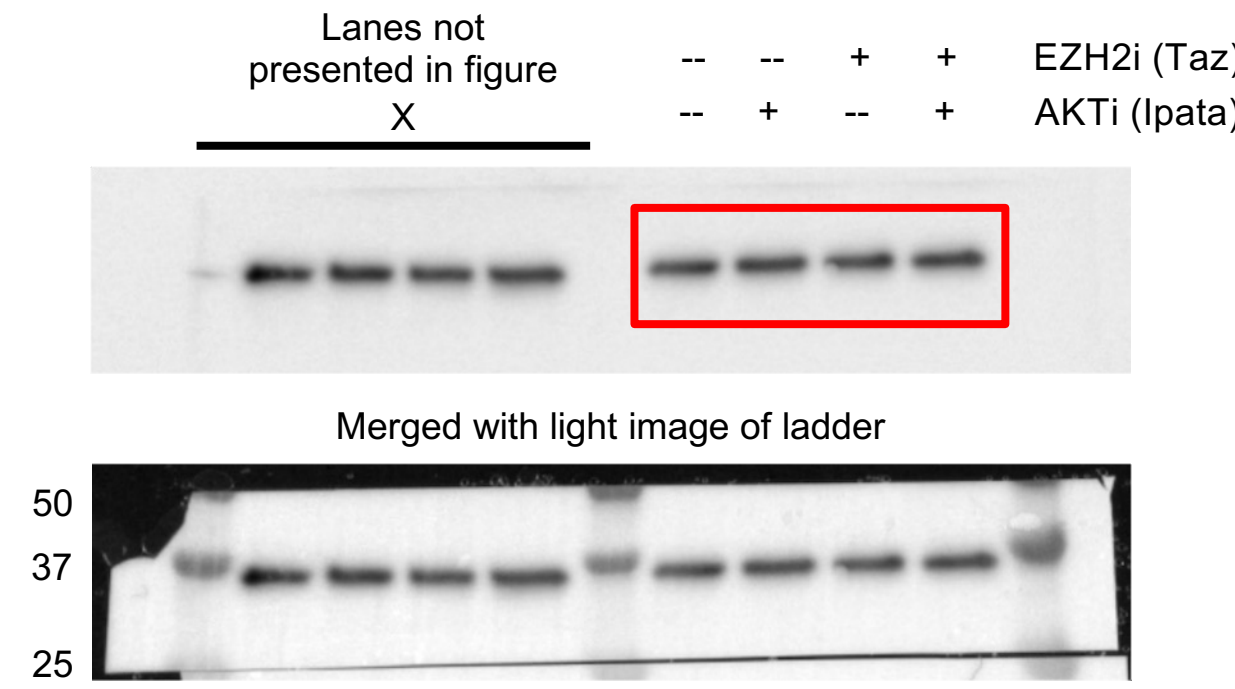

GAPDH

Raw unedited blots for Supplemental Figure 5D

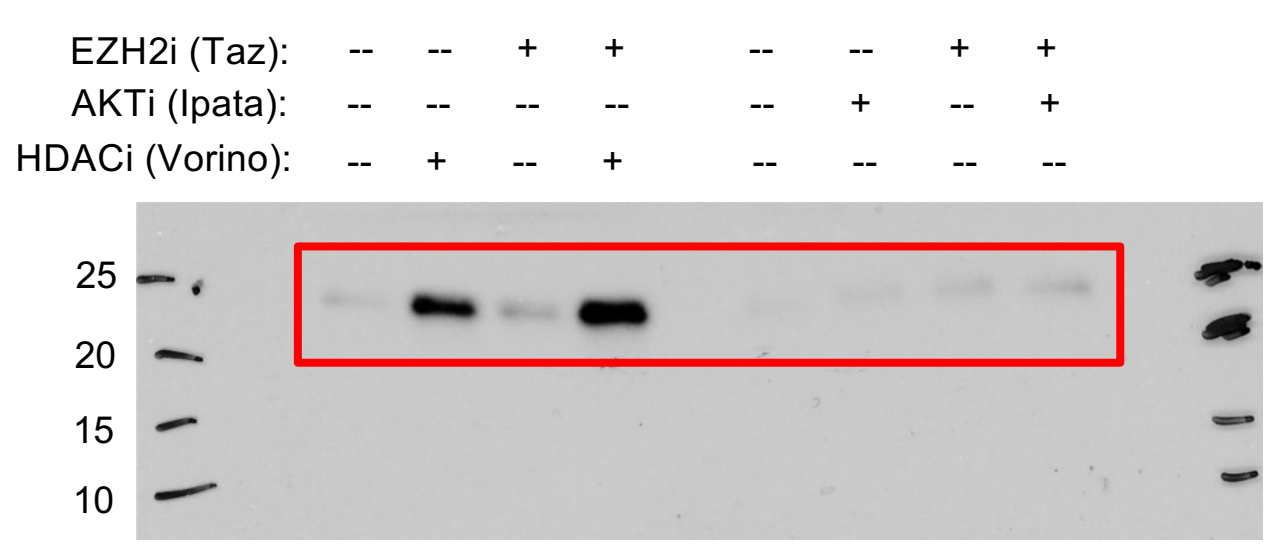

ATF3

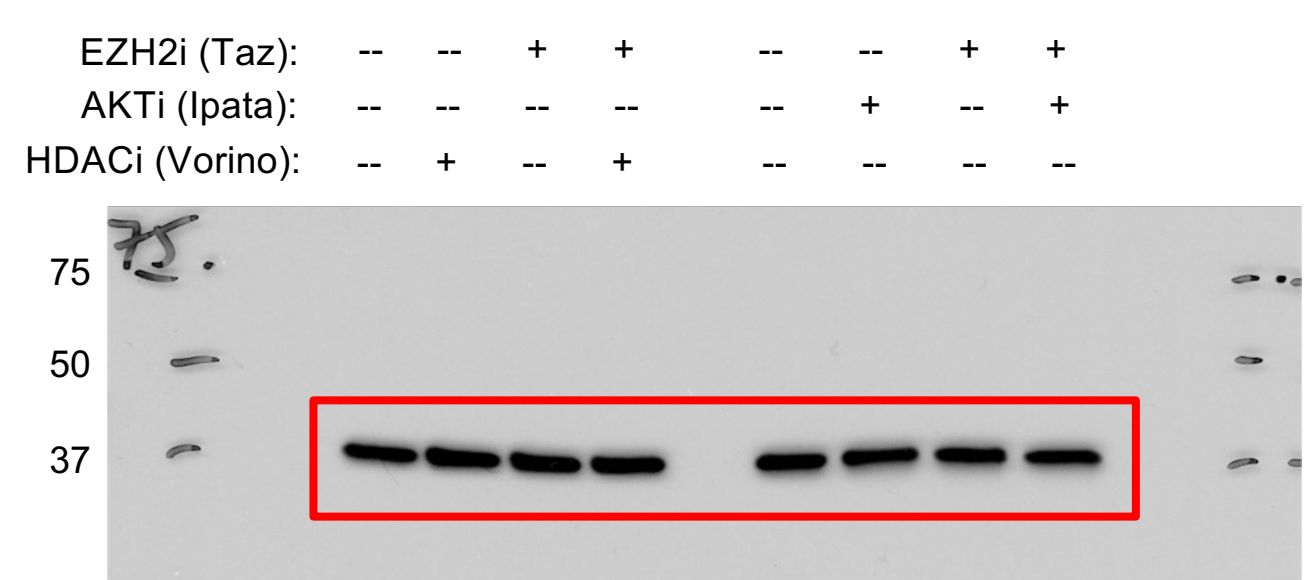

GAPDH

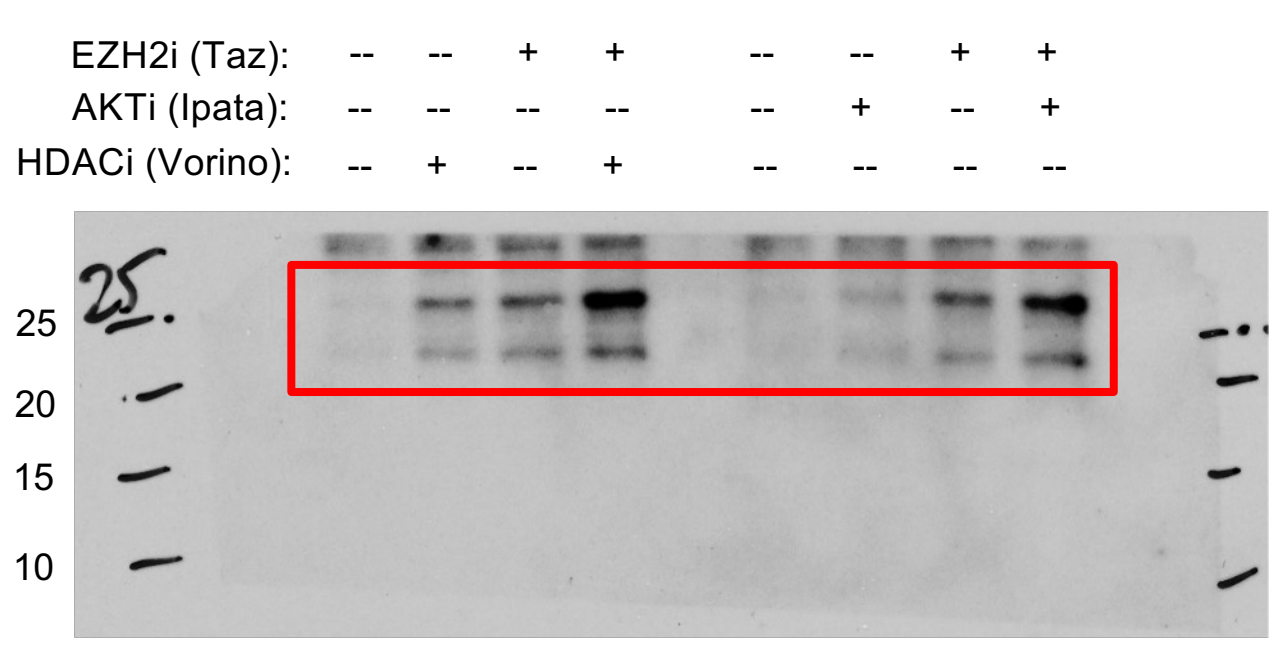

BMF
